# Carbon Nanotube Assembly and Integration for Applications

**DOI:** 10.1186/s11671-019-3046-3

**Published:** 2019-07-01

**Authors:** Anusha Venkataraman, Eberechukwu Victoria Amadi, Yingduo Chen, Chris Papadopoulos

**Affiliations:** Department of Electrical and Computer Engineering, University of Victoria, P.O. Box 1700 STN CSC, Victoria, BC V8W 2Y2 Canada

**Keywords:** Carbon nanotubes, Chemical vapor deposition, Catalyst patterning, Self-assembly, Integration, Electronics

## Abstract

Carbon nanotubes (CNTs) have attracted significant interest due to their unique combination of properties including high mechanical strength, large aspect ratios, high surface area, distinct optical characteristics, high thermal and electrical conductivity, which make them suitable for a wide range of applications in areas from electronics (transistors, energy production and storage) to biotechnology (imaging, sensors, actuators and drug delivery) and other applications (displays, photonics, composites and multi-functional coatings/films). Controlled growth, assembly and integration of CNTs is essential for the practical realization of current and future nanotube applications. This review focuses on progress to date in the field of CNT assembly and integration for various applications. CNT synthesis based on arc-discharge, laser ablation and chemical vapor deposition (CVD) including details of tip-growth and base-growth models are first introduced. Advances in CNT structural control (chirality, diameter and junctions) using methods such as catalyst conditioning, cloning, seed-, and template-based growth are then explored in detail, followed by post-growth CNT purification techniques using selective surface chemistry, gel chromatography and density gradient centrifugation. Various assembly and integration techniques for multiple CNTs based on catalyst patterning, forest growth and composites are considered along with their alignment/placement onto different substrates using photolithography, transfer printing and different solution-based techniques such as inkjet printing, dielectrophoresis (DEP) and spin coating. Finally, some of the challenges in current and emerging applications of CNTs in fields such as energy storage, transistors, tissue engineering, drug delivery, electronic cryptographic keys and sensors are considered.

## Introduction

Carbon nanotubes (CNTs) are long, hollow cylindrical tubule structures made of graphite sheets (a.k.a. graphene), with diameters ranging from below 1 nm to 10 s of nm [1]. CNTs exhibit different electronic properties based on the way these graphene layers are rolled into a cylinder. Nanotubes could either be single-walled structures, called single-walled carbon nanotubes (SWCNTs) or could have many walls, called multi-walled carbon nanotubes (MWCNTs). SWCNTs can be further categorized electrically into semiconducting and metallic SWCNTs (s-SWCNTs and m-SWCNTs), while MWCNTs mainly display metallic behavior. The novel and useful properties of CNTs, such as low-cost, light-weight, high aspect ratios and surface area, distinct optical characteristics, high thermal and electrical conductivity and high mechanical strength make them suitable and of interest for a wide range of electronic, biomedical and other industrial applications. For example, CNTs are promising for electronics ‘beyond CMOS’ as active devices and interconnects in future integrated circuits [2].

CNTs are part of the fullerene family, which are a group of carbon allotropes with atoms linked in the shape of cage-like structures such as a hollow sphere, ellipsoid or cylindrical tube [3]. Fullerenes are comprised of graphene sheets of linked hexagonal and pentagonal rings, which give them their curved structure. Graphene is an allotrope of carbon, which is comprised of a single layer of carbon atoms, arranged in a two-dimensional hexagonal lattice. It is a semi-metal, which has an overlap between the valence and conduction bands, i.e. it has a zero-bandgap [1]. The buckminsterfullerene (buckyball/C_60_), one of the most common spherical fullerenes, is a nanoscale molecule having 60 carbon atoms, with each atom being bonded to three other adjacent atoms to form hexagons and pentagons, with the ends curved into a sphere. The C_70_ molecule is another spherical fullerene that is known for being chemically stable. Additionally, other smaller metastable species, such as C_28_, C_36_ and C_50_, have been discovered. It is believed that fullerenes have existed in nature for a long time; minute quantities of fullerenes in the form of C_60_, C_70_, C_76_, C_82_ and C_84_, have been found hidden in soot [3, 4]. Nanotubes are comprised of sp^2^-hybridized carbon bonds, which are stronger than the sp^3^-hybridized carbon bonds found in diamond, thereby making for the exceptional strength and stiffness of nanotubes. Additionally, they are known to possess very high electrical conductivity [5, 6], high charge carrier mobility [7], high chemical stability [8, 9], large specific surface ratio [10], high aspect ratio [11], excellent mechanical properties [12, 13] and excellent heat conductivity [14], with some SWCNTs exhibiting superconductivity [15, 16]. These properties make CNTs an important topic in nanoscience and electronics research [17].

The specific surface area (SSA) of an individual SWCNT has been theoretically obtained as 1315 m^2^ g^−1^; but measured surface areas are much lower due to bundling, agglomeration and purity of the tubes [10]. For example, SWCNT specimens with SSA values between 150 and 790 m^2^ g^−1^ have been obtained [10]. For MWCNTs, the number of walls is the predominant parameter which determines the SSA. Some measured SSA values include 680–850 m^2^ g^−1^ for two-walled CNTs and 500 m^2^ g^−1^ for three-walled CNTs [10]. Additionally, CNTs have noteworthy mechanical properties. The elastic modulus for individual MWCNTs is about 1 TPa, while the tensile strength for MWCNTs ranges from 11 to 63 GPa [12, 13]. On the other hand, for single SWCNTs, tensile strength values of about 22 GPa have been obtained [12]. Young’s modulus of individual SWCNTs was directly measured and estimated to be between 0.79 and 3.6 TPa [12, 13, 18] while for individual MWCNTs, values of between 0.27 and 2.4 TPa were obtained [12, 19]. The compressive strength of thin MWCNTs was estimated to be between 100 and 150 GPa [20]. CNTs also have good thermal properties. Individual SWCNTs can have thermal conductivity values between 3500 and 6600 W m^−1^ K^−1^ at room temperature, which exceeds the thermal conductivity of diamond [14, 21], while the thermal conductivity of individual MWCNTs ranges from 600 to 6000 W m^−1^ K^−1^ [21]. CNTs also have interesting dimensional properties. Their aspect ratio (length-to-diameter) values can be extremely high. Typical CNT diameter values vary from 0.4 to 40 nm (i.e. by about 100 times), but the length can vary by 10,000 times, reaching 55.5 cm, thus the aspect ratio can be very high [11].

**Fig. 1:**
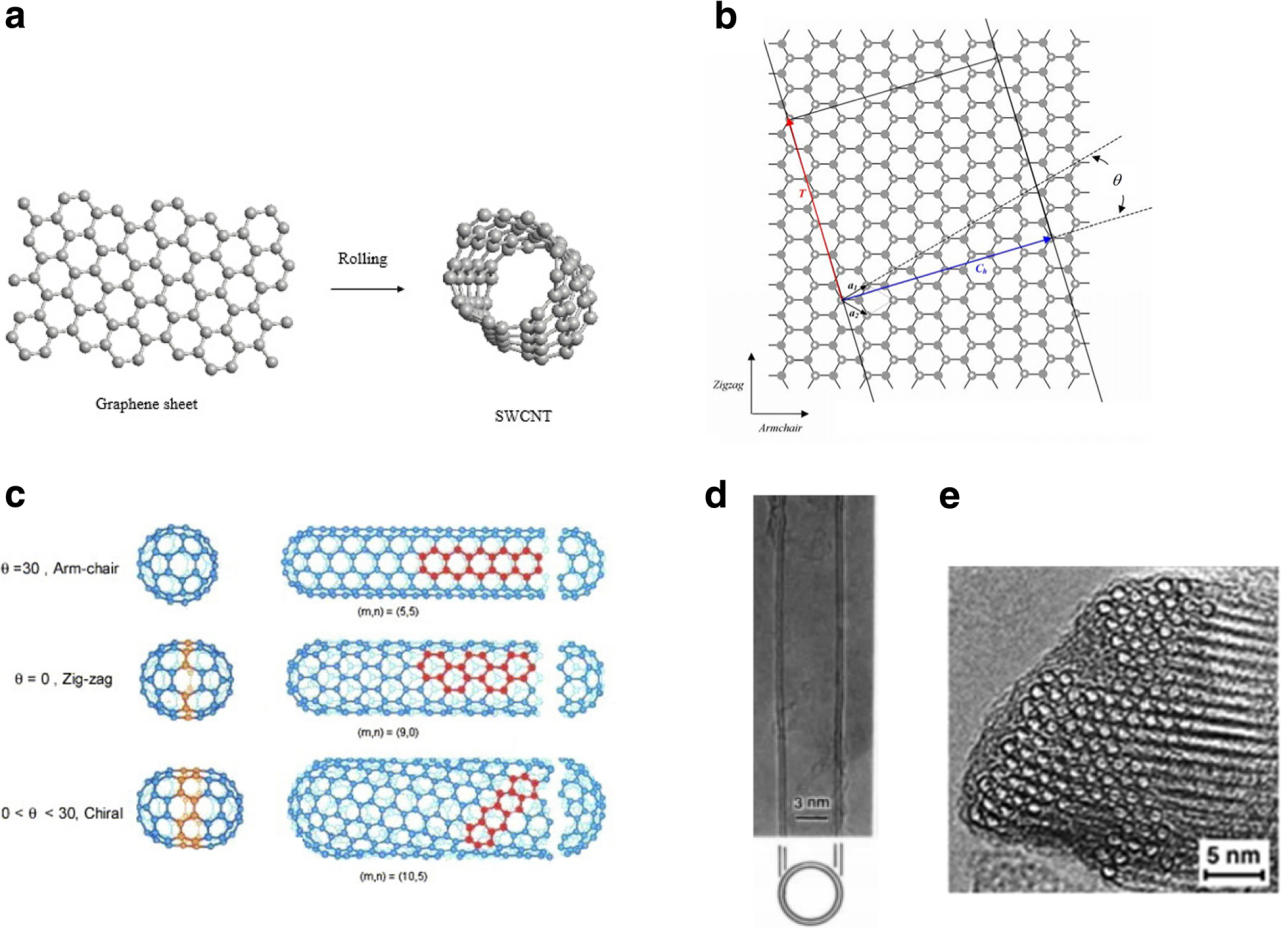
**a** Formation of SWCNT by rolling a single layer of graphite. **b** Illustration of forming a CNT from an ideal graphite sheet. The two ends of the chiral vector *C*_*h*_ are superimposed to create a nanotube with 1D lattice vector *T* and chiral angle *ϴ*. *a*_1_ and *a*_2_ are the primitive lattice vectors of 2D graphite (white dots denote lattice pints). The zigzag and armchair wrapping directions are also indicated. **c** Different types of CNTs based on their chirality. Adapted from [22]. **d** Electron micrograph image of a double-walled CNT with a diameter of 5.5 nm. Adapted from [78]. **e** Electron microscope images of a bundle of ~ 100 SWCNTs, packed in a triangular lattice. Adapted from [17]

CNTs also have unique electronic properties. CNTs’ distinct electronic properties are inherently related to their unique, low-dimensional band structure and quantum-confined carriers. SWCNTs can be either metallic or semiconducting, depending on the diameter and orientation of the graphene lattice with respect to the tube axis, termed as the chirality of the tube [1, 22]. Basis vectors *a*_1_ and *a*_2_ determine the graphene lattice. The chiral vector (***C***), which corresponds to the side of the graphene sheet that will eventually become the CNT circumference is given by: *C* = *na*_1_ + *ma*_2_, where the *n* and *m* are integers. The graphene sheets can be rolled in different ways to generate the three different classes of SWCNTs, as shown in Fig. 1a–c. Also, the electronic properties of each CNT arise from the geometry of the tube, dictated by its chiral vectors. If *m = 0*, *C* lies along either *a*_1_ or *a*_2_ and the nanotubes will be referred to as zigzag CNTs, while if *n* = *m*, *C* lies along the direction exactly between *a*_1_ and *a*_2_ and the tubes are referred to as armchair nanotubes. Finally, chiral nanotubes are formed when *n ≠ m*. Figure 1b shows how the different types of CNTs based on the chiral indices and the corresponding chiral angles are defined. Analysis within the so-called zone-folding scheme [23] shows that armchair tubes are always metallic while two-thirds of zigzag tubes are semiconducting. More generally, two-thirds of all SWNTs are predicted to be semiconducting with the rest metallic or possessing a small band gap (quasi-metallic).

**Fig. 2:**
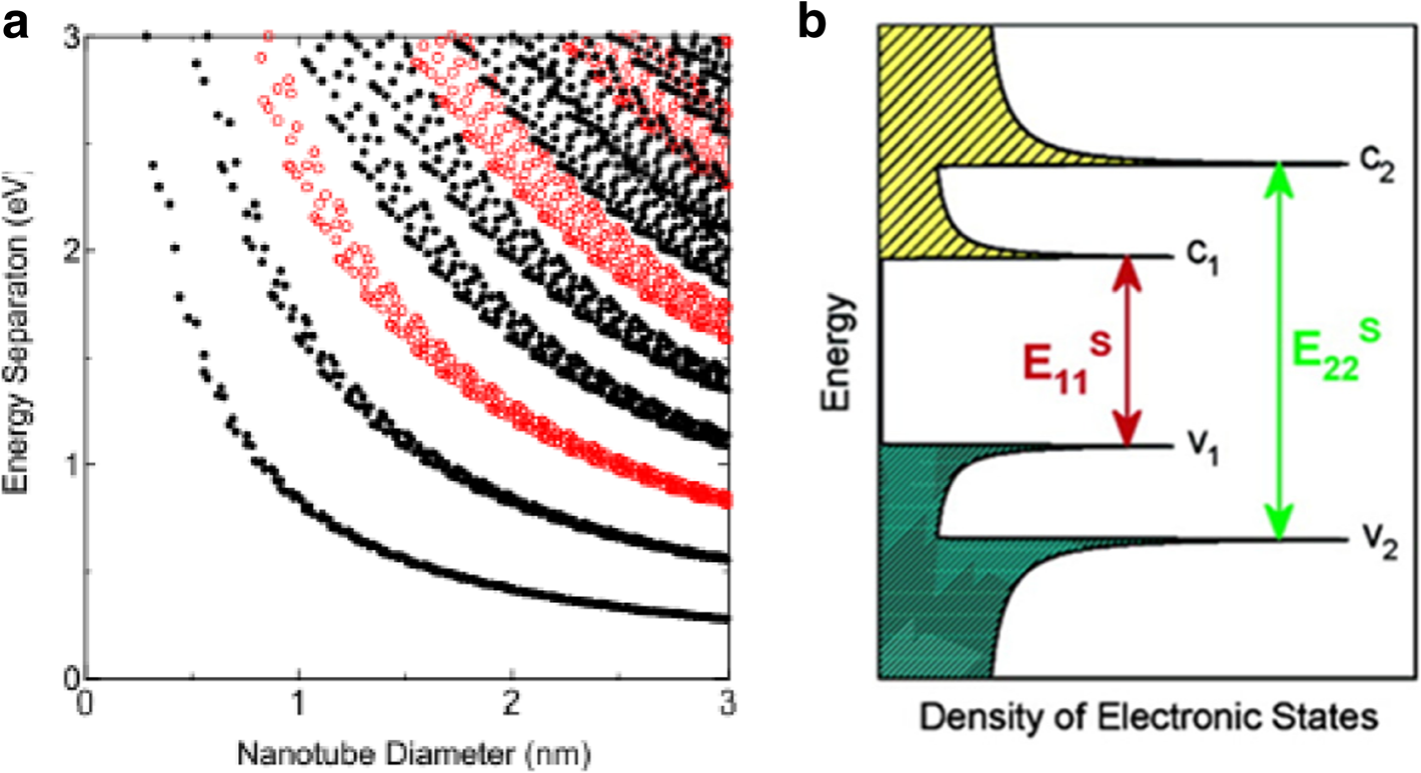
**a** Kataura plot relating the energy of the band gaps in a carbon nanotube and its diameter. Here, red circles denote semiconducting CNTs and black circles denote metallic CNTs. Adapted from [32]. **b** Schematic showing the density of states and VHS peaks (indicated by a sharp maxima) of a semiconducting CNT. Arrows indicate the mechanism of light absorption and emission. Adapted from [36]

CNTs have extremely high charge carrier mobility, and as such, they have the potential to be considered for various electronic device applications [24]. Much progress has been made showing that SWCNTs are advanced quasi-one-dimensional (1D) materials, with high carrier mobility. Estimated values of the carrier mobility in CNTs range from 20 cm^2^ V^−1^ s^−1^ [7] to very large values (~ 10^4^ or greater) in semiconducting tubes and ballistic in metallic tubes [25]. Current densities of between 10^7^ A cm^−2^ and 10^8^ A cm^−2^ are achievable for SWCNTs, with SWCNTs being able to pass currents of about 20 μA [26]. Ballistic SWCNTs with have shown resistances between 6.5 and 15 kΩ. MWCNTs are typically metallic [1], and have a very high current-carrying capacity ranging from 10^6^ to 10^9^ A cm^−2^ [26, 27]. The bandgap of a semiconducting CNT has been shown to be inversely proportional to its diameter (Fig. 2a), and is given by *E*_gap_ = *2γ*_*0*_*a*_*C-C*_*/d*, where *y*_*o*_ represents the C-C tight binding overlap energy (2.45 eV), *a*_*C-C*_ is the nearest neighbor C-C distance (0.142 nm) and *d* is the diameter of the tube [28, 29]. For example, semiconducting CNTs with a radius of 0.2 nm have band gaps of about 2.2 eV, while tubes with a radius of 1.4 nm have band gaps of about 0.4 eV [30].

Each CNT has a distinct optical property because the wave function boundary condition alters with the (*n*, *m*) indices or chirality of the tube. Thus, optical properties such as absorption, photoluminescence and Raman spectroscopy can be used to extensively carry out quick and non-destructive studies of CNTs, by probing CNT samples with photons [31–33]. CNTs also exhibit unique photo-ignition properties when exposed to light [34, 35], resulting in the generation of an acoustic wave and oxidation of the CNTs. The results of optical spectroscopies can be recorded by a Kataura plot, in which each point represents the optical transition energy *E*_*ii*_ (*i* = 1, 2, 3, ...) for a specific (*n, m*) SWCNT plotted as a function of the tube diameter as shown in Fig. 2a. 1D crystals do not have their density of states (DOS) as a continuous function of energy, but have a spike-like DOS, which rises and falls in a discontinuous spike. These sharp spikes or Van Hove singularities (VHS) make for the unique optical properties of CNTs [32]. Optical absorption in CNTs is different from absorption in most bulk materials due to the presence of sharp peaks. When SWCNTs absorb light, the electrons in the VHS of the valence band are elevated to the corresponding energy levels in the conduction band. In nanotubes, optical absorption is tied to the sharp electronic transitions from the *v*_2_ to *c*_2_ (energy *E*_*22*_) or *v*_1_ to *c*_1_ (*E*_11_) levels (Fig. 2b) [36]. These transitions are probed and are then used to identify nanotube types [32].

Apart from optical absorption properties of CNTs, another optical property that is typically studied is its photoluminescence. Photoluminescence is used to measure the quantities of semiconducting nanotube species in a sample of CNTs. Semiconducting SWCNTs emit near-infrared light when excited by a photon, a property referred to as photoluminescence [37]. When an electron in a semiconducting SWCNT absorbs excitation light, resulting in an *E*_22_ transition (electronic transition from the valence to conducting band in a semiconducting SWCNT), an electron-hole pair is created. Both the electron and hole rapidly relax, from *c*_2_ to *c*_1_ and from *v*_2_ to *v*_1_ states, respectively. Finally, they recombine through a *c*_1_ − *v*_1_ transition resulting in light emission [32]. No excitonic luminescence can be produced in metallic tubes—although they can produce electron-hole pairs, the holes are immediately filled by other electrons out of the many available in the metal and hence no excitons are produced.

Raman spectroscopy, another optical technique for CNT characterization, has the ability to detect semiconducting as well as metallic tubes [38] and via Raman microscopy can provide good spatial resolution as well. In Raman spectroscopy, a photon is used to excite a sample of CNTs and is scattered by the phonons in the sample. An analysis of the change in frequency between the exciting photon and the released photon tells what kind of CNTs are in a sample, mainly via the diameter-dependent radial breathing mode [23]. Raman scattering in SWCNTs can also be resonant, meaning that only tubes which have one of the bandgaps equal to the exciting laser energy are selectively probed with an enhanced absorption cross-section.

Selected numerical data for the CNT properties described above are listed in Table 1:

**Table 1:** Tabular representation of CNT properties

Property (unit)	SWCNT	MWCNT	References
Diameter (nm)	0.4–3	1.4–100	[39]
Aspect ratio	4300	1250–3750	[40]
Specific surface area (m^2^ g^−1^)	150–790	50–850	[10]
Tensile strength (GPa)	22 ± 2	11–63	[12, 13]
Young’s modulus (TPa)	0.79–3.6	0.27–2.4	[12, 13, 18, 20]
Thermal conductivity (W m^−1^ K)	3500–6600	600–6000	[14, 21]
Band gap (eV)	0.1–2.2 (direct)	0–0.08 (direct)	[23, 29, 30]
Carrier mobility (cm^2^ V^−1^ s^−1^)	20–10^4^		[7, 24]
Resistivity (Ω-cm)	10^− 6^	5 × 10^− 6^	[41, 42, 43]
Current density (A cm^−2^)	10^7^–10^8^	10^6^ -10^10^	[26, 27]
Cost (per gram in USD)	50–400	0.1–25	[44]

Due to their unique and desirable properties, CNTs have found many applications and incorporation into several commercial products to date.

**Fig. 3:**
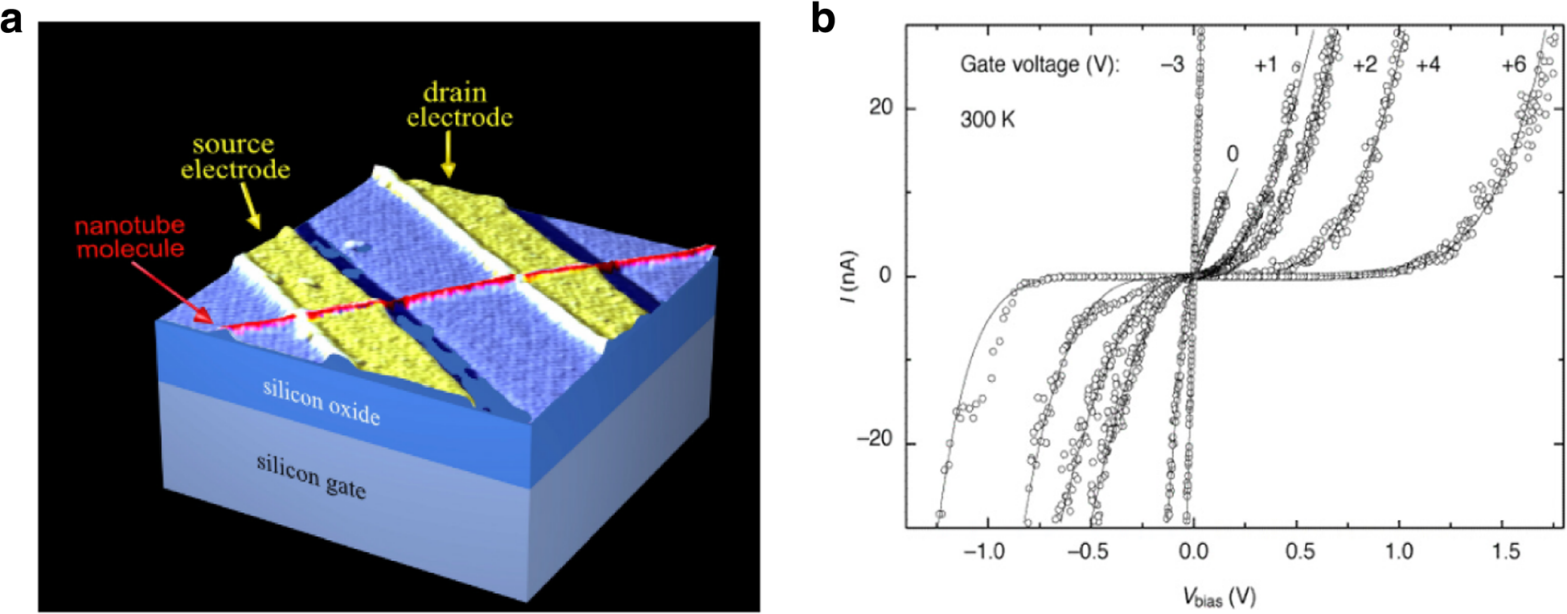
**a** Schematic illustration of the initial CNTFET demonstration. The transistor could be turned on by applying a gate voltage to the silicon substrate (back gate) that induces carriers into the nanotube channel bridging the source and drain electrodes. Adapted from [45]. **b** *I*-*V* characteristics of CNTFET showing switching between ohmic and nonlinear behaviors at different gate voltages. Adapted from [48]

Semiconducting CNTs have been used in field-effect transistors (FETs) [7, 45–48] (Fig. 3 shows the schematic and *I*-*V* characteristics of a CNT field-effect transistor (CNTFET) exhibiting switching for different gate voltages); metallic CNTs are used as interconnects [49, 50]; both single-walled and multi-walled nanotubes have also been used in various THz applications (described below) and Schottky diodes [51]. CNTs are currently being used in lithium ion batteries for efficient energy storage [52, 53]; hydrogen fuel cells [54] and CNT coatings have been extensively used to sense gases like ammonia, hydrogen and methane [55]. Super-aligned carbon nanotube films have been used in liquid crystal displays (LCDs) [56]; CNTs have also found applications in transparent conductive films [57].

CNT-material composites have been found to have enhanced properties. For example, CNT-reinforced epoxy composites were found to have a 24.8% increase in tensile strength compared to the pure epoxy matrix [58]. Additionally, by introducing a small amount of magnetically aligned CNTs into carbon fiber reinforced polymer composites, the flexural modulus and load-carrying capacity were increased by 46% and 33%, respectively [59]. Also, thermoplastic polyurethane (TPU)-based composites filled with CNTs and intumescent flame retardants were shown to achieve good flame retarding, prompt self-extinguishment, good electromagnetic interference shielding properties and increased electrical conductivity [60]. In addition, mixing CNT powders with polymers would increase stiffness, strength and toughness for load-bearing applications [61, 62]. MWCNTs—magneto-fluorescent carbon quantum dots, a carbon nanotube-composite, has been used as a carrier for targeted drug transport in cancer therapy [63]; nanofluids, containing dispersed CNTs, show enhanced heat transfer characteristics [64]; and nitrogen-doped CNTs (N-CNTs) can be used as adsorbents in food analysis, to trace bisphenols in fruit juices [65].

Researchers have begun using SWCNTs as building blocks of novel high-frequency devices [66]. In the presence of external magnetic fields and electric fields, certain nanotubes develop strong terahertz (THz) optical transitions, thus making them useful as tunable, optically active materials in THz devices. Several proposals for using CNTs in THz applications have been developed. They include a nanoklystron which utilizes efficient high-field electron emission from nanotubes, devices based on negative differential conductivity in large-diameter semiconducting nanotubes as well as single and multi-wall carbon nanotube antennae operating in the THz regime [66–68]. Due to their unique electronic properties, CNTs are being used as sources of terahertz (THz) radiation. Creating a compact, reliable source of THz radiation is very important for contemporary applied physics, as there are no miniaturized and low-cost THz sources currently available [66–68]. THz radiation lies between the microwave and infrared radiation in the electromagnetic spectrum. In this frequency range, electronic transport and optical phenomena merge with one another, and classical waves become quantum mechanical photons. This unique position of the THz range means they can only be studied by novel approaches which bridge the gap between the electronic and optical properties of materials, such as by using carbon-based nanomaterials. Researchers are also investigating the use of CNTs in fields including photovoltaics [69] and infrared (IR) detection [70].

The possibility of using CNTs as reactors for synthesis at the nanoscale is another area being explored [71]. Use of CNTs as catalyst supports for electrocatalytic oxygen reduction reactions (ORR) and oxygen evolution reactions (OER) is gaining widespread popularity [72–76]. In particular, various studies have shown the use of nitrogen-doped carbon nanomaterials for efficient ORR and OER reactions, where these CNT electrodes have demonstrated improved stability and electrocatalytic activity as compared to metals like platinum [76, 77].

**Fig. 4:**
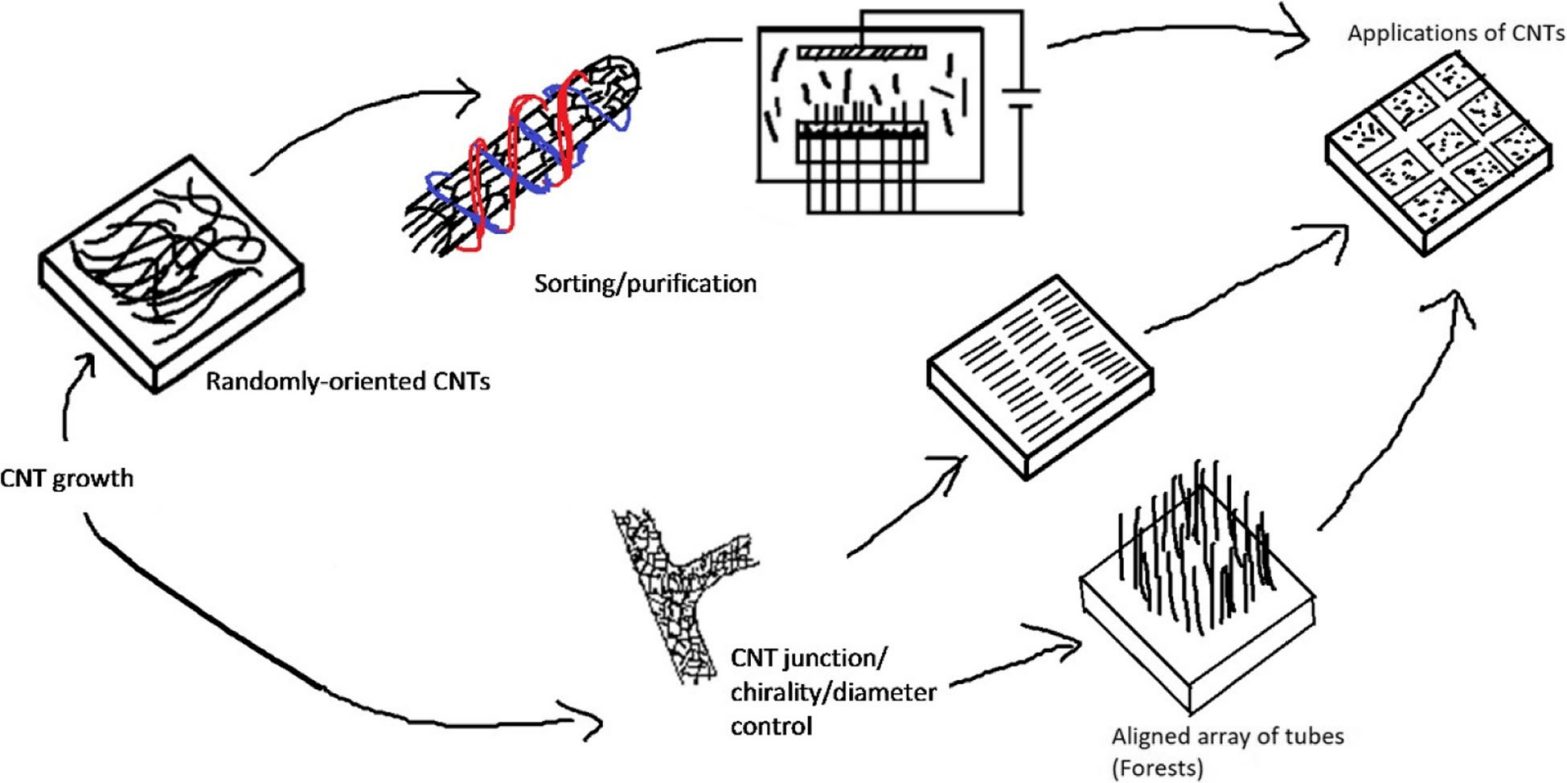
Schematic outline of the paper. In this review, progress on controlling the assembly and integration of CNTs from individual tubes (i.e. chirality, junctions and diameter) to various purification, assembly, alignment techniques and integration of large numbers of nanotubes for a wide range of applications is discussed

In general, precise control of the placement, type, orientation and/or structure of large numbers of CNTs is needed in order to optimize their performance for a given application such as those mentioned above. In this review, progress in carbon nanotube assembly and integration based on a variety of approaches will be reviewed and discussed. In particular, we first focus on techniques for controlling individual CNTs, both directly during growth, and via post-growth approaches. We then examine methods that have been developed for the integration of large numbers of nanotubes in parallel along with the resulting structures and ensembles. Lastly, despite tremendous progress over the past two decades in CNT fabrication and assembly, we highlight significant challenges that remain for both current and emerging applications using CNTs. A schematic outline of this paper is shown in Fig. 4.

## Controlling Individual CNTs

### CNT Growth—Overview

The most widely known techniques for fabrication of CNTs are arc-discharge, laser ablation and chemical vapor deposition. The carbon atoms that result in the formation of CNTs are liberated by methods utilizing current (in arc-discharge), high intensity laser (in laser ablation) and heat (in CVD). These techniques are discussed briefly in the following sections.

#### Arc-Discharge

**Fig. 5:**
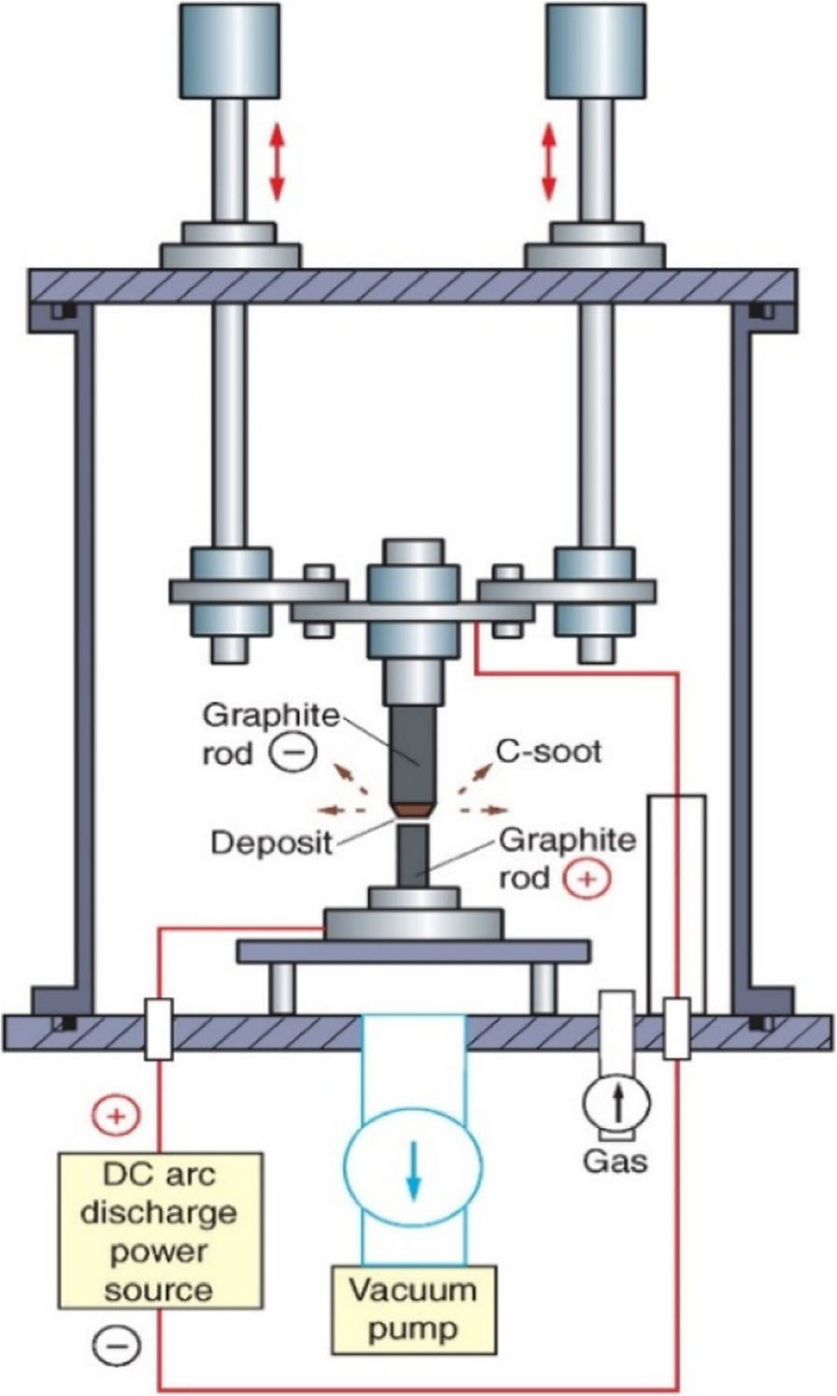
Schematic diagram of an arc-discharge system used to synthesize CNTs. In this technique, nanotubes are produced on one of the graphite electrodes when a large arcing current flows inside a metal reactor maintained at high pressure and temperature. Adapted from [79]

CNTs were produced from carbon soot of graphite electrodes using the arc-discharge method [78]. Arc-discharge method employs high temperature (over 1700 °C) for synthesizing CNTs. This method consists of two graphite electrodes, an anode and cathode (with diameters of 6 mm and 9 mm) which are placed approximately 1 mm apart in a large metal reactor as shown in Fig. 5 [79]. While maintaining an inert gas at a constant high pressure inside the metal reactor, a direct current of ~ 100 A is applied with a potential difference of ~ 18 V [80]. When the two electrodes are brought closer, a discharge occurs leading to the formation of plasma. A carbonaceous deposit which contains nanotubes is formed on the larger electrode. MWCNTs in the form of carbon soot of 1 nm to 3 nm inner diameter; and ~ 2 nm to 25 nm outer diameter were observed to be deposited in the negative electrode [1, 78]. By doping the anode with metal catalysts such as Cobalt (Co), Iron (Fe) or Nickel (Ni), and using pure graphite electrode as the cathode, SWCNTs could be grown up to a diameter of approximately 2 nm to 7 nm [81–83]. This technique can be used to grow large quantities of SW/MWCNTs. However, the major drawback of this technique is the limited yield quantity due to the use of metal catalysts that would introduce unwanted post-reaction products which need purification.

#### Laser Ablation

**Fig. 6:**
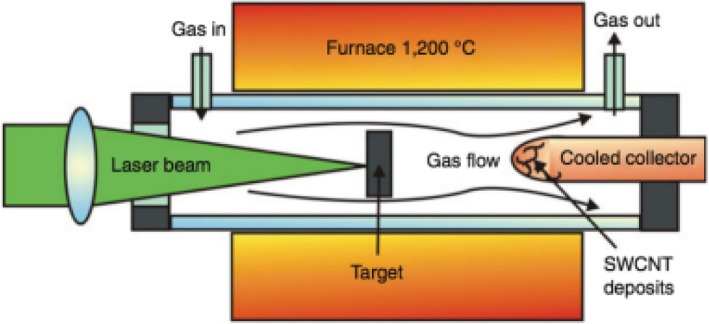
Schematic diagram of a laser ablation system used to synthesize CNTs. In this technique, CNTs are produced in a quartz tube furnace with the help of a laser beam that vaporizes a graphite target leading to self-assembly of CNTs on the surface of the reactor. Based on the type of electrodes (pure graphite or graphite doped with Co, Fe or Ni), the formed CNTs can be single or multi-walled. Adapted from [84]

This technique is similar to the arc-discharge technique; however, it employs a continuous laser beam, or a pulsed laser as shown in Fig. 6 [84] instead of arc-discharge. The laser beam vaporizes a large graphite target in the presence of an inert gas such as He, Ar, N_2_ etc. in a quartz tube furnace at ~ 1200 °C. Then, the vaporized carbon condenses and CNTs are self-assembled on the cooler surface of the reactor [85–88]. If both electrodes are made of pure graphite, MWCNTs are produced with an inner diameter of ~ 1 nm to 2 nm and an outer diameter of approximately 10 nm [89]. When the graphite target is doped with Co, Fe or Ni, the resultant deposit was observed to be rich in SWCNT ‘ropes’ or bundles (Fig. 1e). The yield and quality of CNTs produced depends on the growth environment such as laser properties, catalyst composition, growth temperature, choice of gases and pressure. This method can be expensive due to the need for high-power laser beams. One advantage of this technique is that post-growth purification is not as intensive as in arc-discharge method due to less impurities.

#### Chemical Vapor Deposition

Chemical vapor deposition (CVD) is commonly termed as catalytic chemical vapor deposition (c-CVD) due to the use of metal catalysts in the thermal decomposition of a hydrocarbon vapor. Catalysts play a very important role in the growth of CNTs. An ideal catalyst should be monodispersed on the surface of the substrate. It should also interact with the substrate appropriately via Van der Waals forces. Growth efficiency of the SWCNTs can be improved when there is a weak interaction between the catalyst and the substrate. At high temperatures, metal catalysts are very unstable and chirality-controlled growth of the SWCNTs becomes a challenging task. An ideal catalyst should offer good stability at higher temperatures and lead to controlled growth of CNTs with better diameter distribution. By increasing the interactions between catalyst support and the catalyst nanoparticles, control over some of the problems encountered at high temperatures may be achieved. Hydrocarbon sources may be in liquid (benzene and alcohol), vapor (carbon monoxide) or solid form (camphor) [79]. For the decomposition of hydrocarbons, nanometer-sized transition metal catalysts such as Fe, Co, Ni, Mo are commonly used [90–92]. In addition, metal catalysts like Cu, Au, Ag and Pt have also been used in some studies [93]. In some cases, these metal catalysts are mixed with catalyst supports such as SiO_2,_ MgO and Al_2_O_3_ in order to increase the surface area of the catalytic reaction involving the carbon feedstock and the metal particles [94].

The choice of the type of hydrocarbons and catalysts used determines the various growth mechanisms, termed as vapor-liquid-solid (VLS) or vapor-solid-solid (VSS) mechanisms. Of the two, the VLS mechanism is widely used. Here, the catalyst particles are in the liquid phase, where hydrocarbons are adsorbed on the metal particles and are catalytically decomposed. Next, the carbon forms a liquid eutectic by dissolving into the particle and later precipitates in to a tubular form upon super-saturation [95, 96]. On the other hand, VSS growth mechanism uses a solid catalyst [97].

**Fig. 7:**
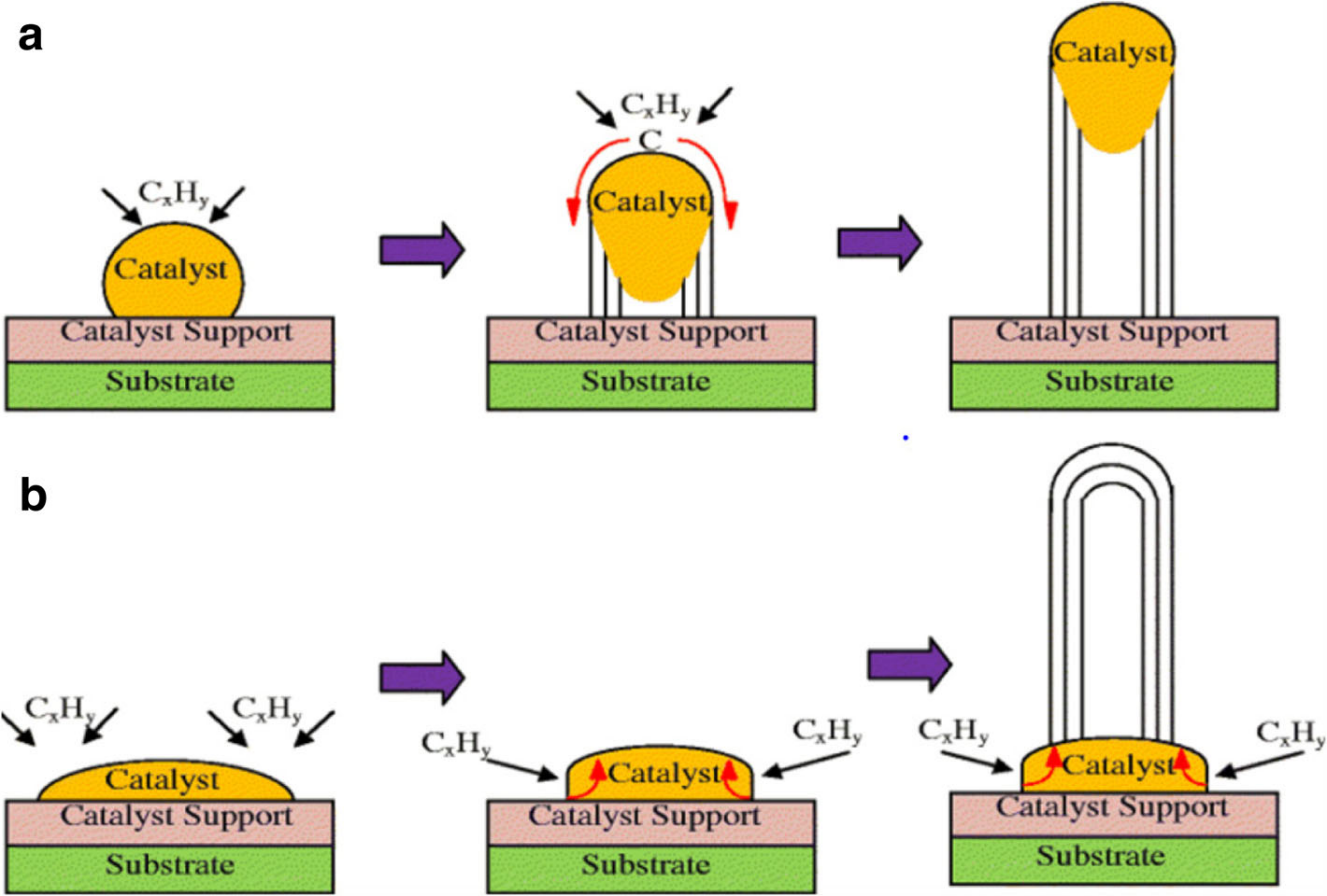
Different growth mechanisms of CNTs using CVD (Adapted from [101]). Based on the catalyst-substrate interactions, two types of CNT growth mechanics can be seen. **a**Tip-growth model: with weak catalyst-substrate interactions, a tip-growth is observed where hydrocarbon decomposes on top surface of the metal that causes carbon to diffuse downwards through the metal leading to CNTs growing out from the bottom of the metal. **b** Base-growth model: With strong catalyst-substrate interactions, a base-growth is observed where CNTs grow out of the metal far from the substrate while the catalyst is rooted at the base

**Fig. 8:**
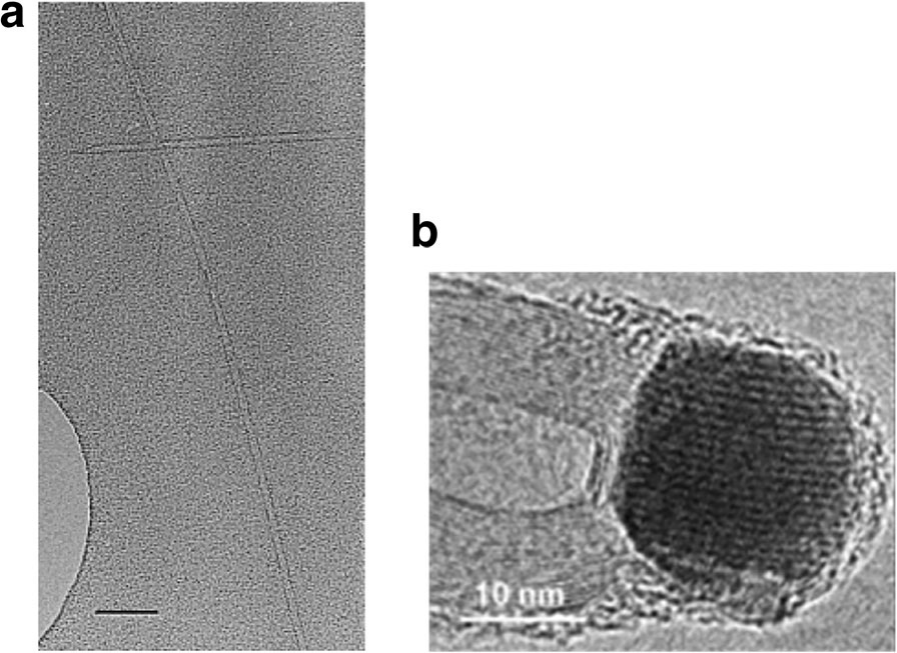
TEM images of CNTs grown via CVD (**a**) an isolated SWCNT grown using Fe_2_O_3_ catalyst with diameter of 5 nm. Scale bar equals 50 nm. Adapted from [102]. **b** MWCNTs grown with catalyst particle at tip end. Adapted from [103]

The synthesis method begins with decomposition of a hydrocarbon vapor in the presence of a metal catalyst at a temperature of ~ 600–1200 °C [98, 99]. When the hydrocarbon vapor interacts with the metal, it decomposes into carbon and hydrogen. Carbon gets dissolved into the metal while the hydrogen gas evaporates. Then, based on the catalyst-substrate interactions, growth of CNTs on the metal catalyst is either in the form of a tip-growth mechanism or a base-growth mechanism [100, 101] as shown in Fig. 7. Tip-growth mechanism is due to weak catalyst–substrate interaction. Here, the hydrocarbon decomposes on the top of metal while the carbon starts to diffuse through the metal. The CNT starts growing from the base of the metal and continues to grow longer till there is enough room for additional hydrocarbon decomposition based on the metal’s concentration gradient. In this process, the metal is pushed farther away from the substrate as shown in Fig. 7a. In case of a base-growth mechanism, there is a strong catalyst–substrate interaction. Similar to tip-growth mechanism, the hydrocarbon decomposes on the top of metal while the carbon starts to diffuse through the metal. However, due to strong catalyst-substrate interaction, the metal particle is not pushed higher, and the CNT grows on top of the metal as shown in Fig. 7b. Figure 8 shows examples of CNTs grown via CVD [102, 103].

Size and properties of the catalyst play a significant role in the growth of SWCNTs and MWCNTs using CVD. Smaller particle size (a few nm) leads to the growth of SWCNTs, whereas MWCNTs are formed when the particle size is larger (tens of nm) [1]. The type of hydrocarbons influences the shape of the CNTs produced. For example, methane, acetylene which are linear hydrocarbons, lead to formation of straight hollow CNTs. Cyclic hydrocarbons like benzene and fullerene produce curved CNTs [104].

In addition, the choice of substrate used also plays an important role in the growth of CNTs due to the catalyst–substrate interactions, in turn influencing the yield, quality and aspect ratio of the CNTs produced. Some of the commonly used substrates for growth of CNTs are made of materials like silicon [104], graphite [18], alumina [105, 106] and zeolite [107]. Studies have shown that use of zeolite substrates can result in high yields with narrow diameter distribution and that substrates made of alumina produce high yields of aligned CNTs with high aspect ratio [79, 108].

Along with the catalyst and substrate choices, structural control of individual CNTs is also affected by the temperature and the gas flow rate during the synthesis procedure. Control of gas flow rate during synthesis depends on the type of hydrocarbons used (i.e. gaseous, solid or liquid. An increase in the SW/MWCNT’s diameter is observed with an increase in the synthesis temperature [109]. For example, in case of a Fe–Co–zeolite system with camphor, the ideal temperature for SWCNT growth was reported to be around 900 °C, whereas for MWCNTs, the ideal growth temperature was reported to be 650 °C [100].

Of the three CNT manufacturing techniques discussed in this section, CVD is a widely used technique to manufacture CNTs due to its various advantages such as better controllability over CNT growth, low cost and use of low temperature [79, 105].

### Structural Control of Individual CNTs

#### Chirality Control

**Fig. 9:**
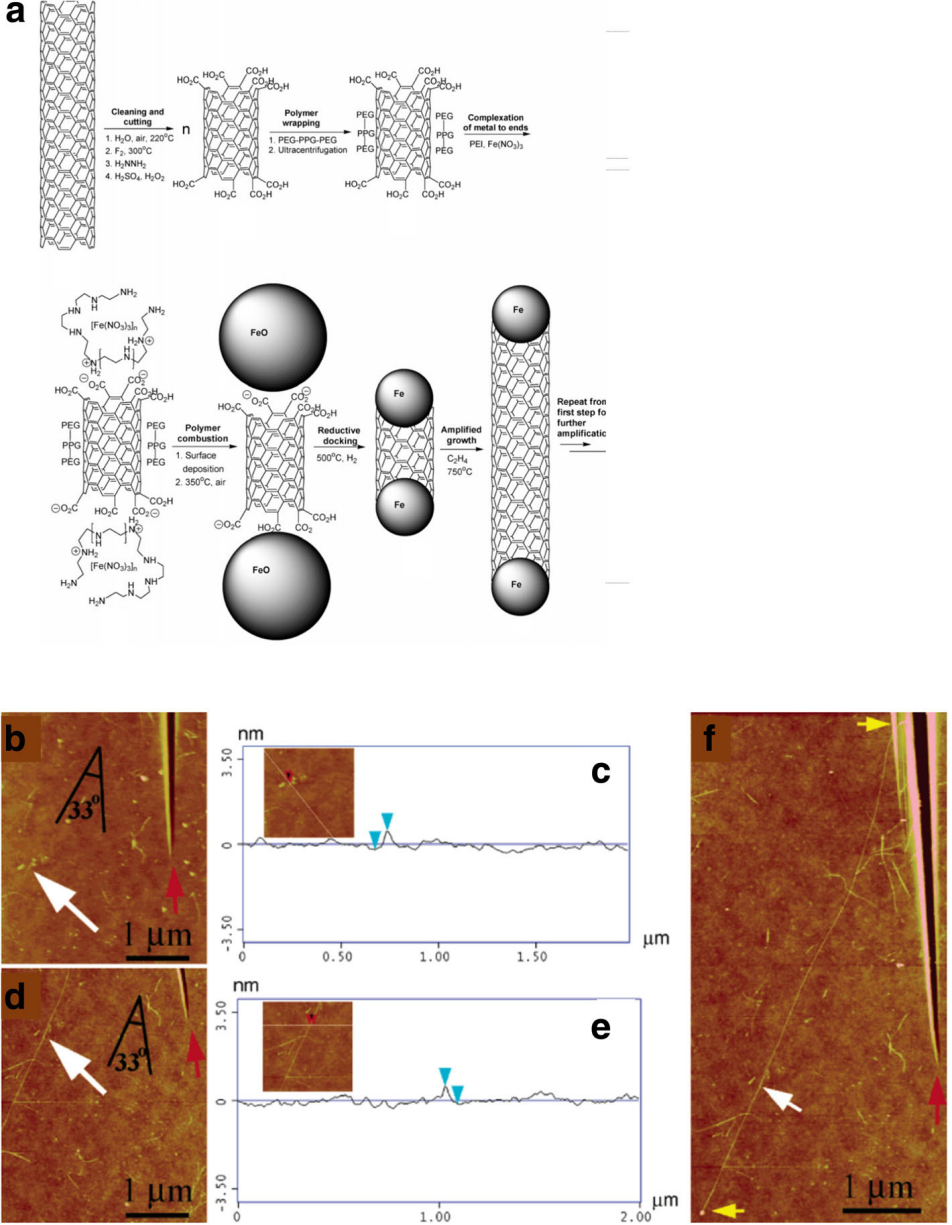
**a** Growth mechanism of SWCNTs using Fe seeds as growth templates. Adapted from [116]. **b**, **c** Atomic force microscope (AFM) images and height analysis of SWCNTs before the amplification process. Adapted from [116] and **d**, **f** after amplification growth process. Adapted from [116]. White arrows represent the original SWCNT seed location, red arrow indicates the seed position and angle relative to the locator inscription and yellow arrows show the entire length of the amplified nanotube. Adapted from [116]

Growing CNTs with controllable chirality is an important step in order to utilize them for various applications. This is because the chirality of a CNT determines various properties like electronic band structure and thus, the type of CNTs grown (i.e. metallic vs. semiconducting). Chirality control can be done by direct-controlled growth or post-synthesis separation approaches or by combining these methods [110] and is considered as one of the most challenging aspects in CNT growth [111]. Various parameters such as growth temperature, catalyst and hydrocarbon type influence the chirality of the CNTs. Direct controlled growth methods aim at controlling the chirality by controlling the nucleation process, as it is reported that during the nucleation process, chirality of a SWCNT is fixed [112]. For example, plasma-enhanced CVD (PECVD) has been used for the preferential growth of semiconducting SWCNTs [113]. In addition, semiconducting CNTs were also grown using ST-quartz substrates and methanol precursors [114]. Various growth parameters like type of catalyst, growth temperature and pressure and the source of the hydrocarbons play a significant role in influencing the nucleation which in turn controls the chirality of the tubes grown. Some of the techniques like use of CNT growth templates as seeds (both metal-based and non-metal based), growth initiated by carbon molecular-based precursors and use of nanoparticle-based catalysts have gained a great interest in this field. Some of these are discussed below:

In the first technique we describe, a single (*n*, *m*)-type SWCNT nanotube sample is cut into smaller pieces (seeds), each of which was aimed to be used as a template for the growth of a longer nanotube using a VLS amplification process (Fig. 9a). The main goal of this method was to grow large quantities of *n*, *m*-controlled structures. Each seed was polymer-wrapped SWCNT, end-carboxylated and tethered with Fe salts at its ends. During the growth process, Fe salts acted as growth catalysts and use of VLS mechanism aimed at achieving narrow diameter distribution [115]. SWCNTs grown with this method had a diameter similar to the diameter of the growth seed (Fig. 9b–f). However, details about the modifications in chirality of the tubes grown could not be clearly established [116]. In addition, this method also involves the need for complex purification steps due to the presence of metallic particles in the SWCNTs grown, thus affecting the final product’s quality.

**Fig. 10:**
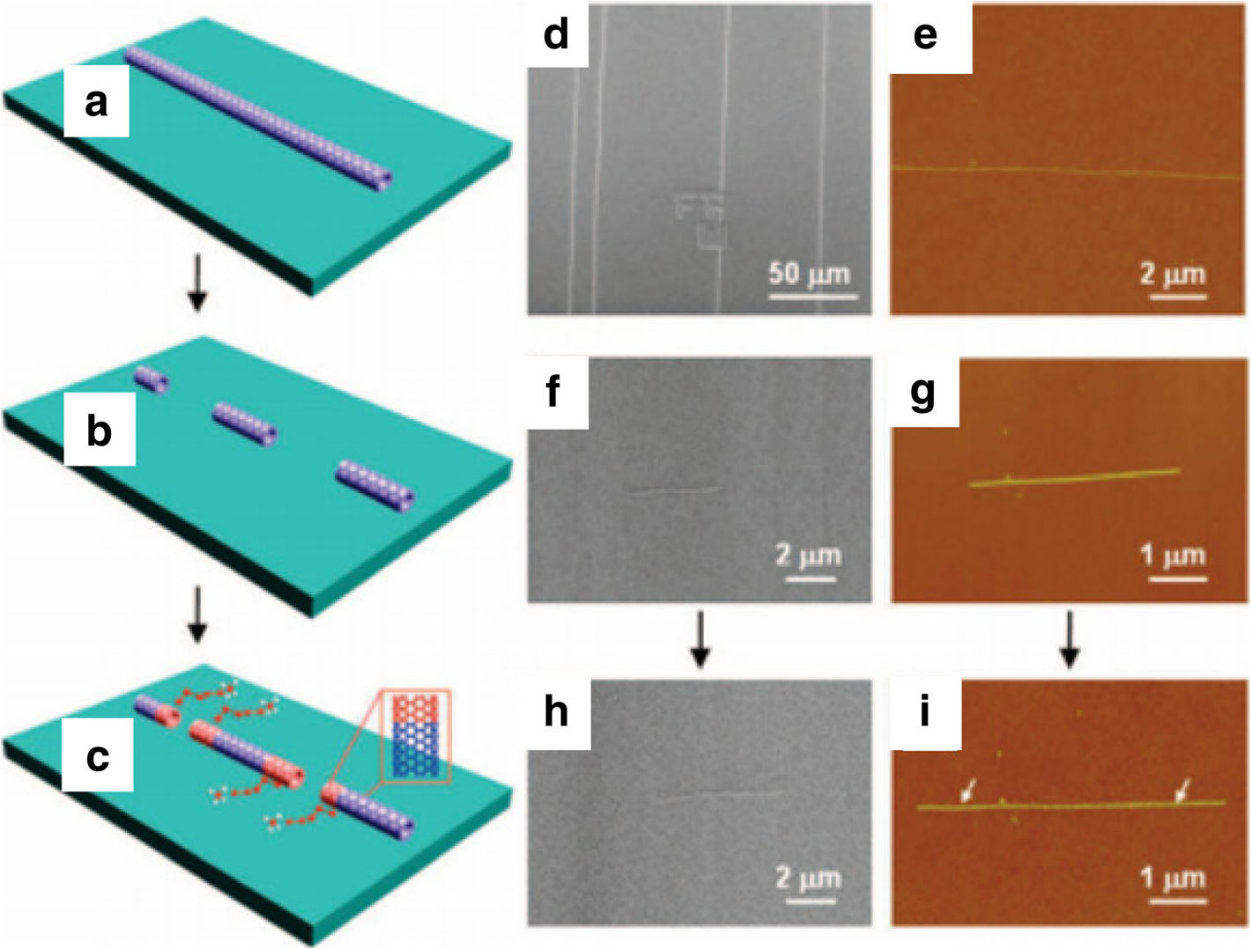
**a**–**c** Schematic diagram showing the growth process of ultra-long SWCNTs using e-beam lithography cut nanotube segments as the template via ‘cloning’ mechanism. Adapted from [118]. **d**, **e** SEM and AFM images SWCNTs used for preparing open-end SWCNTs seeds. Adapted from [118]. **f**, **g** SEM and AFM images of short parent SWCNTs segments for the second growth Adapted from [118]. **h**, **i** SEM and AFM images of duplicate SWCNTs continued grown from the SWCNTs. Adapted from [118]

As an alternative to metal-catalyst-based growth, another technique involved the controlled growth of CNTs by using semiconductor nanoparticles like Si and Ge as the growth templates. In one of these experiments, CNTs were grown using semiconducting nanoparticles (of size 5 nm or smaller), by introducing thermally decomposed carbon atoms from ethanol at 850 °C. However, CNTs grown in this experiment were considered to be of very low quality and low yield as compared to experiments using Fe, Co or Ni as catalysts [117]. Another growth technique was via an open-end growth mechanism, commonly referred as ‘cloning’ (Fig. 10) [118]. Here, the chirality of the SWCNTs was controlled by using open-end SWCNTs as seeds/catalysts without using a separate metal catalyst. Using these seeds, duplicate CNTs were grown on a SiO_2_/Si substrate. The total yield reported in this method was ~ 9%, which could be improved to 40% by growing SWCNTs using this method on a quartz substrate [118]. Another technique based on vapor-phase epitaxy was used to grow the SWCNTs with predefined chirality. This method combined CVD and SWCNT separation techniques by using deoxyribonucleic acid (DNA)-separated single chirality SWCNT seeds as the growth templates. These seeds were of very high purity (~ 90%) and C_2_H_5_OH and CH_4_ were used as the carbon sources. This experiment showed significant elongation of the SWCNTs grown from a few 100 nm to tens of microns. The total yield produced in this method was very low [110] and some of the studies related to vapor phase epitaxy (VPE)-based growth techniques are ongoing with aims to improve the yield.

**Fig. 11:**
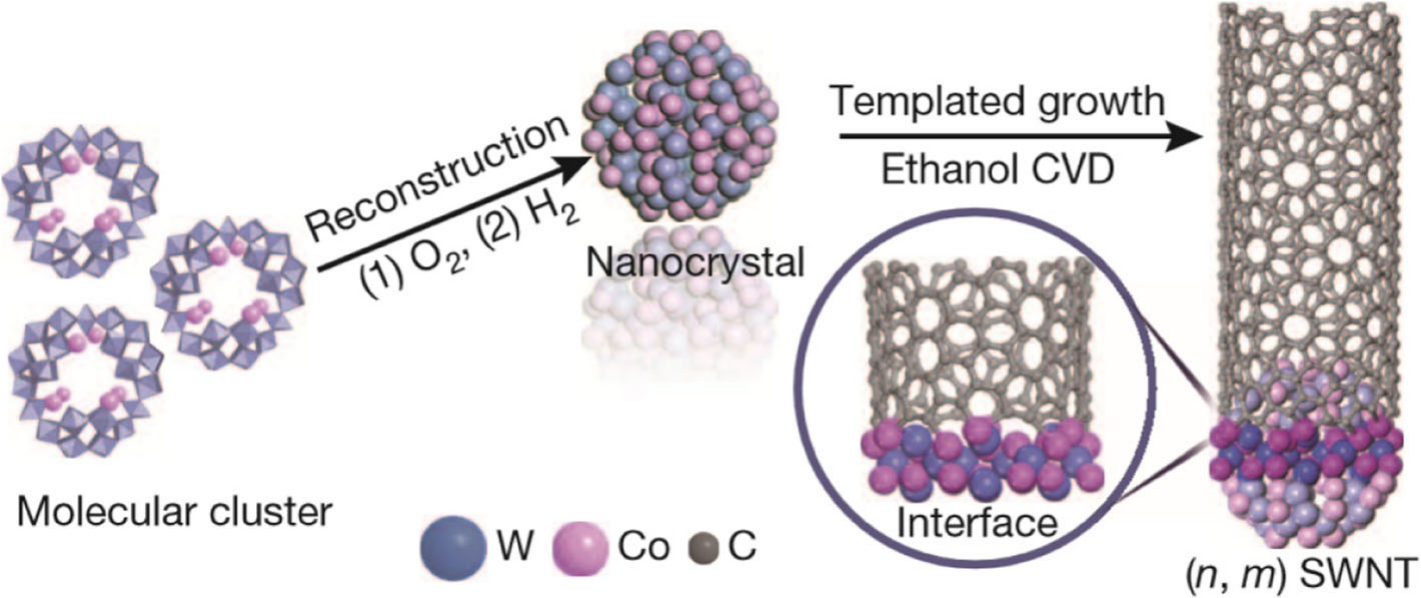
Growth of high purity, single chirality (12,6) SWCNTs using tungsten-based bimetallic solid alloy catalyst (W_6_Co_7_). These alloy nanoparticles catalyze the CNT growth on SiO_2_/Si substrates via ethanol CVD that help in chirality control during CNT growth. Adapted from [124]

One way to selectively grow chiral SWCNTs is by using silica substrate and Co-Mo catalyst [119]. Nanotubes of (6,5) and (7,5) chirality were obtained in this technique. With proper interaction between the Co and Mo oxides, aggregation of Co nanoparticles at high temperatures could be avoided. In addition, by optimizing the gaseous feed composition, growth and temperature, selectivity of (6,5) nanotubes was improved by ~ 55% [120]. Another approach for the selective growth of (6,5) SWCNTs was demonstrated using Co-Si catalyst and provided narrow distribution chiral SWCNTs [121]. High quality (6,5) tubes have also been grown at 800 °C using atmospheric pressure alcohol CVD on silica-bimetallic CoPt catalysts with narrow chirality distribution by tailoring the catalyst composition [122]. (9,8) SWCNTs were grown with high selectivity using Co nanoparticles and nanoporous Si support (TUD-1) [123]. Recently, (12,6) SWCNTs were synthesized using tungsten-based bimetallic solid alloy catalyst, W_6_Co_7_, with purity of > 92% (Fig. 11) [124]. This high level of purity was attributed to the W_6_Co_7_ catalyst which has a very high melting point of 2400 °C and provides a potential avenue for the growth of high purity SWCNTs by using nanoparticle catalysts.

Recently, selective growth of semiconducting SWCNT with diameters in the range of 0.8–1.2 nm was reported based on the deactivation process of the catalyst using a technique known as ‘catalyst conditioning process’ [125]. Here, the catalysts favoring the growth of metallic SWCNT are exposed to the catalyst conditioning parameters (oxidative, i.e. water) and reductive (i.e. H_2_) gases prior to the growth process which leads to the deactivation of these catalysts. An inverse relationship between yield and selectivity based on catalyst deactivation was reported in this work.

Evolving methodologies in the field of organic chemistry have enabled the synthesis of various carbon-based precursors that could be used in growing CNTs with controlled chirality. Some of the examples include flat CNT end-caps, three-dimensional CNT end-caps and carbon nanorings [111, 126, 127], which have all been tested and have proved to stimulate CNT growth under controlled environment. However, each of these approaches has some limitations [128].

**Fig. 12:**
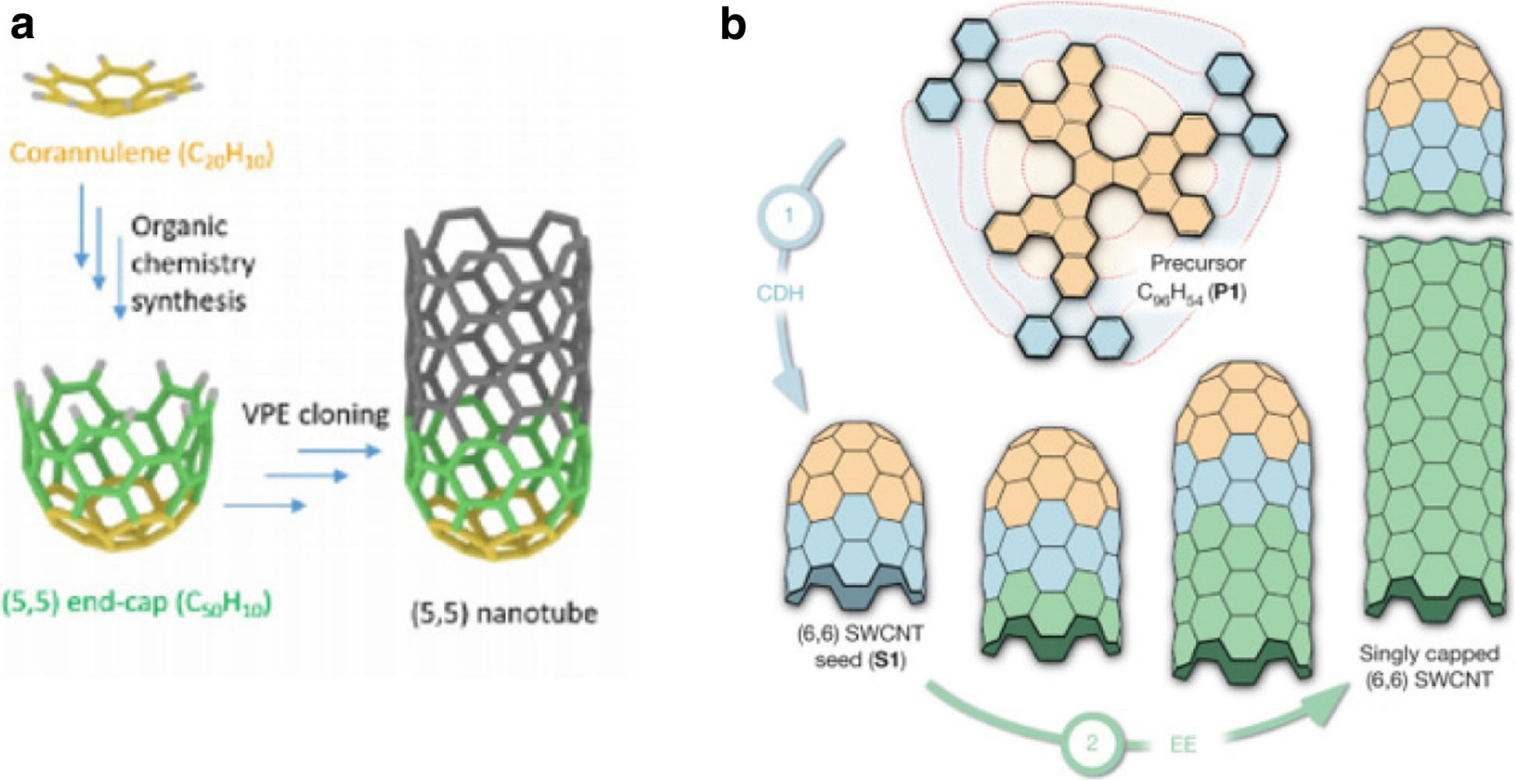
**a** Structure molecular end-caps used for chirality controlled synthesis of (5,5) SWCNTs through VPE elongation that was free of metals. Adapted from [130]. **b** Schematic illustration of a two-step bottom-up synthesis of SWCNTs from molecular end-cap precursors. Singly capped ultrashort (6,6) seeds lead to epitaxial elongation of nanotubes using the carbon atoms originating from the surface-catalysed decomposition of a carbon feedstock gas. Adapted from [110]

In one method, in order to yield hemispherical caps, thermal oxidation was used to open fullerndione. However, there were challenges in the synthesis of single chirality CNTs due to the lack of control in the formed hemispherical cap structures [129]. Synthesis of CNTs using carbon nanorings, viewed as sidewall segments without the cap was also developed [126] but the researchers were unable to control the chirality of the as-grown CNT. An alternative technique was developed by other researchers using an organic chemistry approach to synthesize pure molecular seeds of C_50_H_10_ as an end-cap of a (5,5) chirality nanotube [130]. In this method, the researchers demonstrated chirality-controlled synthesis of SWCNTs through VPE elongation that was free of metals (Fig. 12a). Even though the grown nanotubes were well aligned and of high density, in Raman characterization, it was observed that the synthesized SWCNTs were not (5,5) chirality. It was also observed that the as-grown semiconducting nanotubes were of smaller diameters [130]. Around the same time, another method was demonstrated to synthesize single chirality SWCNTs with predetermined chirality by using an end-cap precursor and planar single-crystal metal surface [131]. In this method, the researcher’s custom synthesized a precursor (C_96_, H_54_) using organic chemistry approach to yield (6,6) nanotube seed through surface-catalyzed cyclodehydrogenation process (Fig. 12b). Although, Raman characterization using 532 nm laser identified that the synthesized SWCNTs had (6,6) chirality, some researchers argue that 532 nm is not in resonance with (6,6) nanotubes. In their study, they quoted that 532 nm was in resonance with (9,2) or (10,0) chirality nanotubes. Furthermore, few others observed that the splitting of G band is not consistent with initial studies in this area that demonstrated the G band of armchair metallic nanotubes as a single symmetric peak [132, 133]. The researchers have recommended further Raman characterization, STM studies to determine whether the as-grown SWCNTs are of (6,6) chirality. The use of organic chemistry techniques has the potential to be referenced in further development of chirality controlled SWCNT synthesis due to the possibility of large-scale synthesis with higher purity.

Most of the fabrication methods used to grow SWCNTs produce polydisperse CNTs of metallic, semi-metallic and semiconducting properties. This variation is based on the way the graphene sheet is wrapped, denoted by the indices (*n, m*) that define the chirality of the tube grown. Steps to control these variations are essential for various applications of SWCNTs as the presence of multiple conductivity types can hinder the device performance. Some of the earlier techniques involved the use of gas-phase etchants like methane plasma [134], water vapor [135], oxygen [136, 129, 137] and hydrogen [134], that would etch metallic particles during the synthesis due to their higher reactivity with the metallic nanotubes, thereby leaving the semiconducting nanotubes behind.

Using floating catalyst chemical vapor deposition (FCCVD) technique with oxygen as an etchant in selective removal of m-SWCNTs, ~ 90% yield containing s-SWCNTs with diameters 1.4–1.8 nm were obtained [137]. However, oxygen can combine with other carbon-based materials due to its strong oxidizing properties during the growth process. Controlling the concentration of oxygen during the growth process is a challenging task. As an alternative, water vapor can be used as an etchant in the CVD technique, as it has a much weaker oxidizing ability. A yield of ~ 97% was reported with this technique [138].

**Fig. 13:**
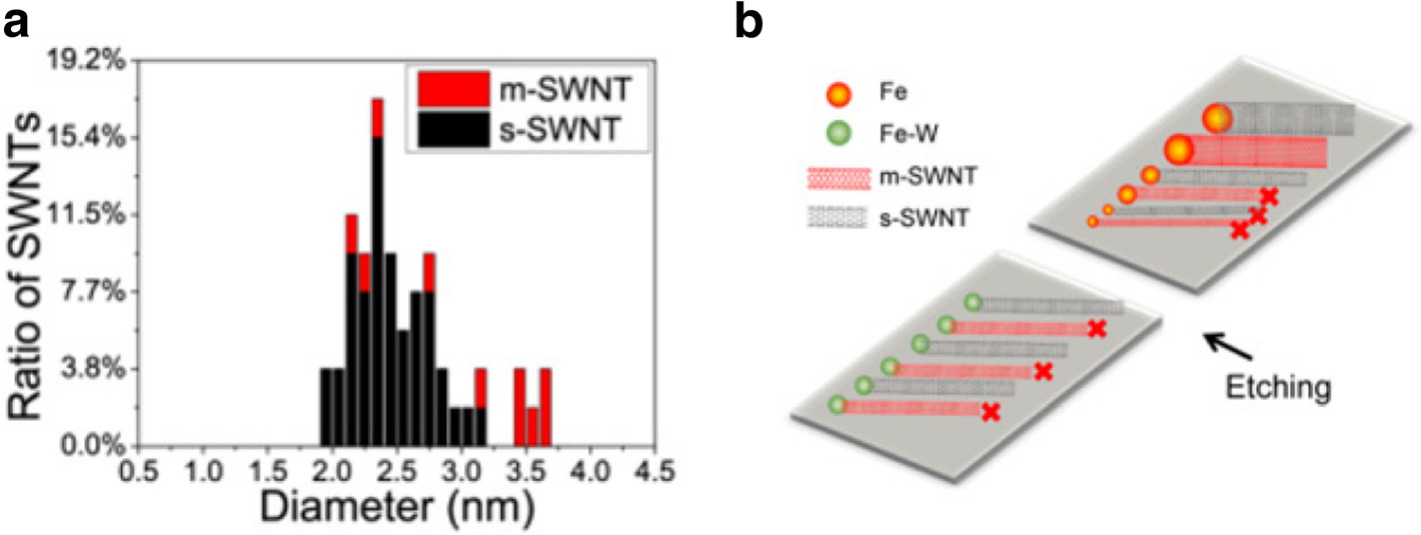
**a** Diameter and chirality distributions of the FeW-catalysed SWCNTs under a water vapor concentration of 522 ppm. About 90% of the as-prepared SWCNTs were reported to be in the diameter range of 2.0–3.2 nm adapted from [140]. **b** Schematic illustration of the diameter-dependent and electronic-type-dependent etching mechanisms during growth. High selectivity of s-SWNTs could be obtained by controlling the diameter via the Fe-W catalysts. Adapted from [140]

Recent studies have reported the importance of diameter dependence on the etching mechanisms. In one of the studies, m-SWCNTs were selectively etched using methane plasma, followed by annealing. At the end, s-SWCNTs are retained on the growth substrates which were stable at high temperatures [139]. By narrowing the diameter distribution to an optimal range of SWCNT diameter, most of the m-SWCNTs are etched within this range. In another technique, to control the diameter distribution, bimetallic solid alloy catalysts like Fe–W (Iron-tungsten) nanoclusters were used as catalyst precursors due to high-temperature stability of tungsten, which causes the nanoclusters to be stable during the CVD synthesis. Water vapor was used as an etchant during the growth process. A yield of ~ 95% was reported with this technique and the diameter of about 90% of the s-SWCNTs formed on the quartz substrate was reported to be in the range of 2–3.4 nm as shown in Fig. 13 [140]. A similar experiment using Fe nanoparticles as catalysts was performed where the overall yields showed broad distribution of the catalyst particle size due to mobility of Fe nanoparticles, which are usually in liquid state during high-temperature CVD growth [94].

**Fig. 14:**
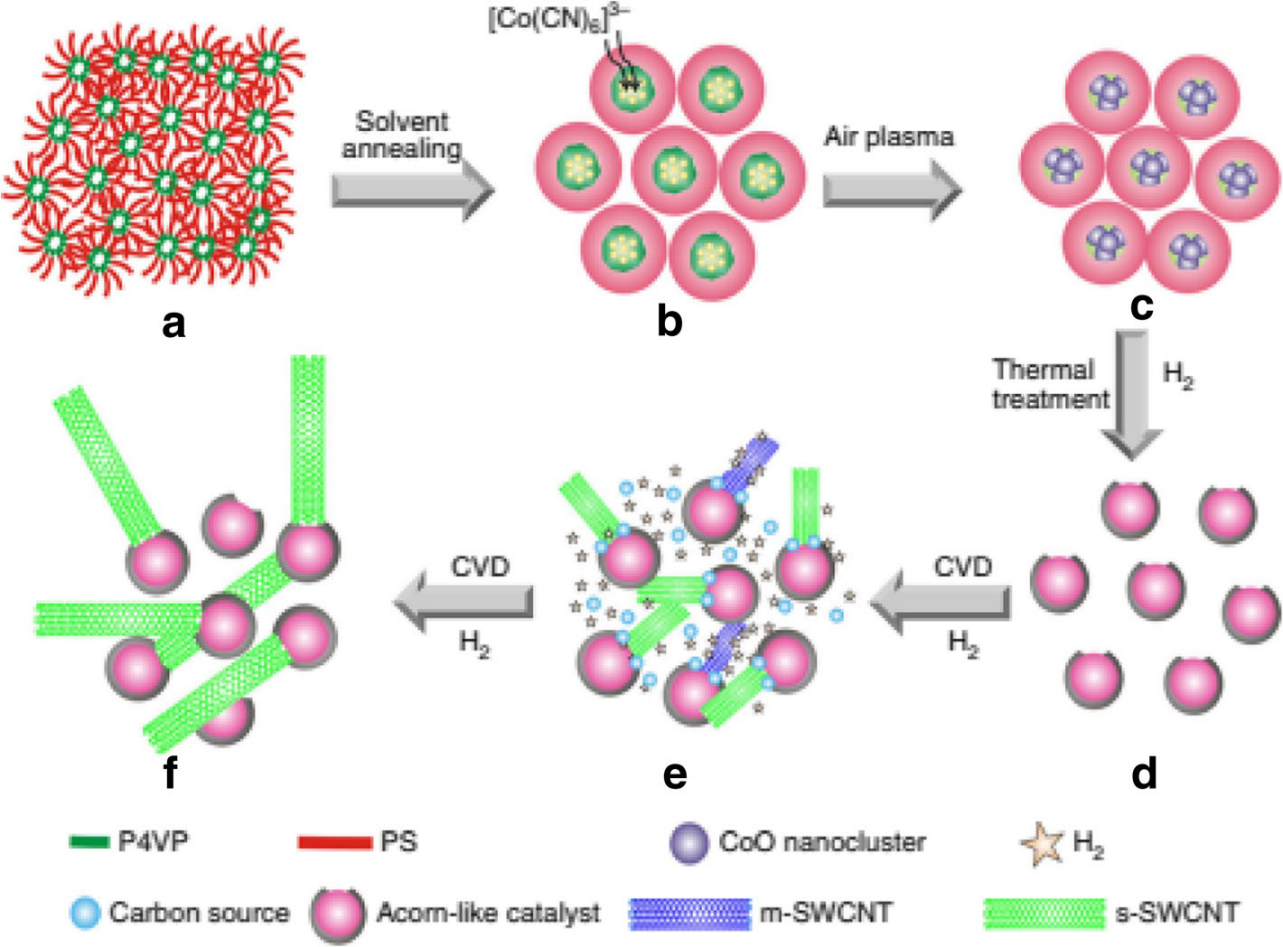
Step-by-step description of growth of s-SWCNTs with narrow diameter distribution using carbon-coated Co nanoparticle catalysts. Solvent annealing, use of air plasma followed by thermal treatment produced a yield of ~ 95% s-SWCNTs with diameters of about 1.7 nm. **a** Poly-(styrene-block-4-vinylpyridine) film self-assembled into vertical nanocylinders. **b** Formation of phase-separated nanodomains from the vertical nanocylinders and adsorption of K3[Co(CN)6]3 catalysts onto them. **c** CoO nanoclusters partially surrounded by a polymer layer. **d** Co catalyst nanoparticles partially coated with carbon to produce acorn-like bicomponent catalysts. **e** Growth of SWCNTs with a narrow diameter distribution from the partially carbon-coated Co nanoparticles followed by in situ etching of m-SWCNTs. **f** s-SWCNTs with a narrow band-gap distribution. Adapted from [138]

Another technique to grow s-SWCNTs with narrow diameter distribution is using carbon-coated cobalt nanoparticle catalyst (termed as acorn-like catalyst) as shown in Fig. 14. The Co nanoparticle acts as active catalytic phase for SWCNT growth. Carbon coating on the outer end prevents aggregation of Co nanoparticles, a major problem faced by most growth methods that lead to formation of larger particles during SWCNT growth at high temperatures [141]. In this technique, the yield of s-SWCNTs grown was ~ 95% with a very narrow diameter distribution centered at 1.7 nm [138].

#### Controlling CNT Geometry

##### Diameter

**Fig. 15:**
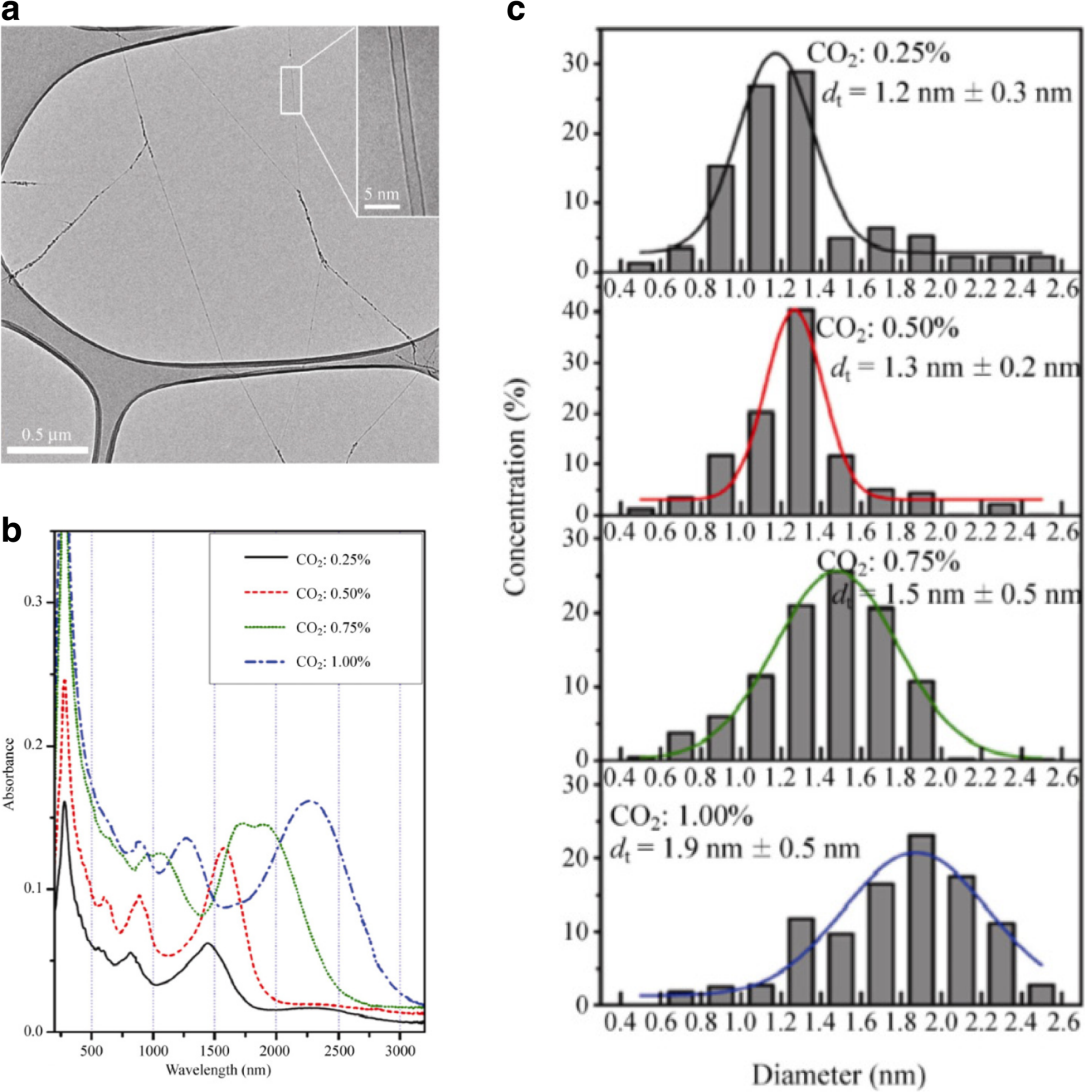
**a** TEM image of SWCNTs grown by adding CO_2_ along with carbon source. (Inset) shows the TEM image of an individual SWCNT Adapted from [142]. **b** Plot showing the absorption vs wavelength of SWCNTs grown with different CO_2_ concentrations. Adapted from [142]. **c** The corresponding diameter distributions of SWCNT samples with different CO_2_ concentrations. Adapted from [142]

Growth of SWCNTs with controllable diameters is regarded as one of the critical parameters in influencing its electrical, surface functionalization and thermal properties [1]. Properties such as band gap and chirality can be controlled by variations in the diameter of the SWCNTs formed. SWCNTs diameter control may be via their growth using floating catalyst method or from a substrate growth method with catalysts deposited on top or using template growth approach. Of the first two techniques, growth via floating catalyst method offers better control over the diameters of the tubes grown due to limited aggregation as catalysts are not restricted on a single plane of the substrate. Studies have shown diameter control in the range of ~ 1.2 to 2.1 nm using this method [126]. In one of the studies, diameter control was achieved by adding CO_2_ (which acts as an etching agent to etch tubes with small diameters) with the carbon source into the aerosol CVD reactor. The corresponding transmission electron microscope (TEM) image and the absorption vs. wavelength plot of SWCNTs grown with different CO_2_ concentrations is shown in Fig. 15 below. Increasing the concentration of CO_2_ leads to the shift in SWCNT diameters from 1.2 to 1.9 nm [142] as shown in Fig. 15c. Size and properties of the catalyst also play a significant role in the controlling the growth of SWCNTs and MWCNTs. Smaller particle size (a few nm) leads to the growth of SWCNTs, whereas MWCNTs are formed when the particle size is larger (tens of nm) [143]. For example, with Fe catalyst of average diameters of 9 and 13 nm, MWCNTs of average diameter 7 and 12 nm were produced [105].

Substrate growth method aims at minimizing particle aggregation by increasing catalyst spacing. For example, centrifuging the nanoparticles before deposition via CVD using ferritin catalyst particles leads to a diameter control in the range of. 1.9 to 2.4 nm [144]. Alternatively, by sandwiching Fe between Al_2_O_3_ in a sandwiched catalyst model, SWCNTs with diameters between 0.8 to 1.4 nm were synthesized [145]. However, SWCNTs grown using these techniques were entangled due to large catalyst spacing.

**Fig. 16:**
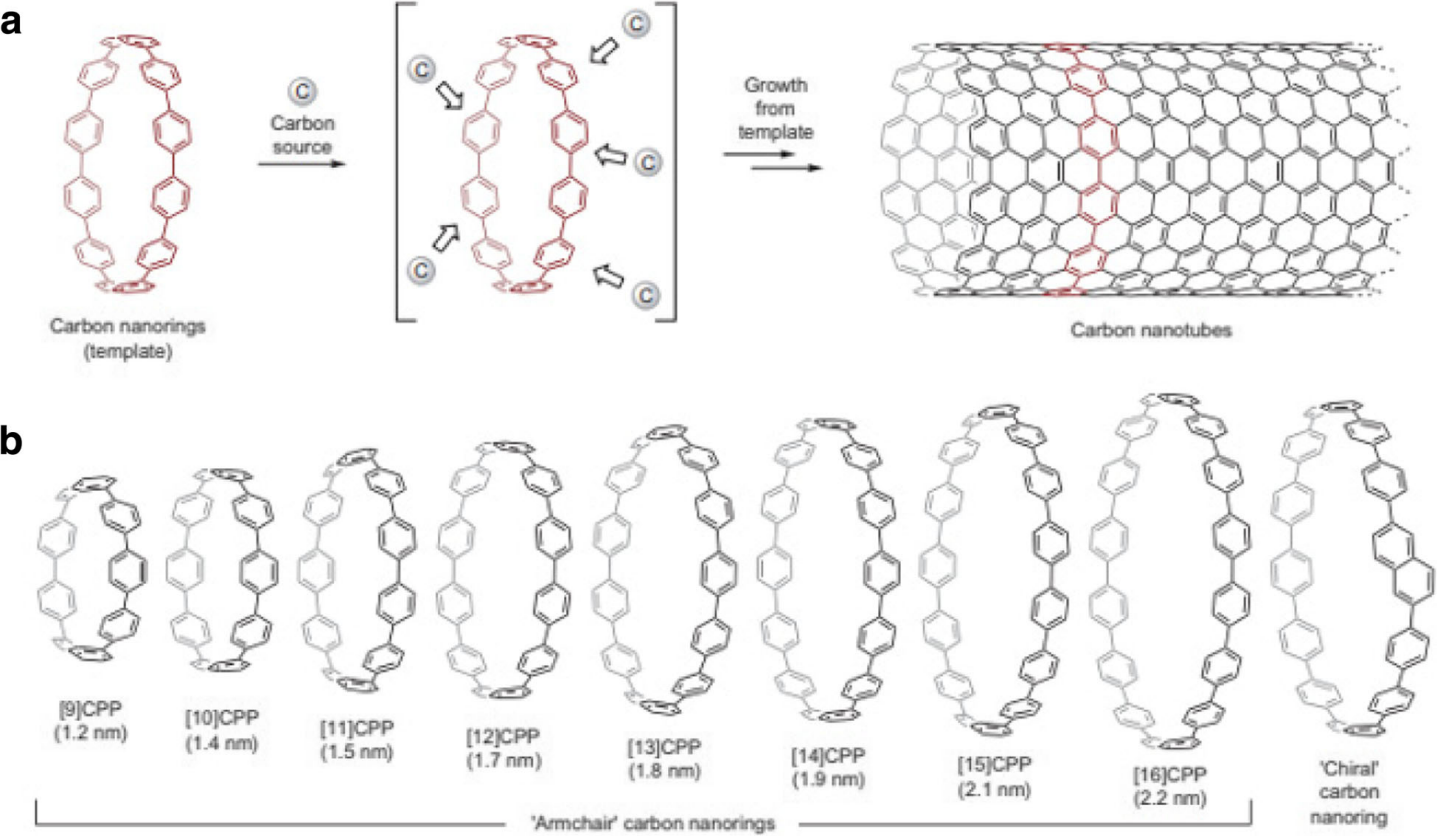
**a** Schematic of template-based CNT growth using carbon nanorings (cycloparaphenylene) that represent the shortest sidewall segment of armchair CNTs. Adapted from [126]. **b** Representation of various carbon nanorings grown using the template-based method and their corresponding diameters in nm. Adapted from [126]

Another way of controlling the diameters of SWCNTs is by using a template-based growth approach [126, 146–148]. Use of carbon nanorings (cycloparaphenylenes), representing the shortest sidewall segment of armchair CNTs (Fig. 16) as growth templates and ethanol as a hydrocarbon source, SWCNTs with diameters in the range of 1.2–2.2 nm were grown. Different types of nanorings (based on number of benzene rings in the structure) were used as growth templates. The diameters of SWCNTs grown were similar to the diameter of the carbon nanorings used, thereby providing an avenue for diameter control of SWCNTs using organic chemistry approaches.

**Fig. 17:**
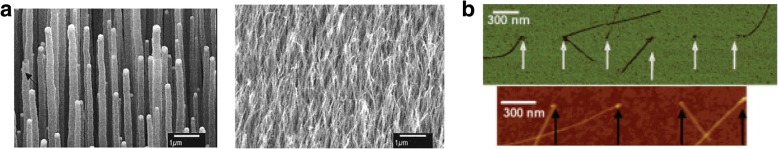
CVD based growth of CNTs produced using different diameter nanoparticle catalyst. **a** SEM images of CNTs produced with different diameters (250 nm and 20 nm in diameter) using nickel-coated glass substrates. Adapted from [149]. **b** AFM images of nanotubes grown using lithographically patterned catalyst and Co nanoparticles with a diameter of ∼ 1.7 nm. Adapted from [150]

Several methods report the growth of MWCNTs with controlled diameters [149–154]. In one of the methods, aligned CNTs with diameters in the range of 20–400 nm and lengths between 0.1 and 50 μm were produced using the plasma-enhanced hot filament CVD method by tuning the catalyst size (Fig. 17a). Another method reported the importance of supply of carbon reactant and the growth temperature in the formation of large diameter nanotubes [105]. Here, the use of an iron nanocluster with diameter of 9 nm, ethylene as the carbon reactant and growth temperature of 900 °C, large diameter nanotubes with two or three walls were produced. Alternatively, arrays of SWCNTs with diameters of ~ 1.5 nm were obtained using lithographically patterned metallic nanoclusters (Fig. 17b).

##### Junctions

**Fig. 18:**
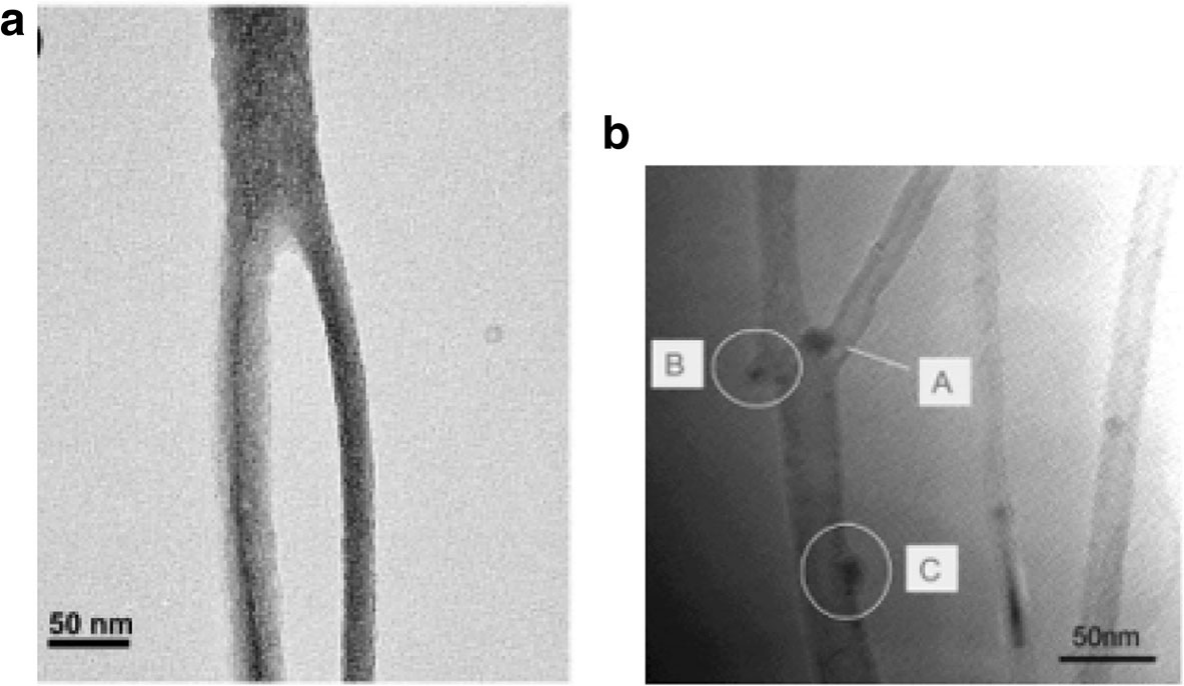
**a** TEM image of a MWCNT Y-junction nanotube grown by CVD using branched nanochannel anodic alumina templates. The grown Y-junctions were reported to be 6 to 10 μm in length with tunable diameters. Adapted from [159]. **b** TEM image of MWCNT Y-junction nanotube grown using Ti-doped Fe catalysts. Catalyst present at the junction (shown as A) leads to the formation of the two branches. B shows a Y-junction grown from catalyst particles that attach on the walls of the nanotube. C represents a catalyst nanoparticle that does not lead to further branching. Adapted from [161]

Modifications in the growth of CNTs leading to junction-like formations can create nanotube structures like the three-terminal Y-junction that could be used for novel electronic switching devices and transistors [155–158]. Y-junction nanotubes can be grown by CVD using anodic alumina templates with adjustable stem and branch templates [159, 160] as shown in Fig. 18a. Another method used Ti-doped Fe catalysts in the growth process to produce MWCNTs (~ 90%) branched in the form of a Y-shaped junction on quartz substrates (Fig. 18b) [161].

**Fig. 19:**
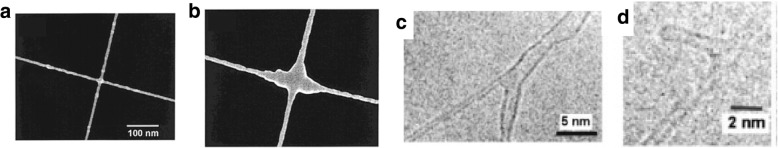
Growth of a MWCNT nanotube junction (**a**) before and (**b**) after soldering by deposition of amorphous carbon via electron beam irradiation. Adapted from [163]. **c** Y-shaped junction formed by electron beam irradiation. Adapted from [162]. **d** T-shaped nanotube junction formed after irradiating a preformed Y junction. Adapted from [162]

In addition to the above techniques, SWCNT junctions formed via crossing of different CNTs connected via irradiating the junction with electron beam, using scanning electron microscopy (SEM) have also been reported [162, 163]. Here, under the influence of electron beam, hydrocarbons used in the growth process are transformed into amorphous carbon which is then utilized to attach the nanotubes and form mechanical junctions (Fig. 19a, b). In another similar work, various carbon nanotube junctions (Y-, T-shaped) were formed by electron beam welding which induced structural defects in the nanotubes, leading to the joining of tubes by cross-linking of dangling bonds (Fig. 19c, d) [162].

**Fig. 20:**
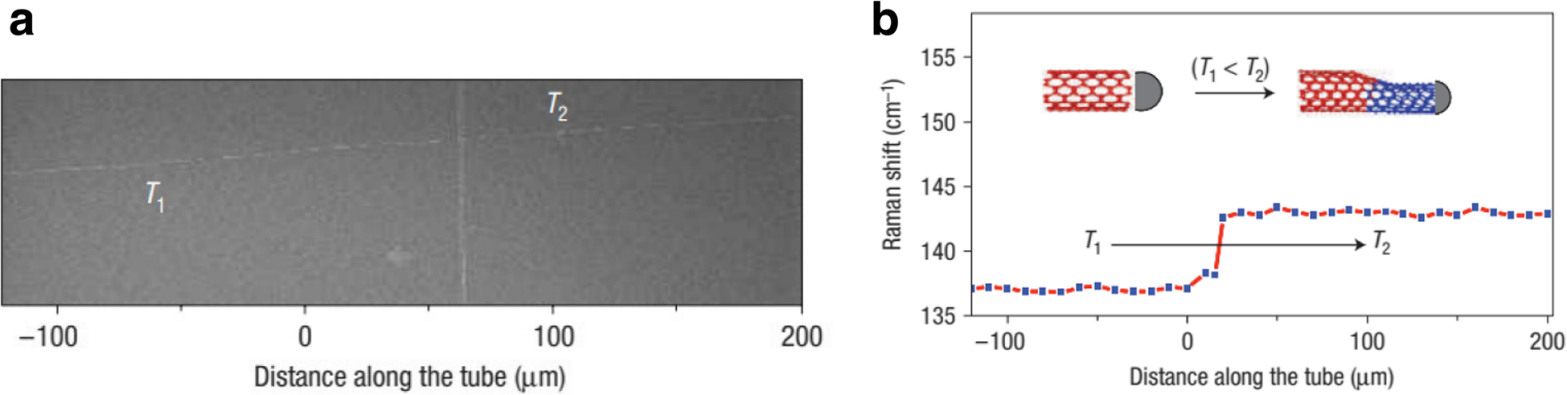
**a** SEM image of a two-terminal SWCNT intramolecular junction formed by varying the temperature during CVD growth from 950 to 900 °C (temperatures are indicated by T_1_ and T_2_). Adapted from [141]. **b** The corresponding shift in the Raman spectra with variations in the temperature. Inset shows the schematic illustrations of SWNT diameter variations with temperature. Adapted from [141]

Alternatively, two-terminal SWCNT junctions can be grown in a controlled manner using temperature modulation during the CVD process (Fig. 20) [141]. In this method, by altering the growth temperature, systematic variations in the diameter and chirality of the SWCNTs lead to the formation of SWCNT intramolecular junctions. These junctions were grown at desired locations by increasing the temperature of the substrate locally using infrared light during CVD. It was also observed that increasing the temperature led to a decrease in the diameter of the growing junctions and vice versa, with no change in the catalyst particle present at the growing tip [141].

### Post-Growth Purification/Sorting of Single Tubes

Understanding CNT sorting methodologies is important as many of the advanced applications, such as FETs and nanoscale sensors, require monodispersed samples with little structural variation [164]. Before CNT sorting can take place, the tubes must be dispersed in a liquid medium (water or organic solvents). Unfortunately, there are certain constraints which may prevent separation in an aqueous dispersion. For example, CNTs have very strong Van der Waals interactions which restrict sorting [87]. There are several well-developed techniques currently being used for the post-growth purification or sorting of tubes. Some of these are discussed below.

**Fig. 21:**
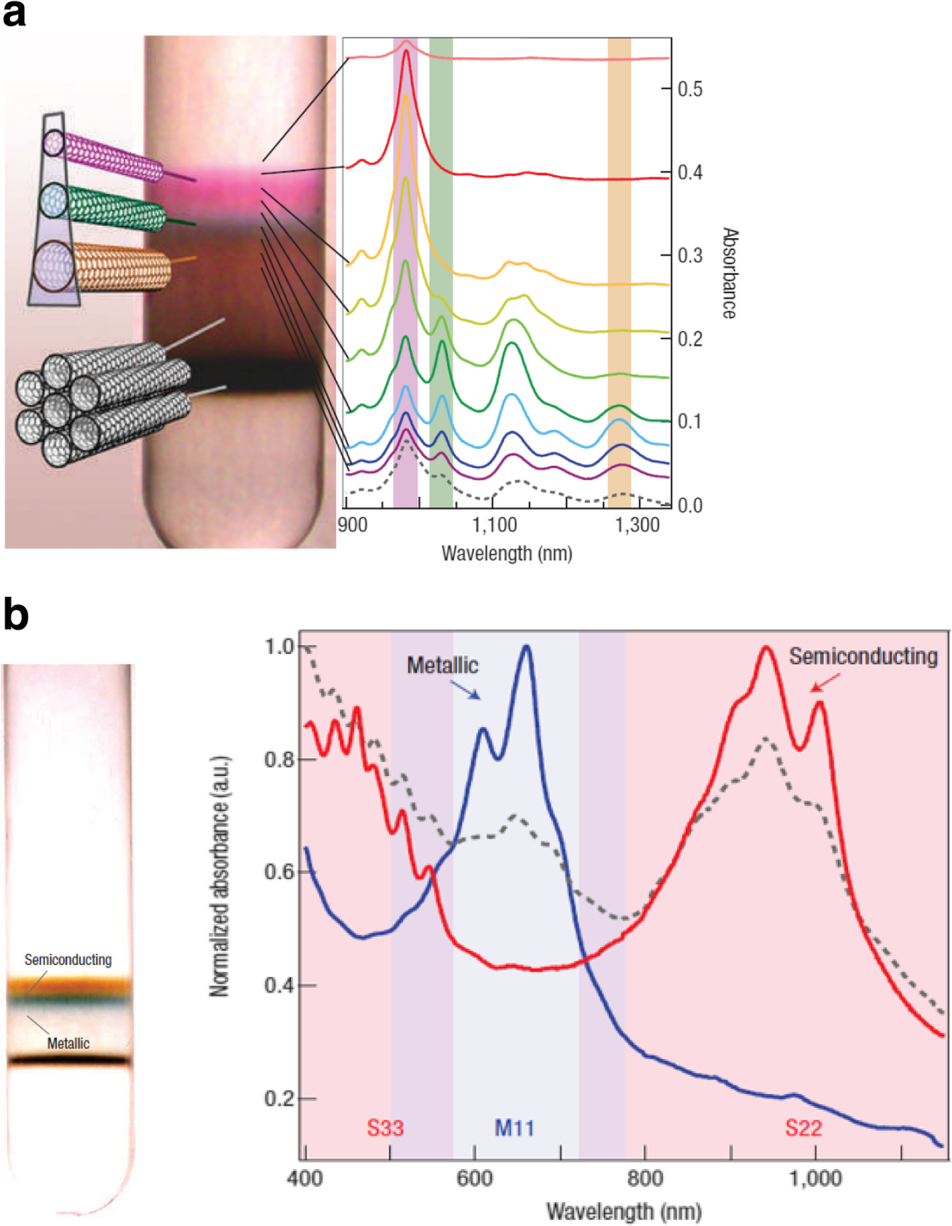
**a** Illustration of DGU separation of tubes coated with surfactant based on their diameter and metallicity. The near infrared absorption spectra of SWCNTs is also shown. Adapted from [166]. **b** Clear separation of SWCNTs by electronic type and the corresponding absorbance spectra for semiconducting SWCNTs (in red) and metallic SWCNTs (in blue) is shown. Adapted from [166]

One of the techniques, commonly referred to as the density gradient ultracentrifugation (DGU), has been shown to produce a high yield of pure SWCNTs, without much need for chemical treatment of the sample [165, 166]. DGU, which depends entirely on the buoyant density of the CNT, is an isopycnic separation process. The process is achieved by wrapping the SWCNT sample with a surfactant (Fig. 21) [166]. After the grown SWCNTs are mixed with the surfactant, the aqueous dispersion of surfactant-encapsulated tubes is added to the centrifuge tubes, which contains a pre-existing density gradient medium. A strong centrifugal force is then applied, and it causes the surfactant-wrapped SWCNTs to be separated by the movement of SWCNTs to regions of the density gradient medium which match the tubes’ buoyant densities (isopycnic points). The aqueous dispersions of the SWCNTs are produced by using either linear chain surfactants or bile salts. The density gradient medium is usually made of a salt (lithium chloride, cesium chloride, sodium chloride) solution in water. Nonlinear gradients are preferred because they are very sensitive and allow trapping of particles over the entire length of the centrifugal cell. The gradient density and its variation are important to the sorting process wherein, the gradient needs to be set up such that the distance between the tubes and their isopycnic points is minimal. As the density gradient medium responds to the centrifugal force, it leads to steeper gradient over time and hence redistribution of the density profile takes place during centrifugation [167]. After the centrifugation process, the sorted SWCNTS are removed layer by layer using the fractionation process (using piston, upward and downward fractionation methods), which involves extracting quantities of mixtures to different aliquots which vary in composition with respect to the density gradient of the original mixture. Uniform surfactant coverage is important or adsorbed surfactant molecules will begin to aggregate and form clusters along the tube sidewalls, thereby impeding effecting separation of the tubes. To separate metallic and semiconducting tubes, a co-surfactants mixture is used for the ultracentrifugation process. After the semiconducting tubes have been separated, chirality enrichment of tubes is carried out to generate samples that are rich in a certain chirality of tubes, and the resulting semiconducting-SWCNTs-enriched fraction is passed through a dialysis membrane to remove the surfactants from the SWCNT sidewalls [168]. Finally, the tubes are characterized using various optical spectroscopy methods.

**Fig. 22:**
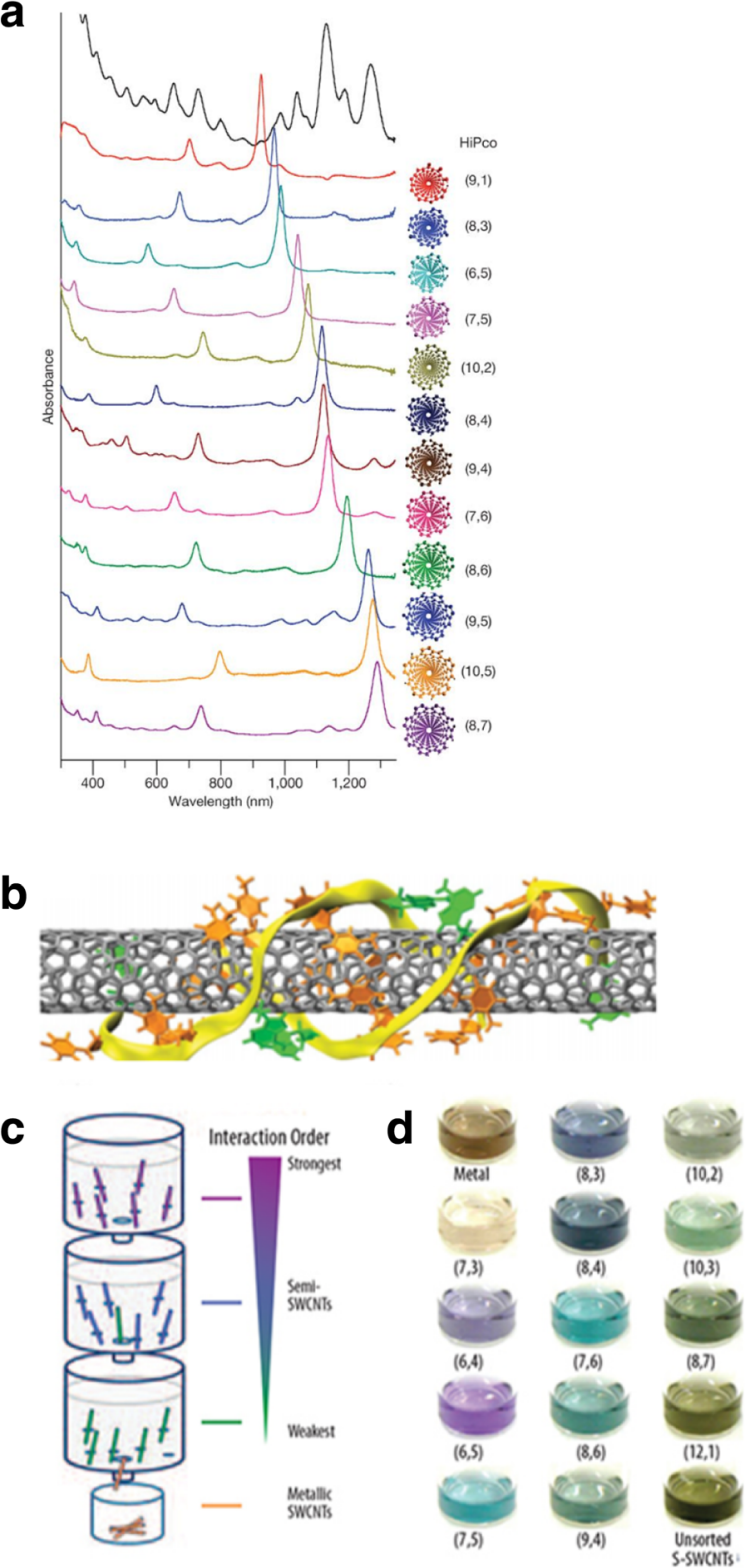
Purification of CNTs with defined helicity with the aid of specific DNA sequences using IEX. **a** Absorption spectra of twelve purified semiconducting CNT species along with their (*n, m*) structural notations. Adapted from [169]. **b** Molecular dynamics model of (8,4) nanotube obtained by rolling a 2D DNA sheet with ATTTATTTATTT strands. Orange color indicates thymine, green color indicates adenine and yellow color shows the backbones. Adapted from [169]. **c**, **d** Chirality separation of SWCNTs using allyl-dextran-based multi-column chromatography. **c** Using SDS as a single surfactant, the dispersed SWCNTs were adsorbed on column medium and, upon saturation, the single-chirality tubes are enriched according to its binding affinity towards the column. Adapted from [176]. **d** Bulk separation of iterative column chromatography to produce single chirality enriched SWCNTs, showing their distinct colors according to their chirality. Adapted from [176]

Another separation technique, referred to as the ion-exchange chromatography (IEX), is based on the ion-exchange processes occurring between a mobile phase and stationary ion-exchange groups (which are bonded to the support material). The IEX separation method is carried out on single-stranded-DNA-wrapped (ssDNA) SWCNTs, which have different electrostatic interactions with an ion exchange column [169, 170]. By selecting the desired sequence from the vast ssDNA library, purification of the specific (n, m) species was possible. With certain ssDNA sequences greatly improving separations between metallic and semiconducting CNTs as well as between semiconducting CNTs of different diameters and electronic band gaps [171]. The IEX process begins by wrapping ssDNA around individual SWCNTs, to form DNA/CNT hybrids. Some of the DNA/CNT hybrids in aqueous dispersions are electrostatically bound to the positively charged anion-exchange resin (stationary phase). As the mobile phase is passed over the hybrid-resin system, and its ionic strength increases, hybrids with the lowest effective charge density elute within the shortest IEX times. Because the hybrids are found in both the stationary and mobile phases, the separation is based on differences in this distribution. There is less electrostatic attraction between metallic hybrids and the IEX resin than between semiconducting hybrids and the IEX resin, thus in a mixture of metallic and semiconducting CNTs of the same diameter, the metallic hybrid will elute from the column first. This method of DNA-wrapped CNTs produced many single-chirality semiconducting CNTs. Figure 22a shows the optical absorption spectra of 12 purified semiconducting SWCNTs along with their structure. This method could also be used for purification of armchair metallic tubes [133, 169]. An alternative approach to sort metallic and semiconducting CNTs is using anion-exchange chromatography technique. Here, single-stranded DNA form stable complexes with CNTs and can effectively disperse them in water. Here, the chosen DNA sequence self-assembles into an ordered structure around an individual nanotube, helping in nanotube formation (Fig. 22b).

Gel chromatography, particularly, agarose gel chromatography is a method of separating semiconducting CNTs from metallic CNTs in an mass-spectroscopy mixture using hydrogels [172, 173]. Agarose gel beads are used for mass-spectroscopy separation, owing to their simplicity, affordability, short process time of about 20 min and scalability. The mechanism for gel chromatography follows a few simple steps. First, the SWCNT mixture, containing both metallic and semiconducting CNTs, would be dispersed in an aqueous surfactant solution, such as sodium dodecyl sulfate (SDS). The wrapping and encapsulation of the SDS surfactant molecules around SWCNTs plays a crucial role in the separation mechanism. The interaction between SDS molecules and SWCNTs via ion-dipole forces depends on the pH condition and concentration of SDS molecules. Due to the electrostatic properties of SWCNTs [174], SDS molecules form different types of micellar structures around semiconducting and metallic SWCNTs [172, 175]. On semiconducting CNTs, randomly oriented, flat micellar structures are formed, while for the metallic CNTs, cylindrical micellar structures are formed. This is mainly due to difference in ion-dipole forces between metallic and semiconducting CNTs during their adsorption on agarose gel. These disparate encapsulation mechanisms form the basis of the separation process. After the SWCNT dispersions are formed, they are ultra-centrifuged to remove SWCNT bundles and other impurities, and the SWCNT-surfactant solution is pipetted to be used in the separation process. Next, a separating column is filled with agarose micro-beads suspended in ethanol, after which the column is washed and equilibrated using the surfactant aqueous solution. The agarose-SWCNTs mixture, which is to be separated, is then poured into the column, and the SDS solution is added. This causes a displacement of the SWCNT dispersion along the column. A portion of the SWCNTs (the semiconducting CNTs) are trapped at the top layer of the agarose beads, while the metallic CNTs move to the bottom of column. This movement is related to the encapsulation of the tubes. Because semiconducting SWCNTs are encapsulated by flat randomly oriented SDS micelles, and have less surfactant coverage, there will be an ineffective shielding between the semiconducting SWCNTs and the agarose gel, and thus, a stronger affinity of the semiconducting SWCNTs to the gel. However, the metallic SWCNT walls are surrounded by an ordered high-density cylindrical micellar structure, which causes a steric hindrance between the SWCNTs and the agarose gel. Therefore, the metallic tubes have less affinity to the agarose gel. A schematic of SWCNTs separation based on the chirality of the tubes is shown in Fig. 22c, d [176].

**Fig. 23:**
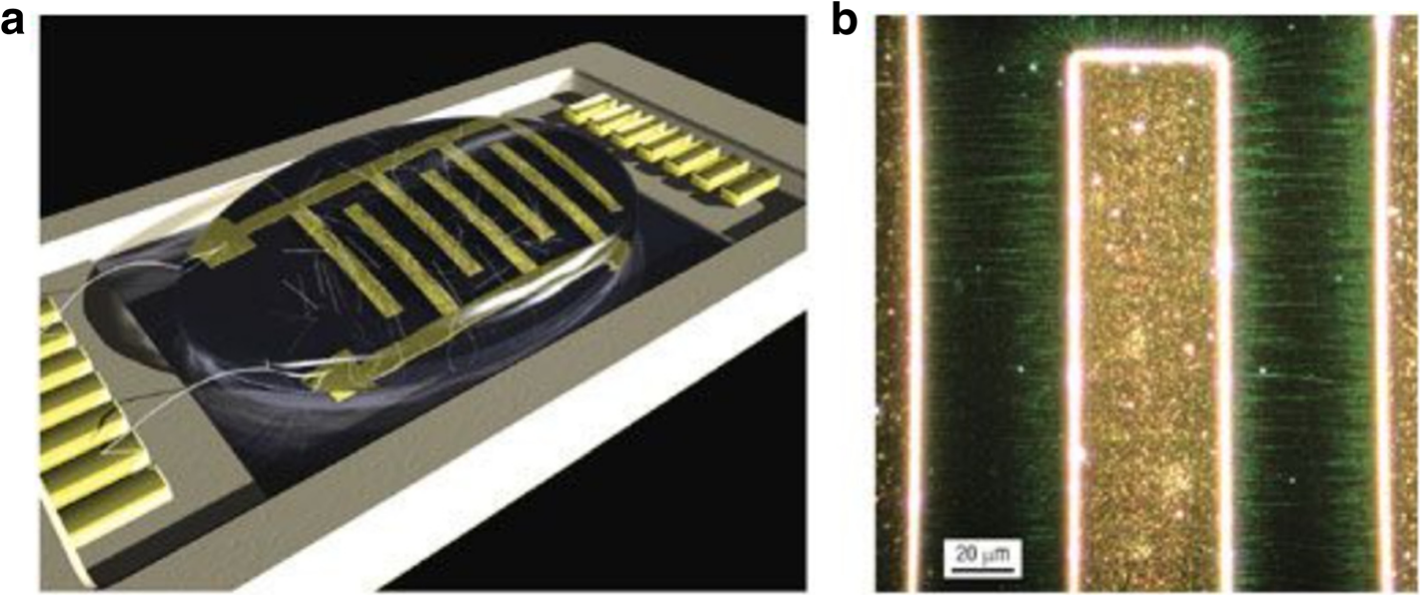
**a** Schematic of the experimental setup of the dielectrophoresis of a SWCNT solution using a microelectrode array. The metallic tubes (in black) are deposited on the electrodes and semiconducting tubes are left in suspension (in white). Adapted from [182]. **b** Rayleigh scattered dark-field micrograph showing aligned SWCNTs (in green) and the corresponding polarized SWCNTs perpendicular to the electrodes. Adapted from [182]

Another technique to separate metal and semiconductor nanotubes is using the technique of dielectrophoresis (DEP). When a particle is placed in an electric field, a lateral force, also known as a dielectric force acts on it [177]. This force can be used to manipulate nanoparticles or cause them to move, and the resulting movement of particles is termed dielectrophoresis [178]. The operating principle of the alternating current (AC) DEP process is based on the fact that metallic and semiconducting CNTs have different dielectric constants. The setup consists of a fabricated microelectrode, fluidic chamber and the SWCNT solution. The DEP force is generated by applying a non-uniform electric field to the setup. Due to the applied electric field, a dipole moment is induced on the SWCNT mixture, and the tubes will move towards the maxima or minima of the electric field depending on their polarity. Under the action of an AC electric field, CNTs in solution will move to the electrodes depending on their surface charge [179–181]. The electrodes are typically fabricated using e-beam lithography, which are then attached to a function generator. When an AC electric field originating from the function generator operating at 20 V peak-to-peak voltage and a frequency of 10 MHz is applied, a suspension of ~ 10 μL of SWCNTs is deposited. The metallic nanotubes will attach themselves to the electrodes, while the semiconducting tubes will remain in the suspension (Fig. 23) [182]. This is due to the divergent responses of the different types of CNTs to the electric field. In this technique, direct current (DC) electric field is not usually used as it leads to aggregation of CNTs near one of the electrodes [179]. The applied electric field and deposition time are the crucial parameters which control the CNT deposition yield.

Gel electrophoresis was developed as an improvement to the AC dielectrophoresis method. This process makes use of the same mechanism as AC electrophoresis but uses agarose gel as a medium. SWCNTs dispersed in an aqueous SDS surfactant are used to fill a gel column and subjected to an electric field. This causes a movement of the m-SWCNTs through the gel medium to the anode while the s-SWCNTs are adsorbed to the gel [177, 178].

Sorting of CNTs can also be done using solution-based conjugated polymers which can be used for selecting pure semiconducting SWCNTs from CNT samples. Here, semiconducting CNTs are wrapped with conjugated polymers, and this technique is considered helpful for selective and large-scale sorting of CNTs [183]. In this method, the SWCNT-polymer mixture is sonicated in an organic solvent for half an hour in order to disperse the SWCNTs. Next, the polymer-wrapped SWCNT solution is centrifuged for about an hour, which results in the sedimentation of m-SWCNTs. Finally, the s-SWCNT supernatant/liquid, which is found lying above the m-SWCNT sediments, is collected for use [183].

In another technique, a gas-phase plasma hydro carbonation reaction is used to selectively etch and gasify metallic nanotubes, retaining the semiconducting nanotubes in near-pristine form [139]. In this method, an array of 98 devices each consisting of ~ 0–3 as-grown SWCNTs grown using CVD were fabricated on an oxide-coated Si substrate. Each SWCNT was of ~ 1–2.8 nm in diameter. These arrays consisted of 55% semiconducting tubes which were non-depletable by the sweeping gate voltage, and about 45% metallic tubes which were depletable with on/off conductance ratio of ≥ 10^3^. These arrays were exposed to methane plasma at 400 °C and then annealed at 600 °C in a quartz tube furnace. Post this, it was observed that the metallic CNTs were selectively removed and the semiconducting tubes were left behind in a greater proportion of about 93%.

## Assembly/Placement/Integration of Multiple CNTs

Integrating multiple CNTs is essential for the realization of large-scale device applications. This has proved challenging due to the need for precise control and positioning of the fabricated CNTs with respect to other device elements. In this section, we focus on some of the existing techniques that are used in the process of batch level control, fabrication of multiple CNTs and their subsequent integration onto the substrates.

### Batch Level Control

#### Catalyst Patterning

During CVD, a catalyst is often dispersed on the substrate from a solution containing a suspension of the nanoparticles. This is done by spin coating the substrate or by dipping the substrate into the catalyst solution. Alternatively, catalysts can also be deposited on the substrates by evaporation to create thin films. In order to position the catalysts at specific locations, different lithographic techniques like photolithography and microprinting have been reported.

**Fig. 24:**
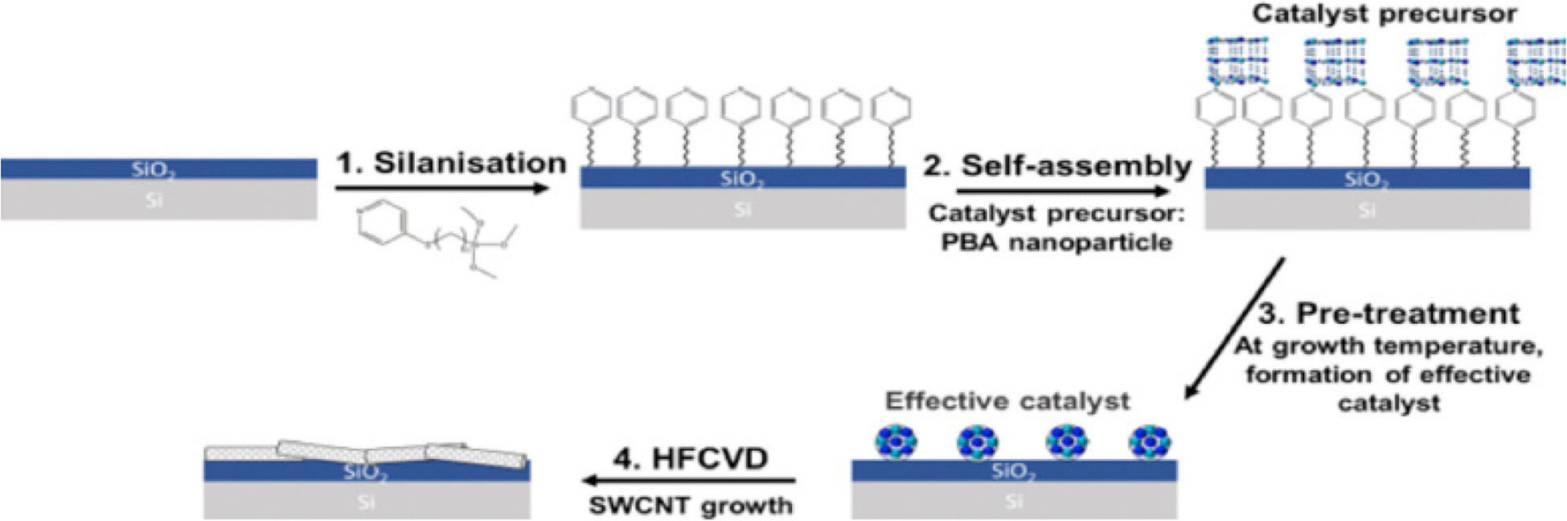
Schematic of the steps followed in the methane CVD growth of SWCNT using PBA-based bimetallic nanoparticle catalysts. In this technique, SWCNTs with diameters in the range of 0.7 nm to 2.6 nm were grown on silicon substrates coated with an oxide layer onto which self-assembled silane molecules were deposited. Adapted from [188]

Photolithography is used to pattern the catalyst which leads to growth of CNT thin films after lift-off. In one of the methods, controlled growth of CNTs with diameters of 0.5–1.5 nm was reported using Fe salt as catalyst. In this work, photolithography produced liquid catalyst islands on polymethyl methacrylate (PMMA) and alumina substrates. However, most of the CNTs grown were randomly oriented [184, 185]. Self-assembled masks can also be used to pattern catalysts in solution in order to control the positioning and alignment of nanotubes [186]. Another work reported the controlled growth of CNT thin films in certain regions by catalyst particle patterning using self-assembled monolayers. Here, a thick silicon substrate was thermally oxidized and positive photoresist mesas where CNT thin films were formed were patterned [187]. In a recent work, the growth of SWCNTs with diameters in the range of 0.7 nm to 2.6 nm using Prussian blue analog (PBA)-based bimetallic catalysts was reported [188]. Control on the overall catalyst size and properties was possible by synthesising PBA nanoparticles with narrow size distribution. Silicon wafers coated with an oxide layer were used as substrates. On these, a self-assembled monolayer of silane molecules (having a pyridine group at the ends) was deposited in order for the bond formation with the PBA nanoparticles to occur. Catalyst precursor reduction and the SWCNT growth were done via CVD with CH_4_ (Fig. 24).

**Fig. 25:**
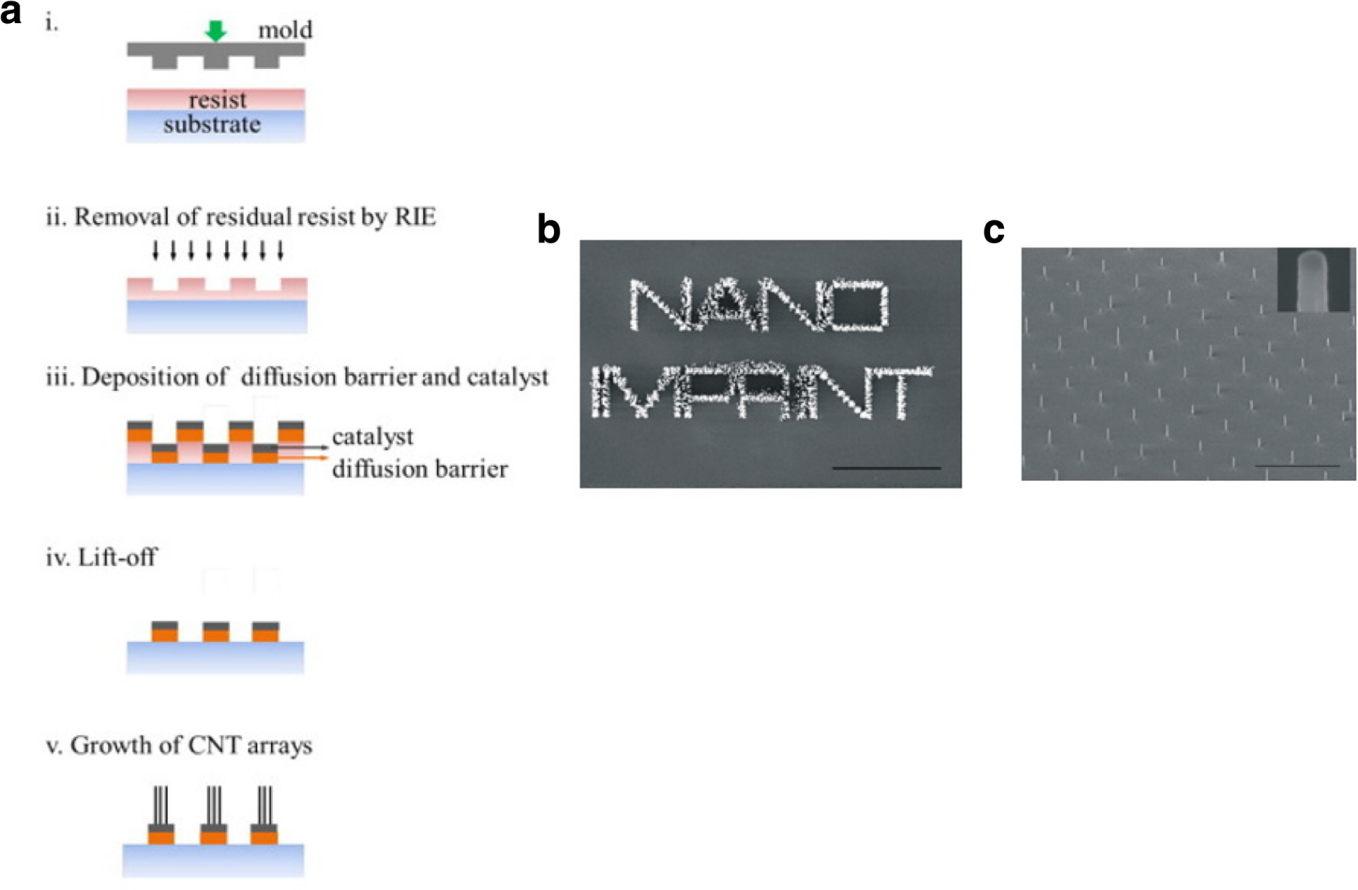
**a** Schematic of steps involved in the growth of CNT arrays using NIL. Adapted from [193]. **b**, **c** SEM images of CNTs grown using NIL. **b** CNTs arranged in a word format reading ‘Nano imprint’. Scale bar equals 20 μm. Adapted from [192]. **c** An array of CNTs with 10 μm spacing. Inset shows the tip of an individual MWCNT grown using Ni catalyst. Adapted from [192]

Nano-imprint lithography (NIL) is another technique for patterning the catalyst [189]. This technique can be used to produce CNTs (in the form of both individual tubes and arrays or forests) with sufficient degree of control over diameters, length and quality [190, 191]. NIL uses silicon molds/stamps with different patterns of nanoscale features to imprint a desired pattern onto a polymer-based thermal resist. After this, required pressure and ultraviolet (UV) light are applied to solidify the polymer resist and form desired circuit patterns. In some cases, temperature can also be applied to the photoresist instead of UV light. Later, the stamp is removed from the resist which leaves behind an imprint of the desired patterns on the substrate. The residual layer of polymer is removed by plasma etching, thereby exposing the substrate onto which the catalyst is deposited. This substrate is loaded into CVD to grow patterns of CNTs. An example of this step-by-step procedure and the corresponding scanning electron microscope (SEM) images of CNTs grown using NIL is shown in Fig. 25 [192, 193].

New techniques using nanolithography like nanowriting with nanopipettes [194] and dip-pens [195] help in the growth of CNTs at predetermined locations. For example, in the dip-pen method, the tip of an atomic force microscope (AFM) is usually dipped in an ‘ink’ that can subsequently be transferred to a substrate with nanometer-scale precision. Similarly, nanowriting provides direct and precise control over surface patterning without requiring complex lithographic processing [196].

**Fig. 26:**
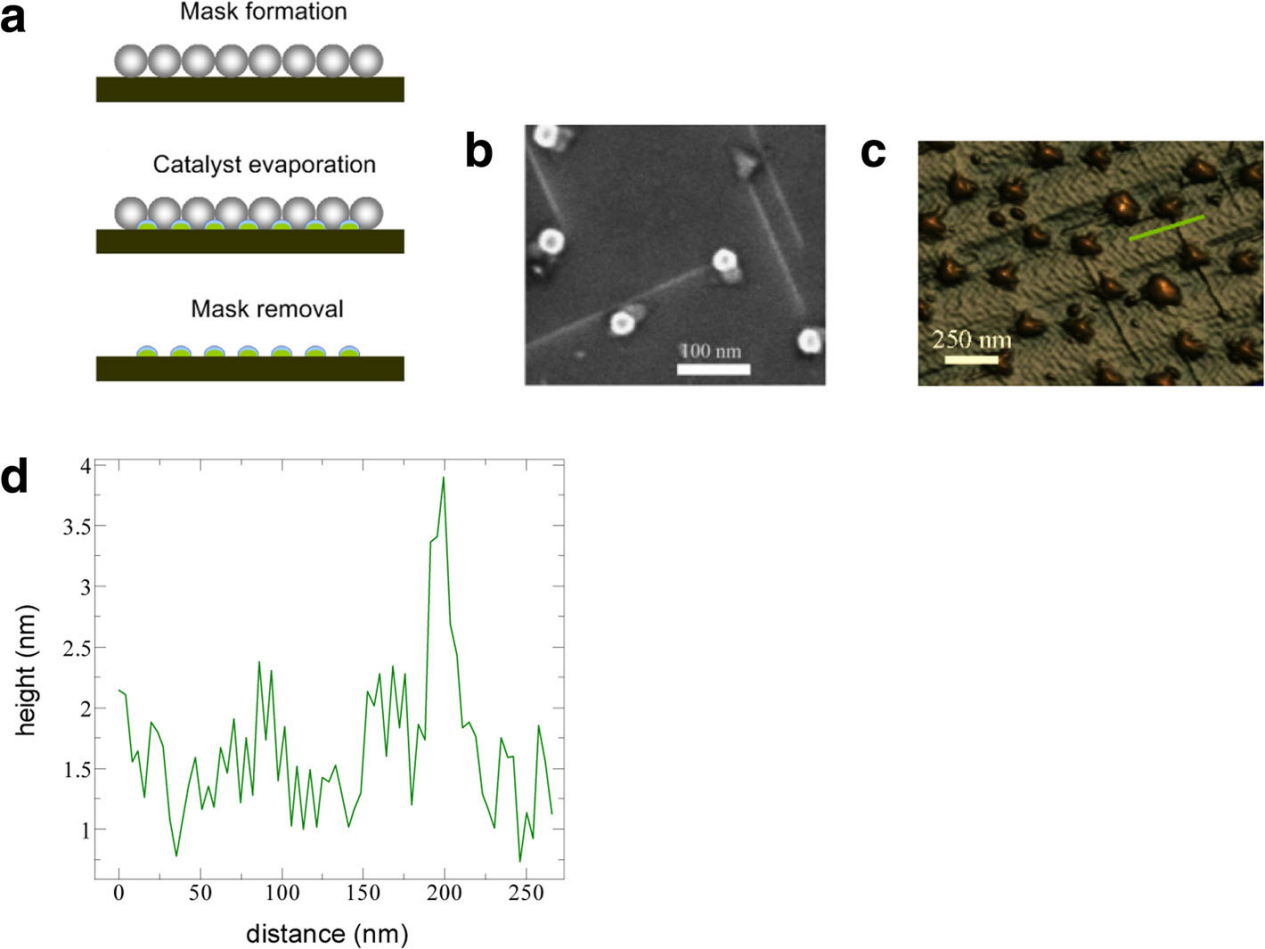
**a** Schematic of steps involved in fabrication of patterned catalyst array on undoped Si substrates using colloidal lithography. The spheres represent polystyrene spheres with a diameter of 450 nm. Adapted from [197]. **b** SEM image of the individual SWCNTs connected between catalyst patterned nanoparticle arrays. Adapted from [197]. **c**, **d** AFM image of individual SWCNTs with diameter of ~ 2 nm. Adapted from [197]. Green line shows the **d** corresponding cross-section. Adapted from [197]

Controlled production of large-area SWCNT networks can also be done using precise nanometer-scale catalyst patterning resulting in desired alignment of individual SWCNTs on silicon [197]. In this method, the catalysts act as a breadboard that connects the nanotubes with desired alignments. Here, a colloidal mask was used to pattern catalyst nanoparticles using polystyrene spheres that were deposited from liquid suspension and allowed to self-assemble during drying into hexagonal close-packed monolayer regions as shown in Fig. 26.

**Fig. 27:**
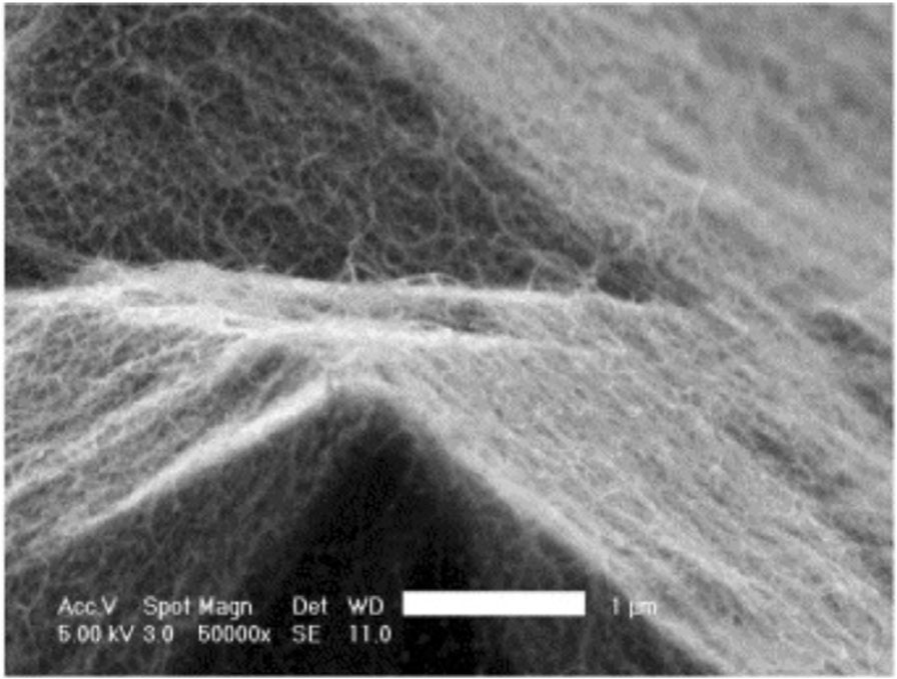
Schematic of a catalyst patterning technique which involves the control of growth direction of CNTs by partially patterning the catalyst layer on slanted KOH-etched edges. The corresponding SEM image of CNT pillars grown on the pyramid inside the KOH-etched microchannel is shown. Adapted from [198]

Additionally, catalyst patterning can also be used to control the growth orientation of CNTs during CVD by patterning the catalyst layer on slanted surfaces etched using potassium hydroxide (KOH) as shown in Fig. 27 [198]. In this technique, the catalyst is patterned fully or partially on slanted trenches fabricated via KOH etching. After this, the patterning of a catalyst layer (of 1 nm Fe and 10 nm Al_2_O_3_) is carried on the sidewalls using lift-off and e-beam evaporation. Then, CVD is used to grow CNTs with the following conditions; growth was carried out at 775 °C for ~ 5 or 15 min).

#### Electric Field, Gas Flow and Substrate-Assisted Growth

**Fig. 28:**
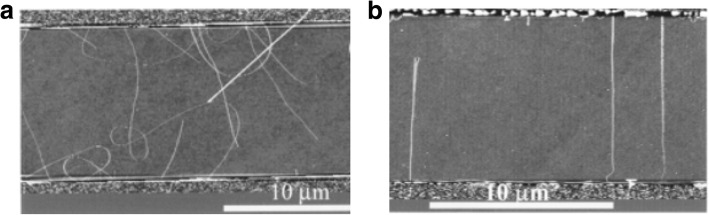
AFM images of CNTs grown using CVD technique between two Mo electrodes which are shown on top and bottom of the images. **a** Randomly grown CNTs in the absence of an electric field. Adapted from [199]. **b** Aligned grown in the presence of an electric field (a bias voltage of 10 V bias applied between the electrodes having a gap of 10 μm). Adapted from [199]

Controlled synthesis of CNTs can be achieved by growing them on the SiO_2_ /Si substrates in electric fields established across patterned metal electrodes [199]. In this technique, Si wafers were used with thermally grown SiO_2_ as substrates. Molybdenum (Mo) metal electrodes with a gap of 10 mm were used to establish electric fields on the substrates. Then, the desired catalyst was patterned on top of the two opposing Mo electrodes leading to the growth of aligned SWCNTs across the gap between the electrodes in the direction of the applied electric field. Figure 28 [199] shows the AFM images of randomly grown nanotubes in the absence of an electric field and aligned nanotubes grown in the presence of an electric field.

**Fig. 29:**
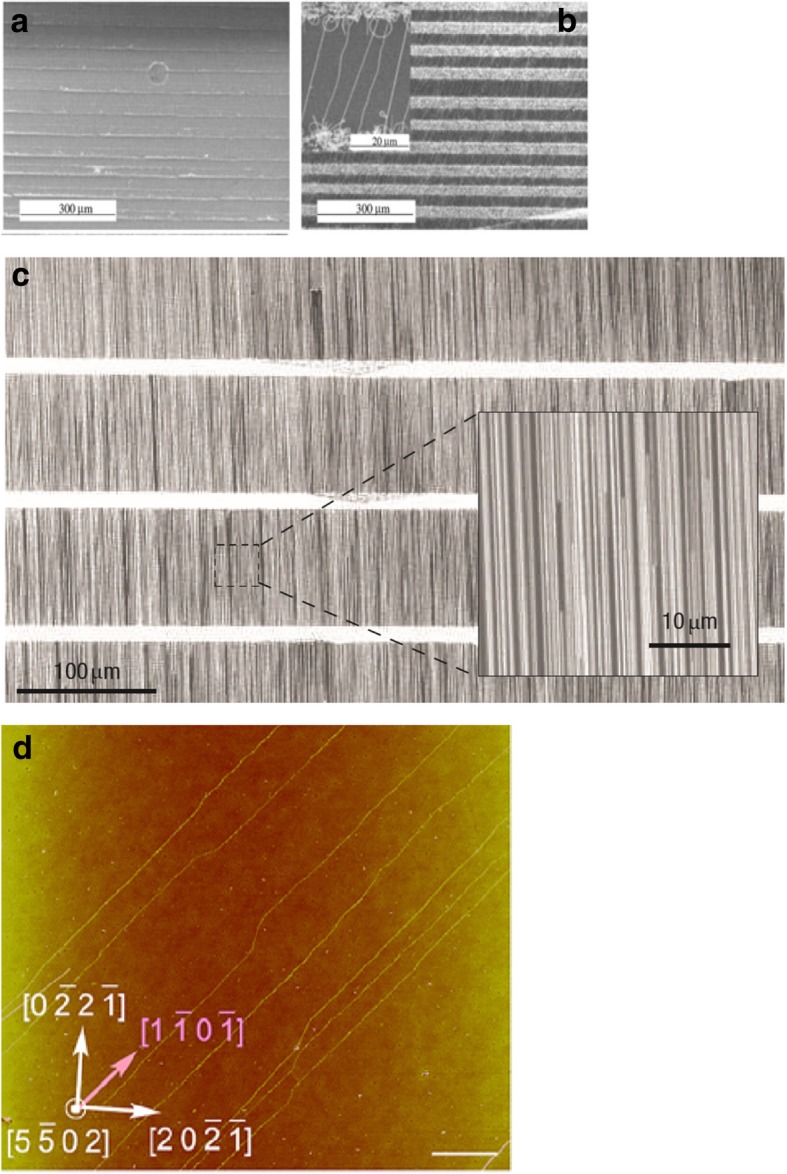
SEM images of (**a**) catalyst pattern seen on oxide coated Si wafer prior to the growth of SWCNTs. Adapted from [202]. **b** Long, well-oriented SWCNTs grown using fast-heating growth process with Fe/Mo nanoparticles, CO/H_2_ 900 °C for 10 min. Inset shows the magnified image of the SWCNT arrays formed. Adapted from [202]. **c** Well aligned arrays of SWCNTs (~ 5 SWCNTs μm^−1^) formed by CVD growth on a ST-cut quartz substrate. Adapted from [207]. **d** AFM image of aligned SWCNTs grown on r-plane (1 1 0 2) crystalline surfaces of sapphire. Adapted from [206]

Another method of controlling the growth of CNTs is based on rapid heating (900 °C for 10 min) of catalyst nanoparticles (Fe/Mo) in the presence of feeding gas (CO/H_2_) [200–202]. SWCNTs were grown parallel to the direction of feeding gas flow. This work reported directional control of the CNTs grown by positioning the substrate based on the gas flow direction. The location and length of SWCNTs was controlled by using photolithography to deposit the catalysts. This method produced ultra-long, well-aligned and well-isolated SWCNTs with length of few mm (Fig. 29) in contrast to an earlier work that reported that long SWCNTs (in the range of mm) either bend or form loops [203]. Here, the growth of long and straight SWCNTs was attributed to the above described growth process also termed as a kite-based growth mechanism [201].

Large scale, highly aligned SWCNTs arrays can also be grown by interactions between SWCNTs and the substrate [204–207] using atomic arrangement-programmed growth. Highly aligned SWCNTs could also be grown using Y-cut single-crystal quartz or ST-cut quartz substrates using CVD of methane at 900 °C and Fe catalyst. As shown in Fig. 29c, SWCNTs with diameters of 1 ± 0.5 nm were grown on quartz using this technique. In this method, SWCNTs were synthesized using CVD of methane at 900 °C with Fe clusters that were dispersed on the sapphire substrates. Alternatively, use of a-plane and r-plane sapphire (Al_2_O_3_) substrates led to guided growth along specific lattice directions due to the attractive interactions between nanotubes and Al atoms that are oriented in specific crystalline directions on the substrates (Fig. 29d).

#### CNT Forest Growth

CNT forests (or CNT arrays) are arrays of vertically aligned CNTs that offer applications in the field of sensors [208], gecko tapes [209], strong fibers [210] and electrical interconnects due to their ability to carry high current densities of ~ 10^8^ A cm^−1^ [211, 212]. Various techniques such as use of plasma CVD, nanopatterning and flying carpets have been proposed as means of growing CNT forests [210, 213, 214]. Of these, CVD is regarded as one of the most efficient techniques to grow vertically aligned CNTs. Precise diameter control of CNT forests using pre-growth conditioning and catalyst engineering is one of the crucial parameters that influences its use in various applications [215–217]. In addition, control of number of layers and length of the tubes grown is also essential [218].

**Fig. 30:**
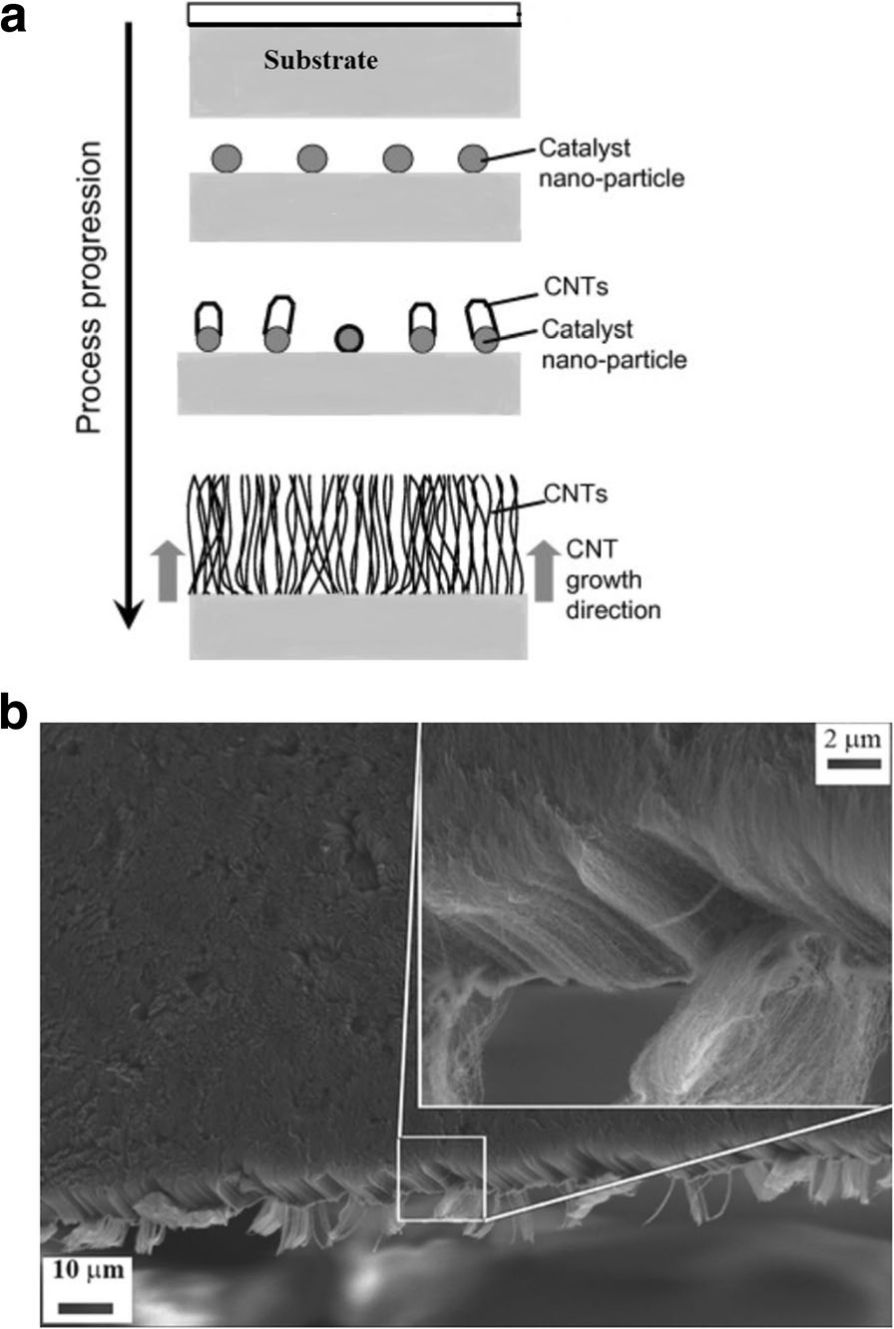
**a** Critical stages of CNT forest growth from a pre-deposited catalytic thin film using the multi-step plasma-induced CVD technique: formation of high-density plasma-induced nanoparticles, nucleation, and CNT forest formation. Adapted from [221]. **b** SEM image of the closely-packed MWCNT forest grown using plasma-induced CVD. Adapted from [222]

Growth of aligned MWCNT forests with diameters of ~ 8–15 nm was achieved using CVD in a quartz tube. Here, a Si wafer was used as a substrate, onto which a 5-nm-thick film of Fe (acting as a catalyst) was deposited using e-beam evaporation [219]. An alternate method for the fabrication of a closely packed MWCNT forest was reported using a multi-step growth method based on plasma-induced CVD technique [220]. The growth mechanism involves the following steps. First, very high-density plasma-induced catalytic nanoparticles were formed from a pre-deposited catalytic thin film. It was observed that these catalytic nanoparticles tend to aggregate at higher temperatures. In order to avoid this, the CNT nucleation process (immobilization procedure of catalytic nanoparticles) was performed and showed minimal aggregation of nanoparticles covered with graphitic carbon film. Finally, closely packed MWCNT forests were grown at a temperature of 450 °C (Fig. 30) [221, 223].

**Fig. 31:**
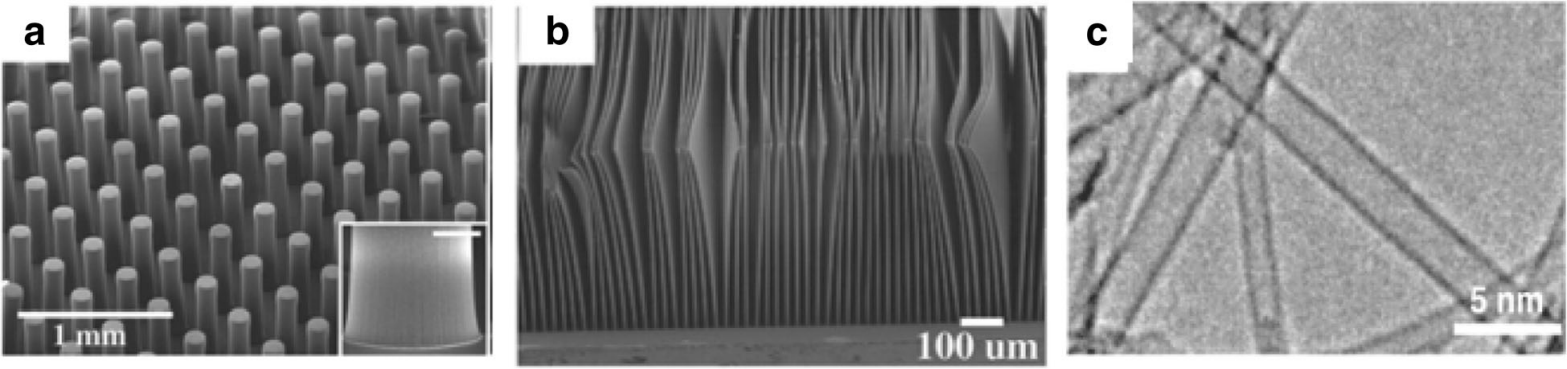
**a**, **b** SEM images of SWCNT forests grown using water assisted CVD. **a** SWCNT cylindrical pillars with 150 μm radius and ~ 1 mm height. Insert shows the root of a pillar. Adapted from [135]. **b** SWCNT sheets of 10 μm thickness. Adapted from [135]. **c** High-resolution TEM image of the SWCNTs grown using this method. Adapted from [135]

The direction of alignment of CNTs with respect to the substrate plays a crucial role in controlling the properties of CNTs grown. CNTs aligned vertically (i.e. perpendicular) to the substrate can be produced using porous silicon substrates with a catalyst patterned by electron-beam evaporation through shadow masks to produce MWCNT blocks. Another technique to produce these CNTs is using water-assisted CVD that produced SWCNT arrays in the order of few millimeters [135]. Here, large-scale production of dense CNT forests was reported to be possible with the help of CVD synthesis where the performance and lifetime of the catalysts was enhanced with the help of water. SWCNTs in the form of well-ordered pillars with a height of ~ 1 mm were grown from lithographically patterned catalyst islands as shown in Fig. 31 below. Alternatively, large-scale production of vertically aligned SWCNT forests is also possible using the oxygen-assisted CVD method. It was reported that the hydrogen species present during hydrocarbon CVD growth method may cause difficulties in the formation of new SWCNTs and can also potentially etch preformed SWCNTs. The introduction of oxygen during the growth process provides great control over the carbon and hydrogen ratio which is responsible for the growth of vertically aligned SWCNTs [223]. More recently, it was also shown that SWCNT forests could be grown using thermal CVD without a rectated etchant gas [224].

**Fig. 32:**
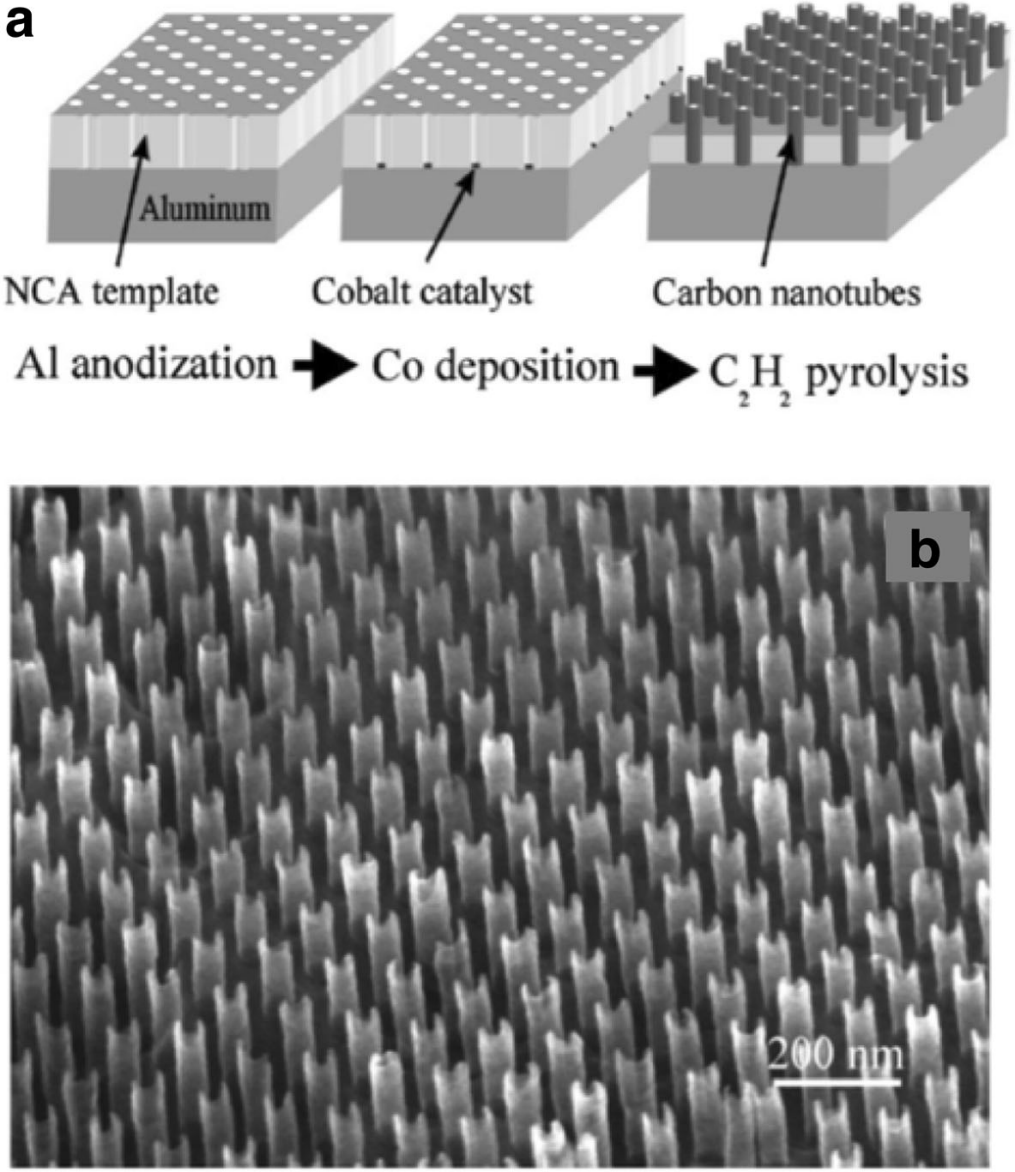
**a** Mechanism of growth of highly-ordered arrays of parallel CNTs using nanochannel anodic alumina templates. The process begins with the anodization of high-purity aluminum on the substrate. By varying the anodization conditions, hexagonal close-packed arrays of varying diameters, densities and lengths can be formed. Adapted from [227]. **b** SEM image of the resulting hexagonally ordered array of CNTs fabricated using the method in (**a**). Adapted from [227]

Another technique involves the fabrication of the CNTs within the pores or channels of a nanoporous template [160, 225, 226]. The commonly used templates are track-etch membranes, porous alumina (AAO) templates as well as various other nanoporous structures. Template-based synthesis allows the preparation of nanomaterials with a desired shape. A template is basically a structure in which the CNT networks form. Once the template is removed, it exposes a filled cavity with features similar to those of the template. After deposition, the nanotubes may be allowed to remain inside the pores of the templates, or they can be collected as a group of free nanoparticles. The template-based deposition process commonly makes use of CVD techniques, in which hydrocarbon precursors like pyrene and ethylene are exposed to elevated temperatures inside alumina templates. As the thermal deposition of the gas occurs over the entire surface of the pores, this method offers considerable control over the length and diameter of the tubes. One example of the growth of highly ordered arrays of parallel CNTs inside a hexagonally close-packed nanochannel alumina template is described below. At first, the anodization of high purity alumina was carried out, and led to the creation of a nanochannel alumina template made up of hexagonal array of channels with a diameter of 32 nm and length of 6 μm. Next, a small amount of catalyst (Co) was deposited electrochemically into the bottom of the template channels. By heating these templates at 600 °C for 4–5 h in a tube furnace in the presence of CO, then in a mixture of acetylene in nitrogen at 650 °C for 2 h, growth of highly ordered array of nanotubes was observed (Fig. 32) [160, 227]. Periodic array of nanotubes with diameters ranging from 10 nm to several hundred nm were grown by pyrolysis of acetylene on Co at a temperature of 650 °C.

Another method in the growth of SWCNT forests, involves the introduction of gas shower system to deliver water vapor and carbon source gas from the top of the forests, rather than traditional way of delivering from the side. This method enables parallel flow of water vapor and carbon source gas to the CNTs within the forest. This technique enables mass production of CNT forests with increased growth yield, height, carbon efficiency and scalability for large area growth [214, 228].

### Macrostructure Fabrication

**Fig. 33:**
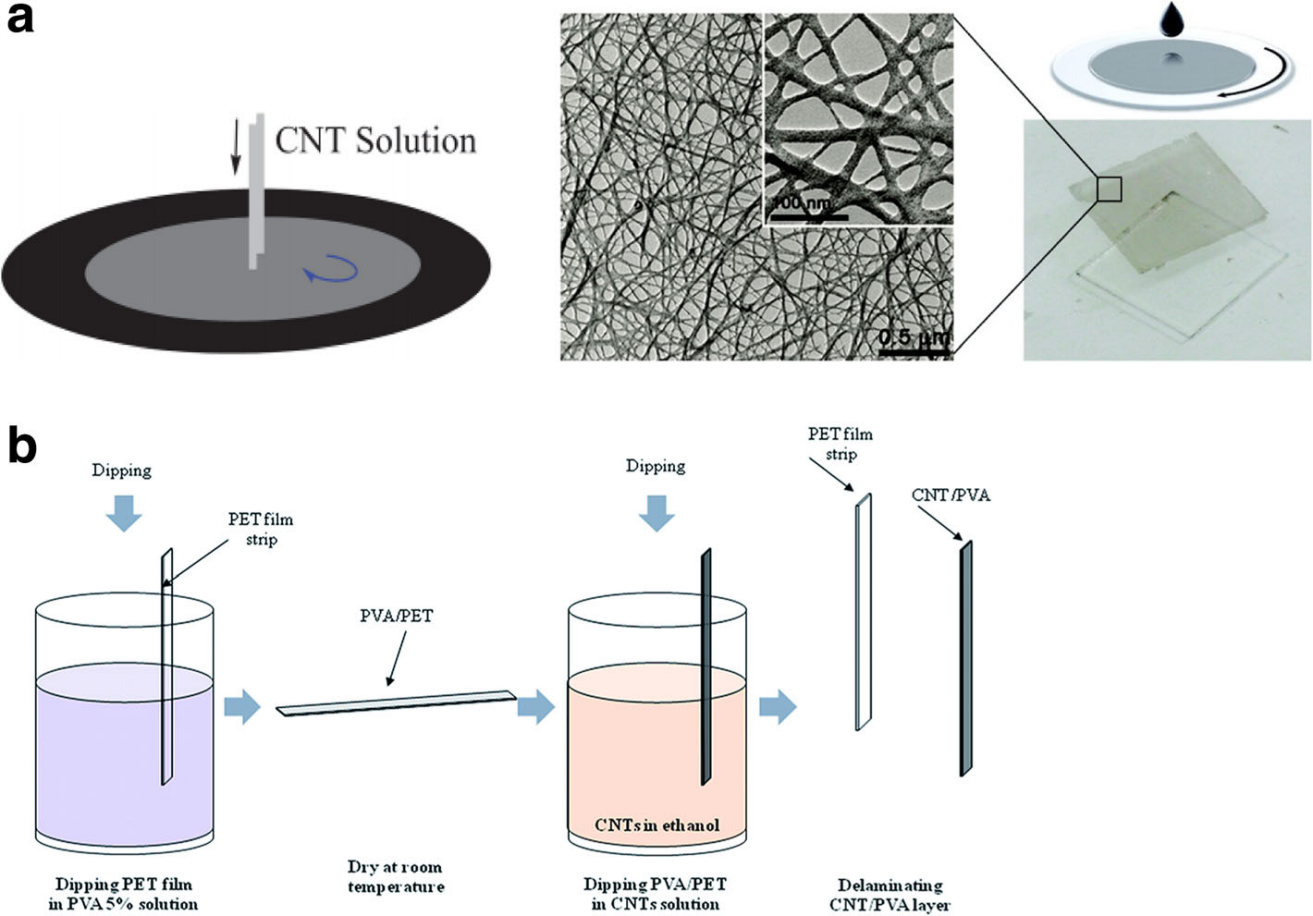
**a** Schematic of spin coating process for the fabrication of thin film of CNTs. Adapted from [230]. The corresponding SEM images of the SWCNT film prepared by single deposition of spin coating the substrate with CNT solution in an organic solvent called quinquethiophene-terminated poly (ethylene glycol). Adapted from [229]. **b** A schematic of a CNT-polymer composite fabrication via solution-based processing. Adapted from [232]

Fabrication and assembly of CNTs on a macroscale is of utmost importance in order to enhance its practical applications [179]. A commonly reported technique is using spin coating. This process of fabricating CNT films involves depositing droplets of CNT-based suspensions at the center of a substrate and then spinning the substrate to a very high velocity such that the CNT dispersion spreads out on the surface of the substrate, forming a thin film (Fig. 33a) [229, 230]. This process is due to the centrifugal force on the CNT dispersion. The thickness of the film depends on the viscosity of the dispersion, angular speed, spin time and the concentration of CNTs. Before the CNT dispersion is formed, it is important to overcome the Van der Waals forces between the tubes, else they will tend to aggregate and form clumps on the film. Amphiphilic surfactants are usually added to enhance the dispersibility of the tubes; conventional surfactant will not suffice because there is a strong charge repulsion between the CNT complexes and the surfactant, which prohibits the deposition of CNTs onto the substrate. Additionally, high-power ultra-sonication or strong-acid treatment is usually performed on the tubes. After the CNT film is deposited, the surfactant is usually either washed off or vaporized from the surface of the substrate. Spin coating is not applicable for large substrates because they cannot be spun at a sufficiently high rate to form an even layer. Additionally, the process lacks material efficiency as a lot of the material is flung off during spinning [229]. Alternatively, electrophoresis process can also be used for the film deposition of CNTs [231]. Some of the other relevant techniques include using composites [232], foams [233], yarns [210] and aerogels [234] as discussed below.

#### CNT Composites

Due to the various properties that CNT’s exhibit, especially being superior in stiffness and strength, they can be widely used in structural applications. Furthermore, researchers are showing increased interest in tapping CNT’s properties by fabricating them into a polymer matrix. Some of the commonly used CNT-based polymer composites are made up of polystyrene, ultra-high molecular weight polyethylene (UHMWPE), PMMA, epoxy and phenolic resin [62, 235–237]. Various techniques have been proposed for fabricating CNT-polymer composites based on the material combinations used. Some of the common methods like solution casting, melt mixing and in-situ polymerization are discussed below.

Solution casting, also referred as solvent casting, facilitates CNT dispersion, where CNTs are suspended in the desirable polymer solution via magnetic stirring or sonication (energetic agitation process). The solvent is then allowed to evaporate to produce CNT-polymer composites. Polymers such as polyvinyl alcohol (PVA) [238], PMMA [239] and epoxy [236] have been used in this method. One such manufacturing technique is shown in Fig. 33. Here, PVA/CNT composite fibers of ~ 50 μm diameter were synthesized using the following steps. First, CNTs were dispersed in ethanol, then a PVA-coated polyester strip was dipped in a solution containing the CNT mixture. Then, the CNT-coated PVA on the polyethylene terephthalate (PET) strip were delaminated. Finally, this delaminated layer was stretched and twisted to form a composite fiber (Fig. 33a). Studies involving different solutions and methods have demonstrated that the selection of solvent for nanotube dispersion had an influence on the properties of synthesized nanocomposites and the type of solvent is dependent on the solubility of polymer in it [240]. A major shortcoming of this method is that the nanotubes may agglomerate if the solvent evaporation process is slow. This may be mitigated by increasing the rate of evaporation [241]. This leads to inhomogeneous distribution of CNT’s in the polymer matrix [242].

Another commonly used method for the fabrication of CNT-polymer nanocomposites is called melt mixing. It is mostly used for manufacturing of thermoplastics and thermistors. Thermoplastic polymers melt/soften when heated to high temperatures thus making them less viscous. This causes the substrate to be less viscous and the need for high shear forces to disrupt the nanotubes bundle. In this method, composites of various polymers (such as polystyrene and polypropylene) are formed with CNTs. To start with, the selected polymers are mixed with nanotubes in a high sheer mixer. Post this, composite films are formed using compression molding [6]. Further studies in this area has enabled different techniques such as extrusion, compression molding, injection molding, etc. for fabricating samples of various shapes [6, 243, 244].

**Fig. 34:**
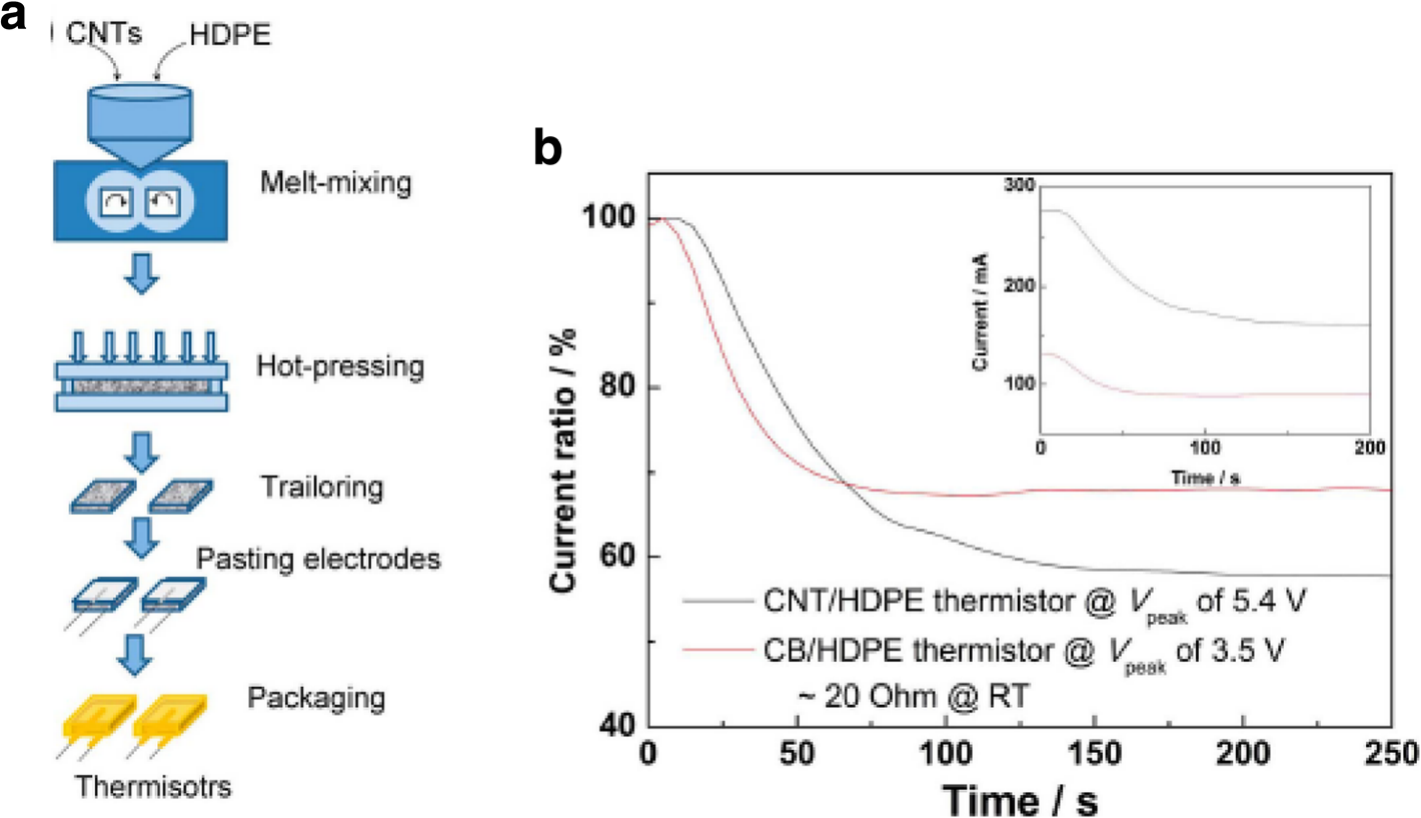
**a** Schematic of the fabrication of CNT/HDPE thermistors using melt mixing. The process consists of melt-mixing, hot-pressing and packaging. Adapted from [245]. **b** Graph comparing the current vs time plots of CNT/HDPE and CB/HDPE thermistors. The comparison of the current decay between the CNT/HDPE and CB/HDPE thermistors with the same resistances under peak applied voltages shows a quick response of current delay of the CNT/HDPE thermistors. Adapted from [245]

Recently, melt-mixing method was used in the manufacturing of positive temperature coefficient (PTC) thermistors using CNTs as conductive fillers in high-density polyethylene (HDPE) composites. Traditional PTCs manufactured using carbon black (CB) filled high-density polyethylene composites suffer from disadvantages like low thermal stability and poor processability. Use of CNTs in the manufacturing process can help in overcoming these challenges as the CNT-based thermistors showed ~ 129% increase in hold current and hold voltage, in comparison with the CB-filled composites [245]. These CNT/HDPE microstructure composites were prepared by melt-mixing, hot-pressing and packaging methods as shown in Fig. 34a. CNT/HDPE composites showed a decrease in electrical resistivity with an increase in the CNT loading. Figure 34b shows a comparison of current vs time plot obtained for CNT/HDPE and CB/HDPE thermistors.

**Fig. 35:**
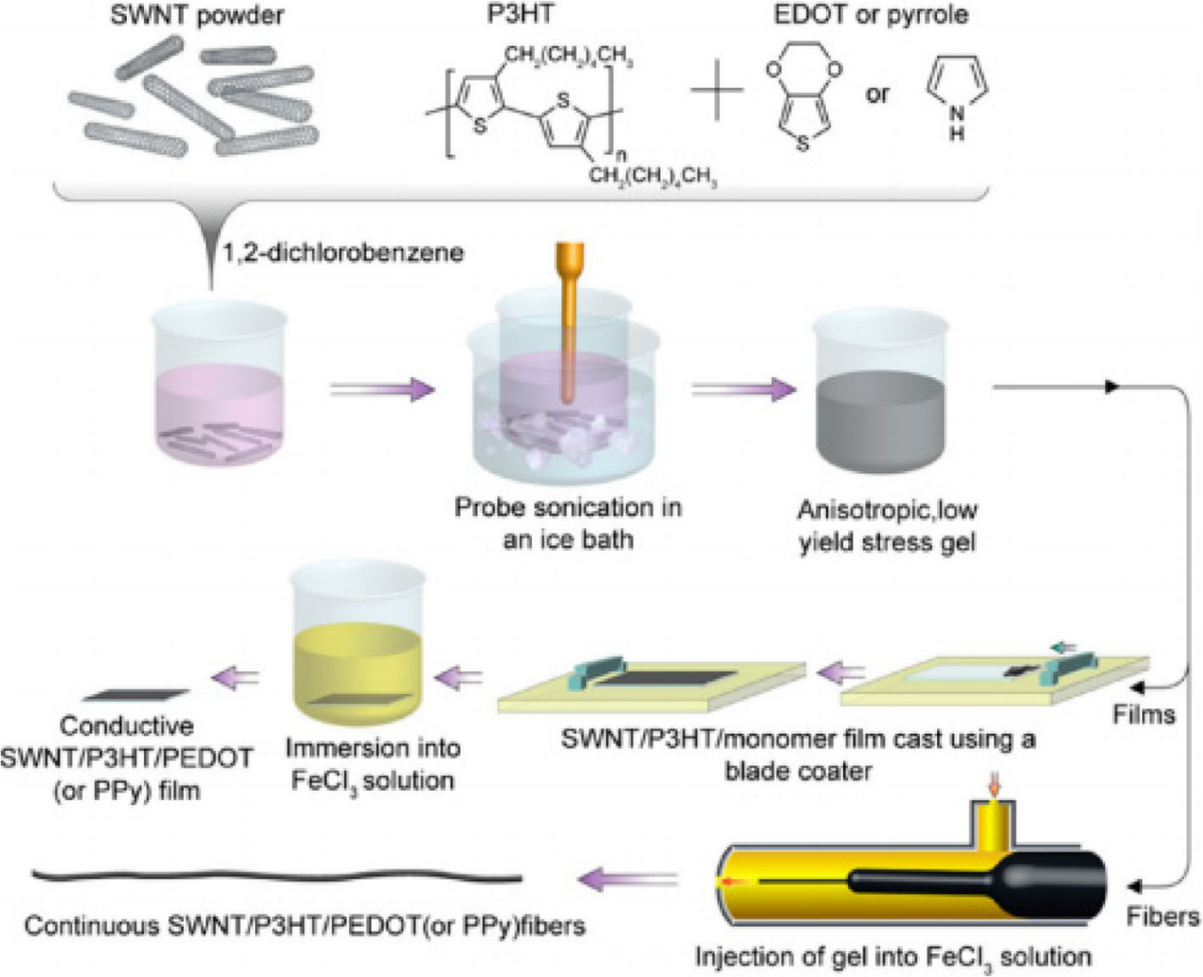
Schematic of formation of SWCNT/P3HT/PPy or SWCNT/P3HT/PEDOT conductive films and fibers using in-situ polymerization. The SWCNT/P3HT monomer dispersions were prepared and then coated onto a glass slide to form a film or extruded from a syringe to form fibers. Adapted from [249]

CNTs can also be fabricated with monomers or polymers of higher molecular weight using in-situ polymerization. In general, this method can be used in the fabrication of almost any polymer composite with CNT to polymer matrix that is either covalently or non-covalently bounded [246, 247]. In addition, this method makes the grafting of larger polymer molecules to the walls of CNT possible. This enables the fabrication using insoluble and thermally unstable polymers that cannot be achieved by solution or melt processing. A stronger interaction of CNTs with polymers during the growth stage due to π-bonding was observed in this technique [248]. One such schematic of fabrication of CNTs using in-situ polymerization is shown below in Fig. 35 [249].

Many other lesser known methods to fabricate nanocomposites have also been proposed such as twin screw pulverization [250]**,** latex fabrication [251], coagulation spinning [252] and electrophoretic deposition [253].

#### Foams, Yarns and Fibers

Three-dimensional porous networks made of polymers and CNTs have a great potential to be used in various applications like energy storage [254] and sensing [255]. Here, CNTs are used as modifiers into structures made of different porous materials like carbonaceous aerogels, foams and sponges [219, 234, 256]. Fabrication of CNT foams and aerogels can be done using several techniques like c-CVD, phase separations, polymerization reactions, etc. Creation of CNT foams and aerogels can be done using a bottom-up method consisting of phase separations induced by thermal phase transition. Here, a three-dimensional (3D) network of CNTs is initially formed in a solution, post which the liquid part is removed without disturbing this network. This technique is commonly referred as freezing as it involves a solid-liquid phase separation process. However, it was reported that CNTs grown using the various freeze-drying techniques lead to agglomerations [257].

**Fig. 36:**
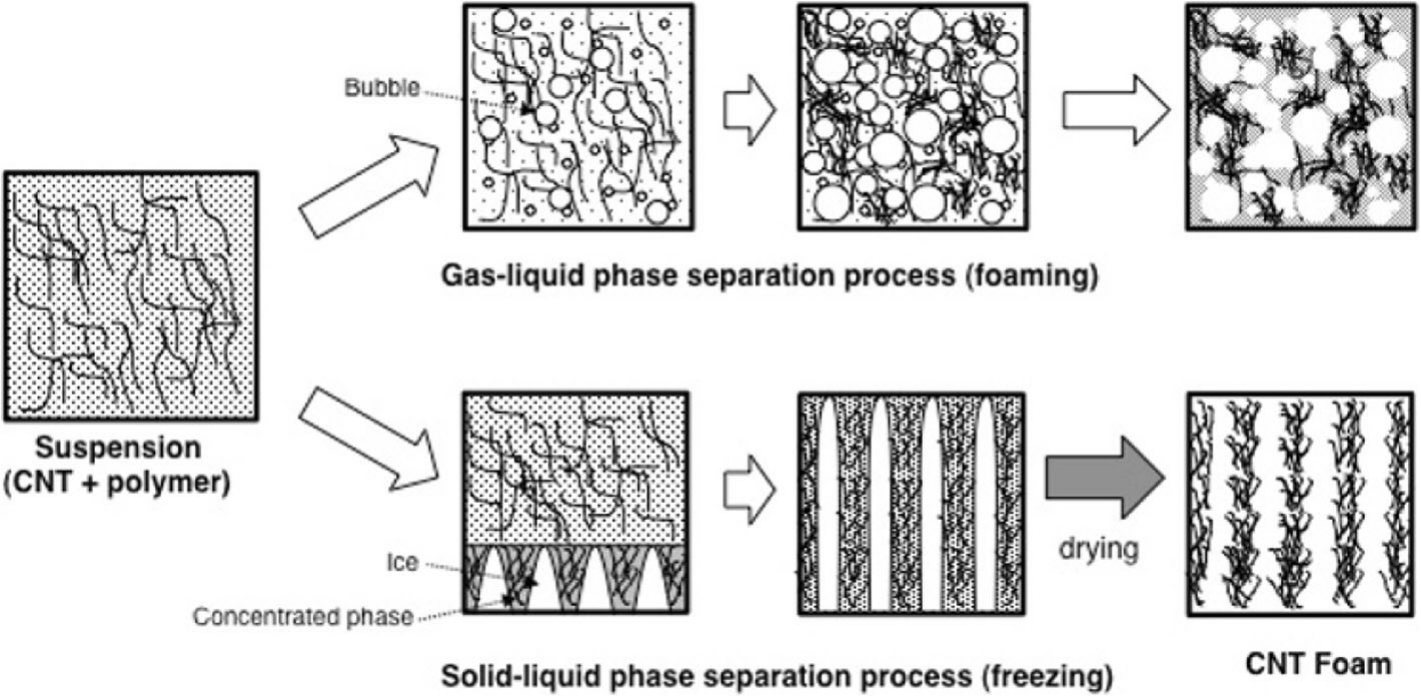
CNT foam formation using various techniques. The figure depicts various phase separation processes being performed on a CNT suspension to produce the CNT foam. The phase separation process is usually done via a gas-liquid phase separation or a solid-liquid phase separation as shown in the figure. Adapted from [258]

Another technique of CNT aerogel formation is using a gas-liquid phase separation process, commonly referred to as foaming as shown in Fig. 36 [258]. Fabrication of CNT foams with controlled pore structures using this foaming process can be done using PVA, polystyrene, polyurethane, etc. [259, 260] An alternate route to the fabrication of CNT foams is using CVD, wherein, by controlling the growth parameters like type of catalyst and source of carbon, vertically aligned CNT array foams could be formed [233, 260]. Recently, aligned CNT foam structures of desired size were fabricated by stacking sheets of CNTs and infiltrating the stacked sheet assembly with pyrolytic carbon (PyC) [233]. Here, vertically aligned MWCNTs were grown using CVD technique with FeCl_2_ as catalyst and acetylene as the carbon precursor. These aligned CNTs were drawn from arrays and collected around two rotating parallel glass rods in order to have less compacted macro-porous structures. The CNT foams were then coated with 20 to 100 cycles of alumina buffer layers (that pins the catalyst particles on the surface of nanotubes) to help in the formation of secondary CNTs leading to creation of junctions, or branched CNT networks [260, 261]. In this method, prior to CVD, a buffer layer made of Al_2_O_3_ is deposited onto the porous structure to promote secondary CNT growth (in the form of straight CNTs or coiled-CNTs) at the surface of the primary nanotubes and within the pores of the CNT foams [261].

**Fig. 37:**
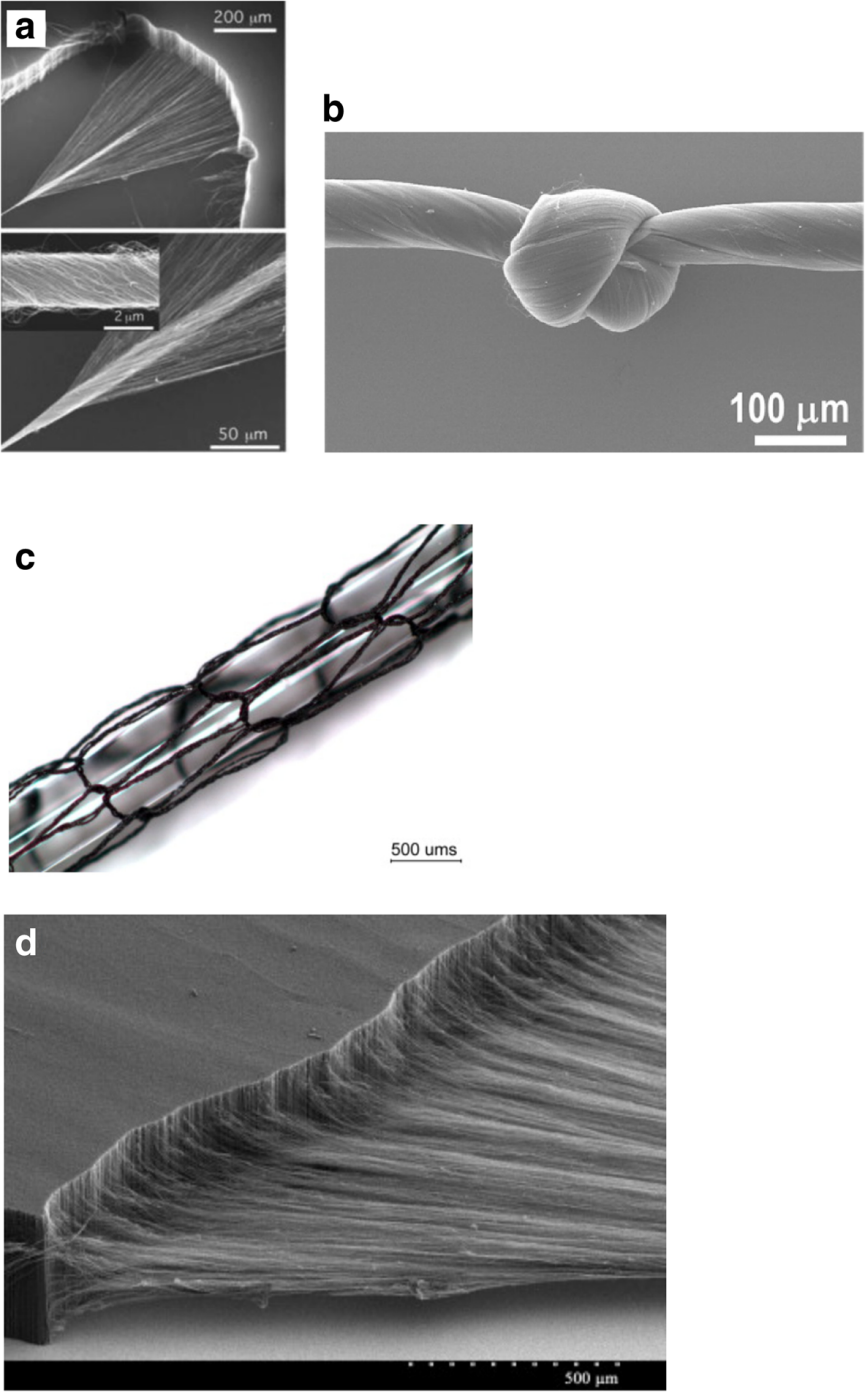
SEM and optical images of CNT yarns. **a** SEM image of a part of CNT yarn from the nanotube forest being drawn and twisted. Dry spinning of the CNT yarns from the forests (vertically grown CNT array) is shown. Adapted from [266]. **b** SEM image of a knotted CNT yarn. Adapted from [265]. **c** Optical image of a MWCNT knitted yarn, with a glass rod through the center. The average diameter of the MWCNT knitted yarn was measured to be 34.09 ± 2.86 μm. Adapted from [267]. **d** SEM image of MWCNTs in a forest being drawn into a sheet. Adapted from [270]

MWCNTs shaped into yarns have potential to be used in multifunctional applications like artificial muscles and actuators, in electronic textiles and as fiber-based supercapacitors [219]. Electromechanical actuators formed using sheets of SWCNTs (as electrolyte-filled electrodes of a super capacitor) are reported to have shown higher stresses than natural muscle. Like natural muscles, the macroscopic actuators are assemblies of billions of individual nanoscale actuators and are shown to have a great potential in various applications [39, 262, 263]. In addition, by twisting CNTs from a MWCNT forest, CNT yarns could be formed [219, 264]. Here, MWCNTs with a diameter of ~ 10 nm were drawn from a MWCNT forests (grown using CVD by using Fe catalyst) and twisted with a motor running a variable speed of ~ 2000 rpm [219]. This twisting causes the MWCNTs to form small individual bundles consisting of few CNTs. Further, by allowing the twisted yarns to relax (untwist in an opposite twist direction), knitted and knotted yarns were formed (Fig. 37a–c) [265–267]. Alternatively, MWCNT yarns could be synthesized from aerogels during CVD [268]. MWCNTs can also be drawn into sheets from MWCNT forests synthesized using CVD with ~ 3 nm iron film as a catalyst and acetylene or ethylene as carbon source [269]. These sheets were formed as a highly anisotropic electronically conducting aerogels that could be drawn into sheets with ~ 50 nm thickness as shown in Fig. 37d [270].

**Fig. 38:**
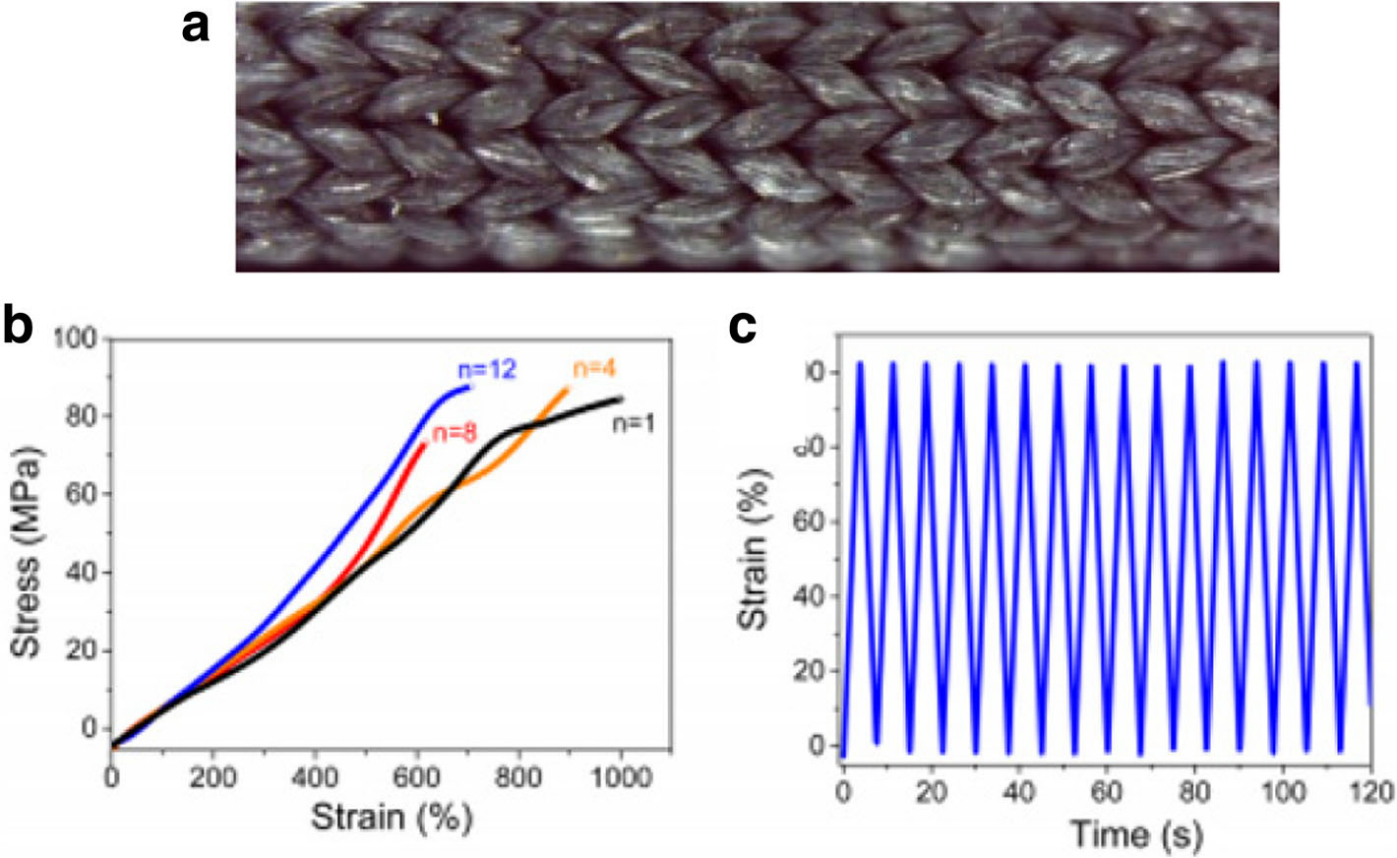
SEM image of CNT/SPX8 knitted textile and the corresponding mechanical properties. **a** SEM image of CNT/SPX8 knitted textile. Adapted from [271]. **b** Stress–strain curves of the CNT textile material. Adapted from [271]. **c** Strain versus time for this textile, stretched for 1000 cycles at 0.2 Hz. Adapted from [271]

Recently, electrically conducting 3D fabrics made from hybrid Spandex (SPX)–MWCNT yarns showed excellent stretchable properties with a potential to be used as artificial muscles (Fig. 38). They were fabricated in a knitting machine into which a CNT aerogel sheet drawn from CNT forest, wrapped around continuous supply of SPX filament was fed. These fabrics exhibited breaking strains of 600% to 900%, tensile strengths in the range 75 to 86 MPa and an approximate resistance of 3.0 kΩ m^−1^ [271]. They offer unique advantages such as large tensile actuation, high repeatability, scalability and stretchability due to which they can be used in various applications like medical sensors/devices and smart clothing.

### Alignment/Placement on Substrates

Most methods of CNT mass production result in randomly oriented CNT bundles. The placement and alignment of the tubes can either be done while they are being grown or after they have been grown. In the former, the CNTs are grown in a given direction and position within the substrate or normal to it, while in the post-growth method, the tubes are first isolated in a dispersion and then the dispersed CNTs are aligned and selectively positioned in a certain direction by applying external forces. Various methods have been used for aligning and placement of CNTs on substrates, as discussed below.

#### Photolithography

**Fig. 39:**
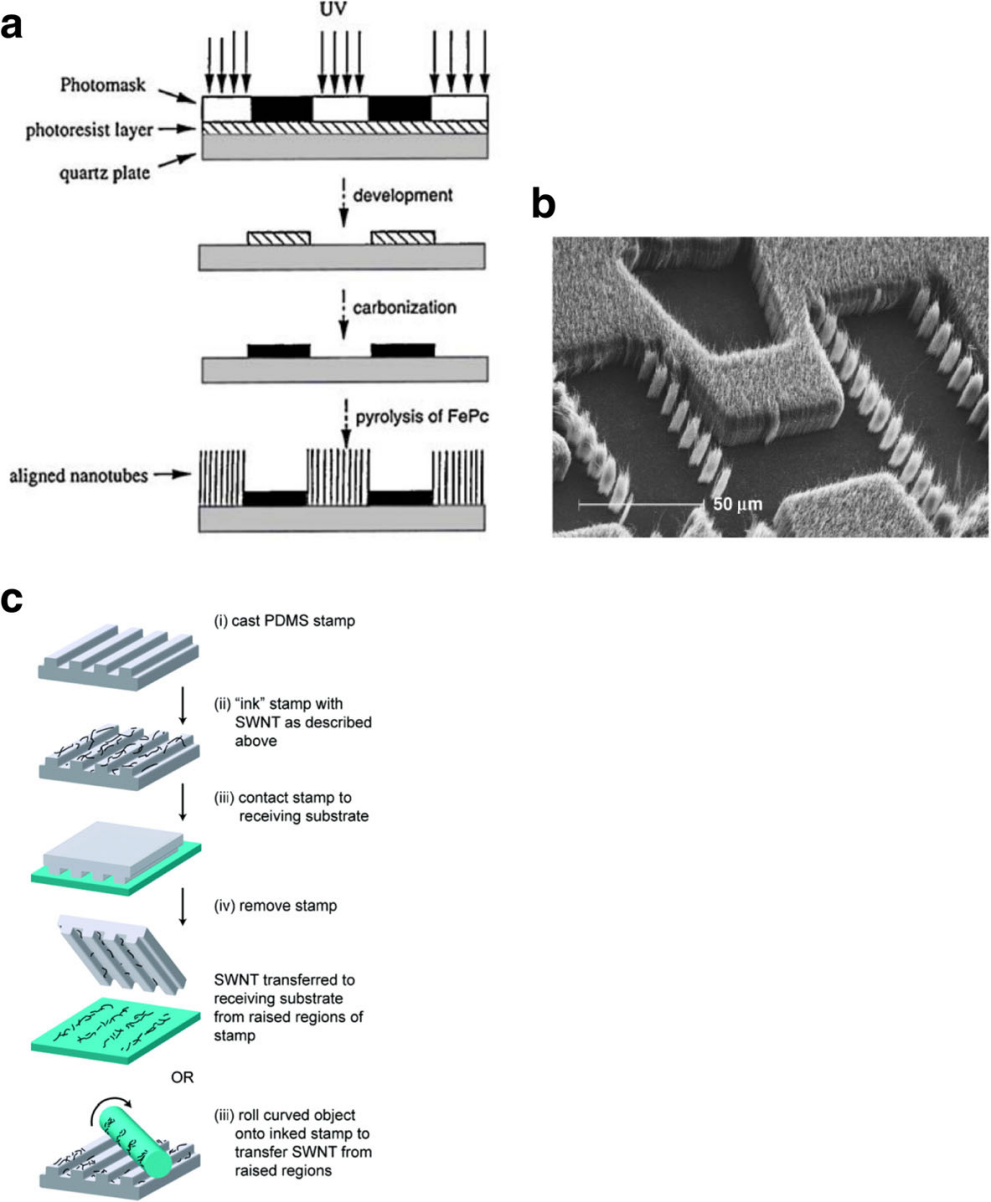
**a** Schematic flow diagram of micropattern formation of vertically aligned MWCNT arrays by direct photolithography process. Adapted from [272]. **b** SEM images of patterned films of vertically aligned MWCNT arrays formed by the pyrolysis of FePc onto a photolithographically patterned quartz substrate. The resolution of the figure is in the micrometer scale. Adapted from [272]. **c** Step-by-step illustration of transfer printing of SWCNTs onto various substrates by contact stamping. Adapted from [273]

Photolithography has been successfully used to prepattern either catalysts or substrates for site-selective growth of CNTs. In such a case, the photolithography setup consists of a substrate (glass), a patterned resist and a photomask. The process involves the following steps; first, direct photolithographic patterning is performed on the metal-containing photoresist. Next, metal oxides are heated and then reduced by hydrogenation to patterned metal nanoparticles, which act as catalysts from which aligned CNTs are grown, via the hydrocarbon pyrolysis of FePc (Fig. 39a, b) [272]. Alternatively, catalyst patterning using photolithography can be done by creating a template with desired patterns of photoresist, followed by molding of PET stamps which are used in transfer printing of CNTs. However, this technique suffers from certain limitations which include not having the necessary resolution to grow nanometer-scale patterns. In addition, growth of CNTs may be hindered/affected due to contamination that may be introduced through these masks [195].

#### Transfer Printing

Currently, most nanoelectronics devices are being made on flat, rigid, smooth surfaces. This poses a problem because future nanoelectronics applications will require for nanomaterials to be placed on flexible substrates, such as plastic, or nonplanar materials. Due to this, methods such as transfer printing, which allow for the fabrication of a device on a conventional substrate and then transfer-printing of the entire working device onto target substrates, are being developed [273, 274]. This precludes the need for performing harsh synthesis and fabrication methods on the target substrate. The mechanism for transfer printing CNTs is as follows; first, the tubes are stabilized by mixing them with surfactant molecules, next the surfactant-stabilized CNTs are deposited on a variety of substrates from the solution. The most common process for the deposition of the tubes with wide area coverage is based on controlled flocculation. The controlled flocculation approach uses a spinning mechanism, in which the suspension of tubes and a solvent (methanol) are applied at the same time, to a spinning substrate. The methanol or any other hydrophilic solvent displaces the surfactant from the nanotubes, by bonding to them thereby causing the tubes to precipitate from the solution, to the edges of the substrate where they are deposited on a high-resolution polydimethylsiloxane (PDMS) stamp. The tubes form thick films on the PDMS stamps, and the stamp can be pressed on the target substrate to transfer the tube patterns from the raised regions of the stamp (Fig. 39c) [273]. One problem with this technique is the requirement to pattern the stamps each time based on the design [275].

#### Template-Based Deposition

**Fig. 40:**
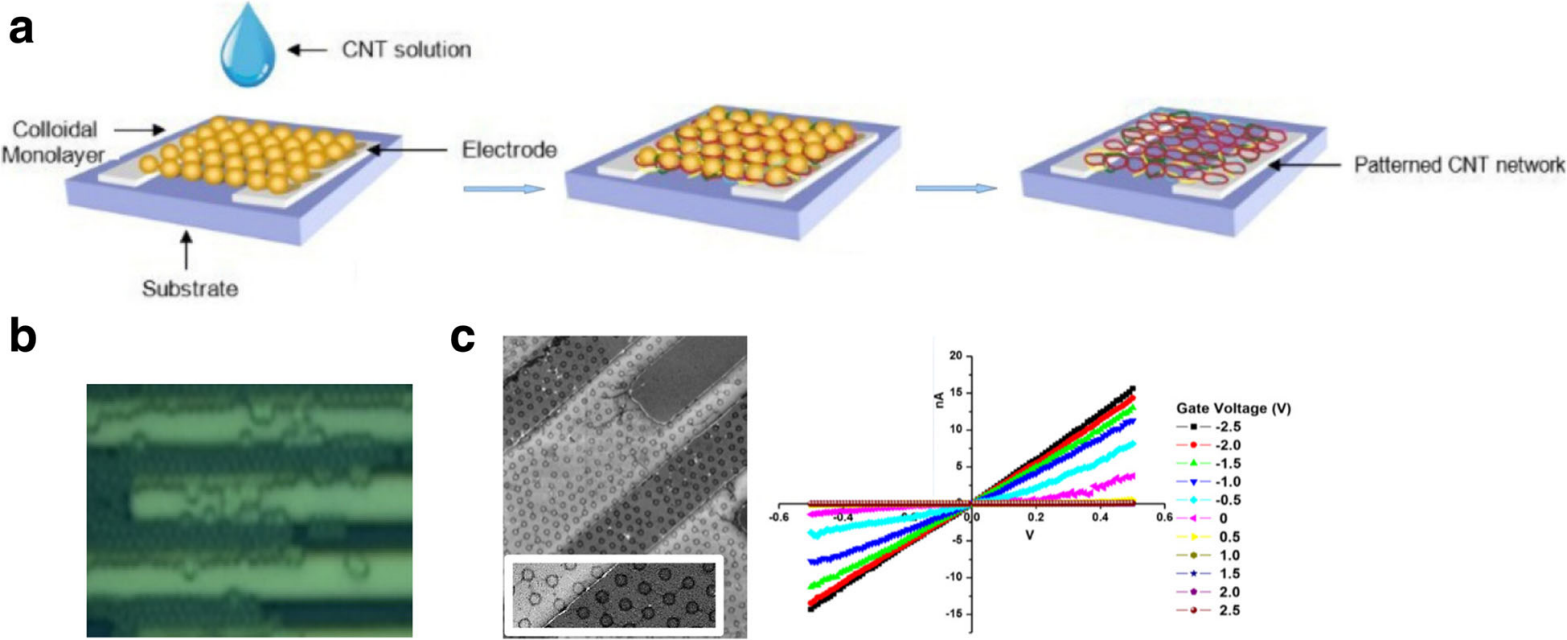
**a** Schematic of fabrication process for patterned CNTFET networks. **b** Optical microscope image of patterned electrode substrates after self-assembled colloidal mask assembly. **c** SEM image of interconnected nanotube ring patterns formed during the self-assembly process and gate voltage-dependent *I*-*V* characteristics of resulting CNTFET. The center to center distance of the circles is around 500 nm. (Inset) shows the corresponding magnified view of the interconnected nanotube ring patterns. [Y. Chen and C. Papadopoulos, unpublished results]

Patterning in thin film CNTFET channels can be achieved with an inexpensive solution-based self-assembled colloidal mask that leads to devices with large on/off ratios while maintaining carrier mobility and sub-threshold performance. By guiding SWCNT thin film formation, the colloidal mask approach allows an ordered nanoscale CNT network with more consistent and accessible channel surface area resulting in patterned thin film FET channels whose structure and properties can be tuned for different applications (such as sensors). In one of the template-based deposition techniques, the patterned SWCNT thin films were fabricated using colloidal lithography [241]. This method employed a liquid-based self-assembly of colloidal sphere monolayers as masks to create ordered nanoscale arrays and films. Here, heavily doped silicon wafers with ~ 100-nm-thick SiO_2_ layer was used as a substrate and patterning of Au/Ti source-drain electrodes with ~ 40 nm thickness and 1–3 μm spacing on the surface of wafer was carried using photolithography. SWCNT powder (1.2–1.5 nm diameter and 2–5 μm length) was dispersed in chloroform with SDS surfactant to form aqueous suspensions, which was mixed with an aqueous suspension of silica or polystyrene colloidal spheres to form patterned nanotube networks. The overall process flow for patterning the SWCNT thin films and the corresponding results are illustrated in Fig. 40a–c.

**Fig. 41:**
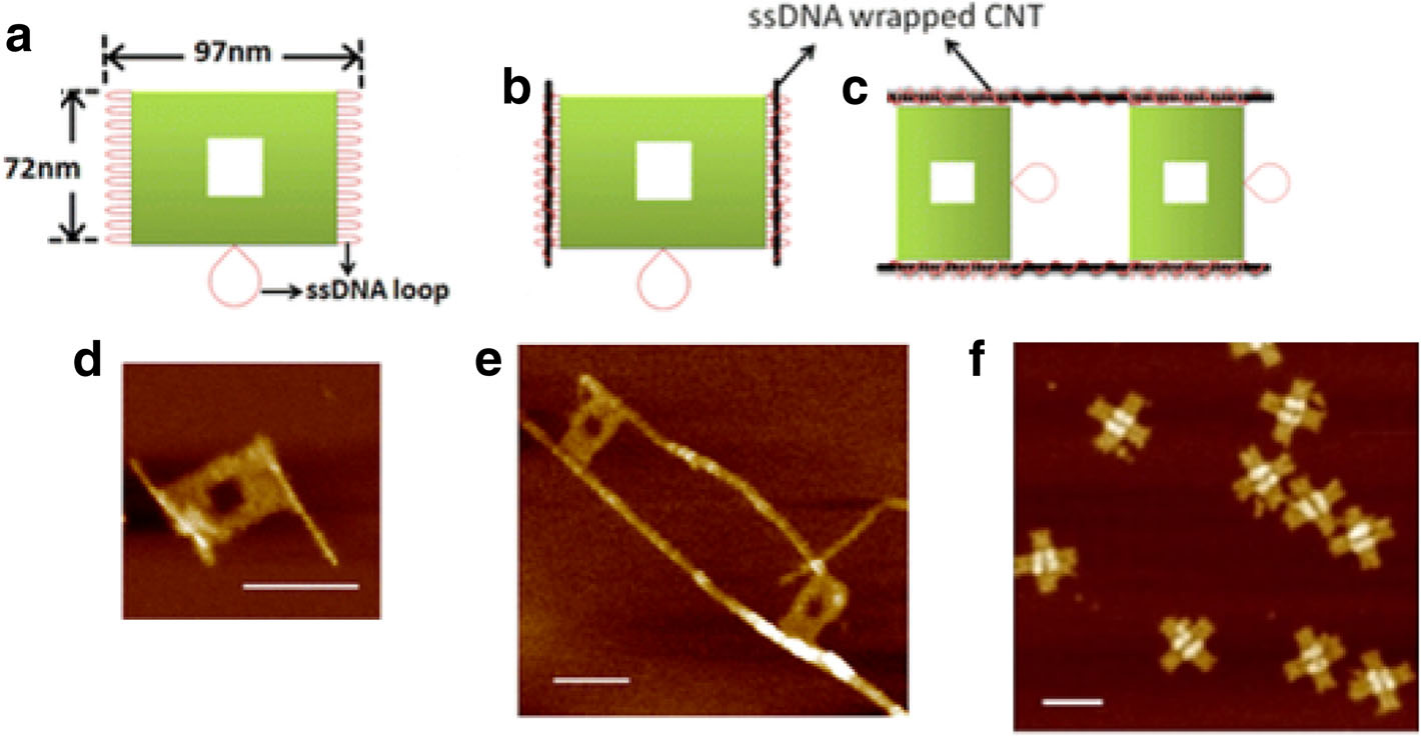
Schematics of **a** rectangular DNA-origami. Adapted from [276]. **b**, **c** Site-specific immobilization of ssDNA-wrapped short and long CNTs onto the single rectangular DNA-origami scaffold. Adapted from [276]. **d** AFM images of DNA origami-nanotubes formed using self-assembly showing CNTs aligned at the edges. Adapted from [276]. **e** AFM images of DNA origami-nanotubes formed using self-assembly showing two single rectangular DNA-origamis aligning two long 6,5-SWCNTs. Adapted from [276]. **f** Cross-shaped SWCNTs. Scale bar equals 100 nm. Adapted from [276]

Another technique to control the placement, alignment and spacing of CNTs is achieved using DNA-origami structures [276–278]. Here, various geometrical shaped (L-, T-, Y-, rectangular, triangular, etc.) nanotube structures are formed on DNA-origami templates using self-assembly process (Fig. 41), where the CNTs were organized into several patterns, with control over the inter-tube angles. In one of the techniques, CNTs solubilized by wrapping with ssDNA reacted with the DNA origami constructs forming linear arrays of ssDNA that lead to immobilization of the CNTs onto the DNA origami scaffold. This was due to the strong π–π interaction existing between the bases of ssDNA and CNTs. SWCNTs with lengths ranging from ~ 92 ± 24 nm (termed as short SWCNTs) and ~ 314 ± 249 nm (termed as long SWCNTs) were obtained in this work [276].

#### Solution-Based Deposition of CNTs

Most solution-based alignment or deposition methods of CNTs require the formation of CNT films from CNT suspensions. As such, there’s a need to form uniform dispersions of CNTs in a solvent. A challenge that is commonly encountered in dispersing CNTs is overcoming the strong inter-tube interaction of CNTs, which is attributed to van der Waals forces. When CNTs are placed in a solvent, they usually bundle together. This attractive force of the CNTs is usually overcome by binding the CNT walls to surfactant molecules (surfactant wrapping) to form stable solutions of CNTs. As such, these van der Waals forces are a major consideration when using solution-based methods for aligning CNTs [230]. Some of the solution-based alignment techniques are discussed below.

Inkjet printing is a precise method of patterning, and therefore, post-printing steps are not needed. This technique is advantageous because it does not require the use of a prefabricated template, thereby allowing for rapid printing at a low cost [279, 280]. Additionally, it is valuable because many layers of ink can be printed on top of one another. The carbon nanotube ink is prepared following certain steps; first, sonication is used to disperse the CNTs within the liquid. After dispersing the CNTs, the ink is centrifuged several times to separate the well-dispersed CNTs from the bundles, which could clog the printer nozzle. Next the supernatant is collected and filtered severally to remove any remaining CNT bundles. After the ink has been successfully produced, it is loaded into an inkjet cartridge and ready to be used for printing on substrates like glass and polymers.

**Fig. 42:**
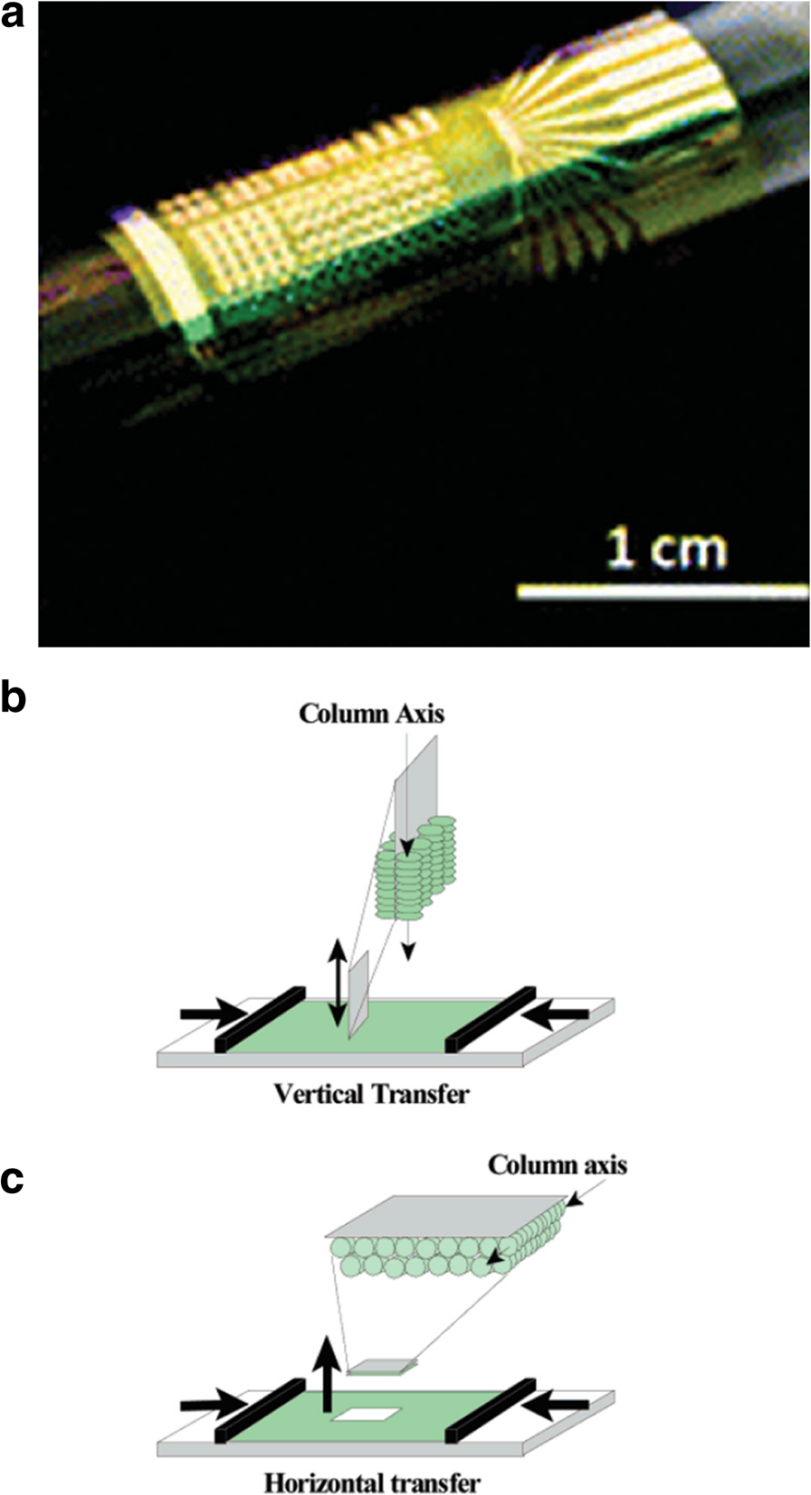
**a** Optical image of a CNT-based circuit on polyimide wrapped around a test tube. The circuit was fabricated using CNT inkjet printing. Adapted from [281]. **b** Schematic of the Langmuir-Blodgett (LB) process for the fabrication of films. The LB process is either performed using the **b** vertical dipping method or **c** horizontal lifting method. Adapted from [283]

Consumer inkjet printers are of two types; continuous and drop-on-demand. The former supplies a continuous stream of ink droplets, which are charged once they leave the nozzle, and are then deflected by voltage plates, such that the applied voltage determines if the droplet will be deposited on the substrate, while the drop-on-demand printer functions in a different manner, in that, the printer only ejects ink when required [279]. Carbon nanotube inkjet printing has been successfully used to deposit MWCNTs and SWCNTs. It has also been used to fabricate transistors, sensors and electroluminescent devices. The process is a useful development in electronics because it allows direct printing of the CNTs to make a pattern or a circuit on a suitable substrate, thereby enabling us to control the transparency and resistance of the printed patterns (Fig. 42a) [281]. In order to produce uniform networks of CNTs by inkjet printing, the jetting conditions need to be properly tuned to generate correctly directed droplets. Another important consideration is the temperature of the substrate. The substrates need to be heated in order to reduce the drying time for the printed CNT solution. For manufacturing purposes, quick drying of the dropped ink is desirable. However, the temperature should not be too high as it would cause the droplet to evaporate once it is released from the nozzle. A constraint in using this process is the difficulty of dispersing nanomaterials within the ink as CNTs typically bundle together in a solvent, due to their attractive van der Waals forces. The bundling of tubes needs to be prevented as it could cause clogging of the inkjet nozzle [279].

Alternatively, spray-coating involves spraying CNTs dispersed in a suspension on a heated substrate such that every sprayed droplet undergoes pyrolytic decomposition when it reaches the hot substrate surface thereby forming a thin layer of CNTs film [282]. CNT films can be spray deposited on glass before being transferred to a flexible substrate or they could be directly sprayed onto the flexible substrate. Before the spraying of the CNTs can take place, the solution has to be prepared. First, CNTs in powdered form are dispersed into a solvent via sonication. The CNTs are usually either dispersed in an organic solvent or in a surfactant-based aqueous dispersion. Surfactant-based dispersion using SDS or carboxymethyl cellulose (CMC) is usually preferred because the surfactants can be rinsed off after the film is deposited, as these surfactants are soluble in water. The surfactant is first dissolved in water to form an aqueous solution, to which the CNTs are later added. This forms a surfactant based aqueous CNT solution. Sonication of the surfactant-based solution is then performed to evenly disperse the CNTs. Finally, the solution is centrifuged, and the supernatant is taken from the top to be used for deposition. Before spray deposition of the CNTs can occur, the substrate surface has to be treated and cleaned using solvents like acetone and isopropanol, followed by plasma cleaning. After treatment of the substrate, the colloidal suspension of CNTs is sprayed through an air atomizing spray gun, onto a heated substrate. The dispersing fluid (in this case, the surfactant) then evaporates due to the heating of the substrate to leave behind a uniform coating or layer of CNTs.

CNT films can also be produced using the Langmuir-Blodgett (LB) technique, in which a substrate is dipped in a solution containing well-dispersed CNTs, and then slowly pulled out either by horizontal lifting or by vertical dipping method as shown in Fig. 42b, c [283]. This technique produces a thin layer of CNTs aligned in the dipping direction on the substrate. The thickness of the CNT film is dependent on the concentration of CNTs in the solution, the pulling speed, and the number of dips. Generally, SWCNTs are dispersed in an amphiphilic polymer matrix, spread on a water surface [284]. This film is transferred to a substrate by dipping the substrate in it. The disadvantage of this method, as with most solution-based methods, is that the solution might require a solvent that is incompatible with some substrates, for example plastic.

**Fig. 43:**
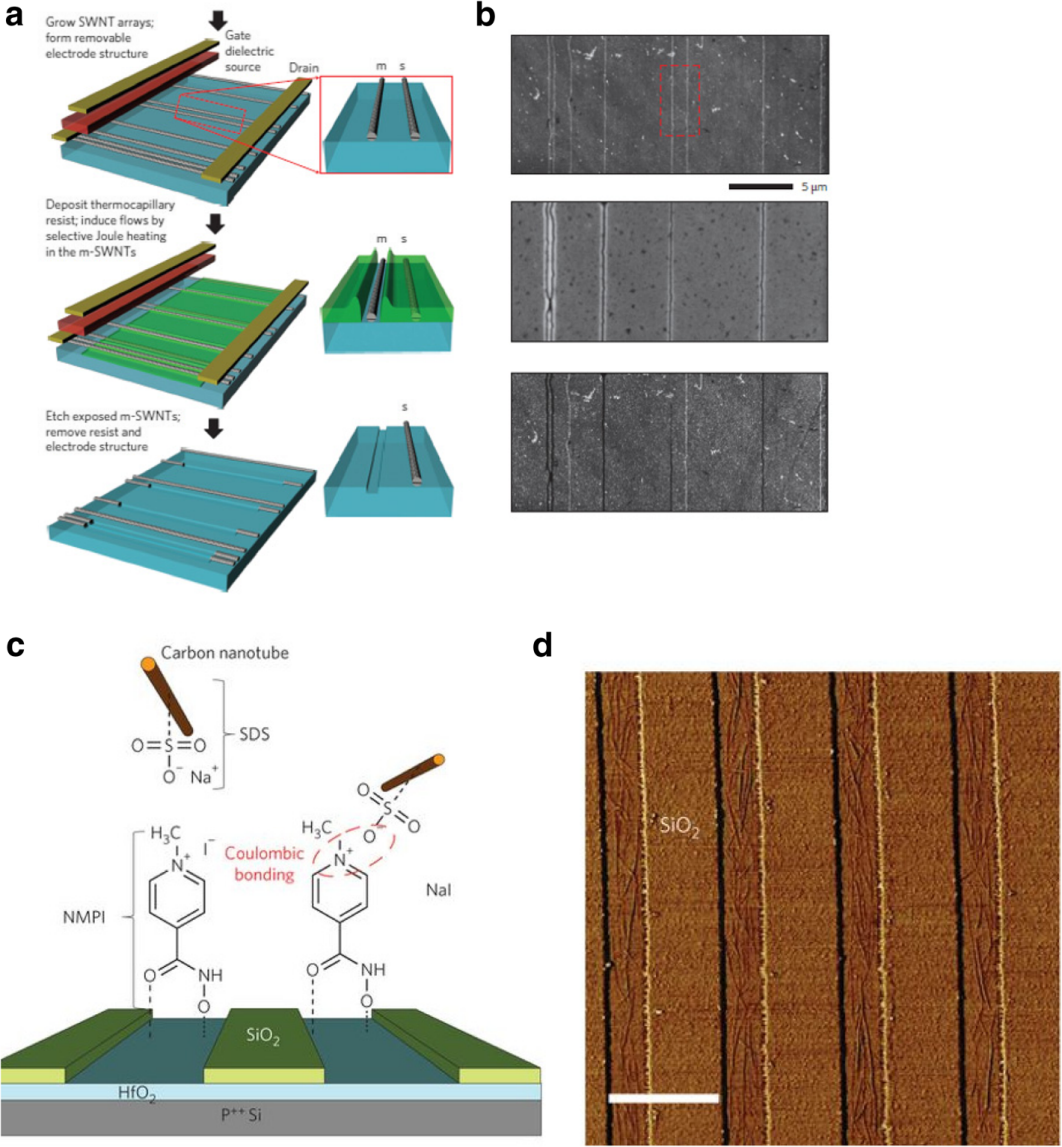
**a** Schematic illustration of steps involved in selective removal of metallic CNTs using thermocapillary effects. Adapted from [285]. **b** Corresponding AFM images of each step starting from growth of SWCNT arrays, followed by deposition of thermocapillary resist and etching of metallic SWCNTs. Adapted from [285]. **c** Schematic of the deposition of well-aligned CNTs on HfO_2_ by means of the ion exchange process. Adapted from [286]. **d** Corresponding AFM image of the nanotubes deposited on a HfO_2_ trench with a width of about 200 nm. Adapted from [286]

Well-aligned, horizontal arrays of semiconducting nanotubes can also be created using nanoscale thermocapillary effects in thin-film organic coatings (Fig. 43a, b) [285]. In this method, metallic SWCNTs are selectively removed via thermal resist exposure and etching. SWCNTs, grown on quartz substrates comprise of both semiconducting and metallic nanotubes with diameters between 0.6 and 2 nm. By means of thermal evaporation, thin organic thermocapillary resist layer of a,a,a′-tris (4-hydroxyphenyl)-1-ethyl-4-isopropylbenzene is deposited on these SWCNT arrays. Post this, by etching metallic CNTs are removed from the arrays.

Alternatively, aligned CNTs were deposited on a HfO_2_ trench with a density of 10^9^ cm^−2^ using self-assembly (Fig. 43c, d) [286]. This was done through the SDS-wrapped CNTs dispersed in water and the use of 4-(*N*-hydroxycarboxamido)-1-methylpyridinium iodide (NMPI) to form surface monolayer. This led to ion exchange between Na^+^ in SDS and I^−^ in NMPI. By controlling the concentrations of SDS surfactant and dimensions of the trenches, placement of CNTs inside the trench was randomly varied. With trenches of dimensions, 200 nm and 500 nm fabrication of a billion nanotubes per square centimeter is predicted. For example, one preliminary successful functioning CNT computer circuitry was self-assembled using selective surface chemistry [287].

## Emerging Applications and Challenges

**Fig. 44:**
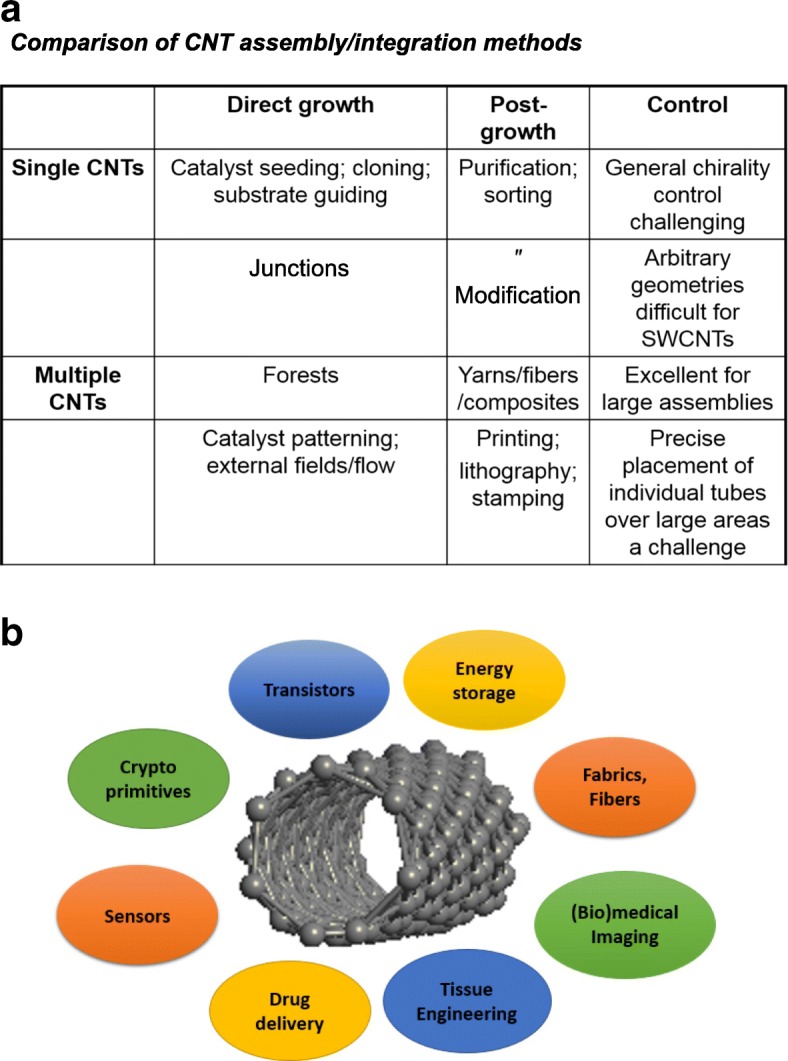
**a** Summary description of CNT assembly and integration techniques. **b** Overview of various emerging applications of CNTs

Figure 44a gives an overall summary and comparison of the various CNT assembly techniques presented in this review. As seen throughout the preceding sections, CNTs have gained widespread interest for use in applications [1, 39, 52, 57, 65, 66, 276, 288]. Electronic devices, sensors, drug delivery, energy storage devices, crypto primitives and tissue engineering are just some of the potential areas where nanotubes can be employed (Fig. 44b).

CNTs are used in various commercial applications in the form of conductivity enhancement/control in plastics [289], in anti-static packaging and to enhance the strength of concrete, polymers, baseball bats, bicycles, etc. [62, 289]. In addition, use of CNTs for structural monitoring in aircraft structures is currently patented [290]. Successful use of CNTs in this application may greatly reduce the risk of an in-flight failure caused by structural degradation of aircraft.

**Fig. 45:**
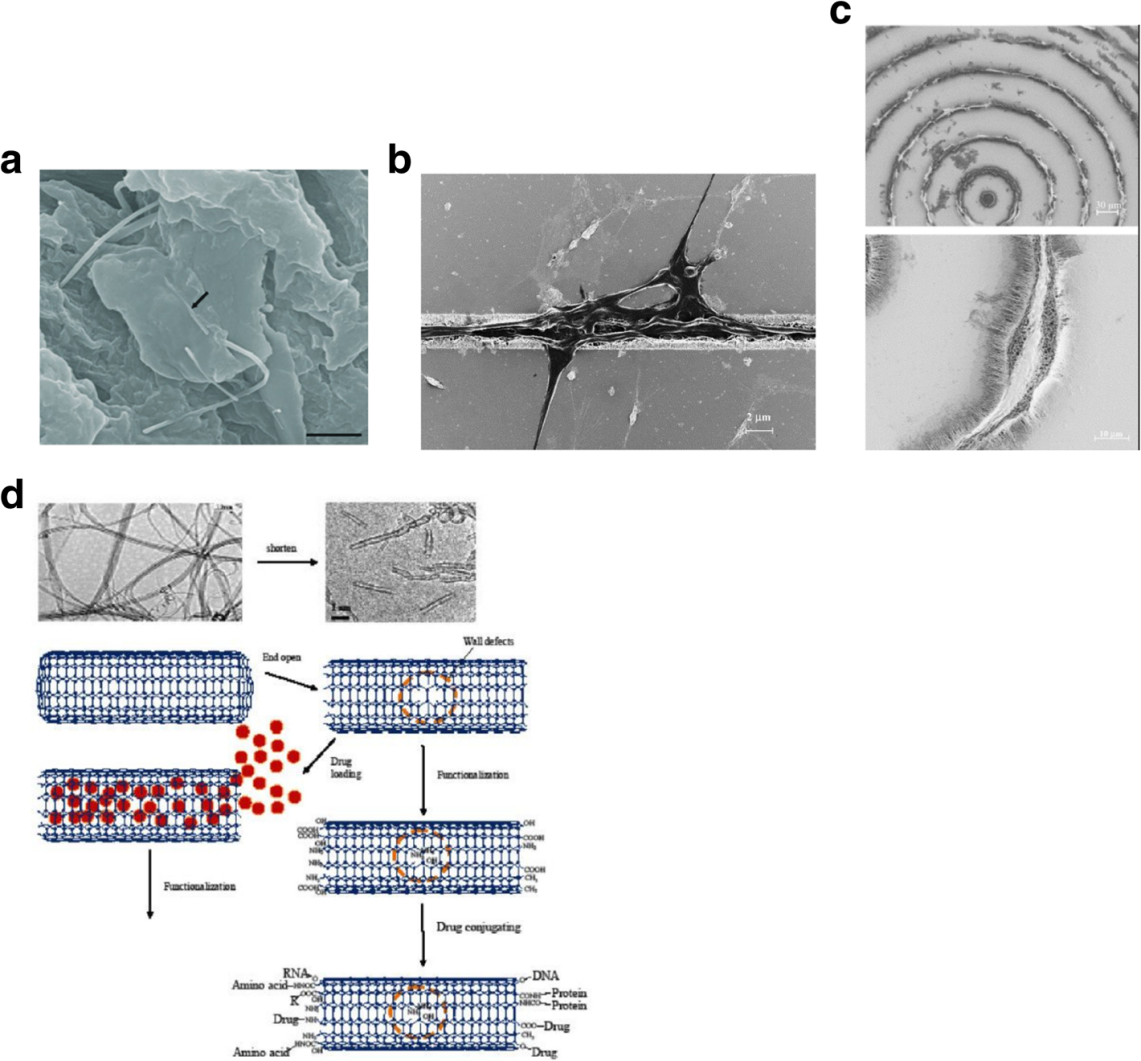
**a** SEM image of bone-tissue sections observed 4 weeks after CNTs were implanted into tubal defects in mice. Adapted from [295]. **b**, **c** SEM images showing guided neurite growth along the MWCNT array pattern. The bottom image in part (**c**) represents the magnified view of a selected region in the top image. Adapted from [296]. **d** Schematic of steps showing CNT-based drug delivery systems. CNTs of various lengths were used as drug carriers by making their ends open and allowing the drug to be loaded for targeted drug delivery. Adapted from [297]

CNTs are also finding use in biomedical applications such as imaging [291–293], tissue engineering as scaffolds for bone growth [294, 295] and scaffolds to guide neurite outgrowth [296] as shown in Fig. 45a–c. In addition, CNTs can also to be used in targeted drug delivery systems (Fig. 45d) [297, 298] due to their capabilities to interact with mammalian cells. Due to their ability to penetrate into cell membranes, CNTs can be used as carriers to deliver therapeutic agents into the cytoplasm of cells. CNTs that are gastrointestinally absorbed were lysosomotropic and could enter into the cells for targeted drug delivery.

**Fig. 46:**
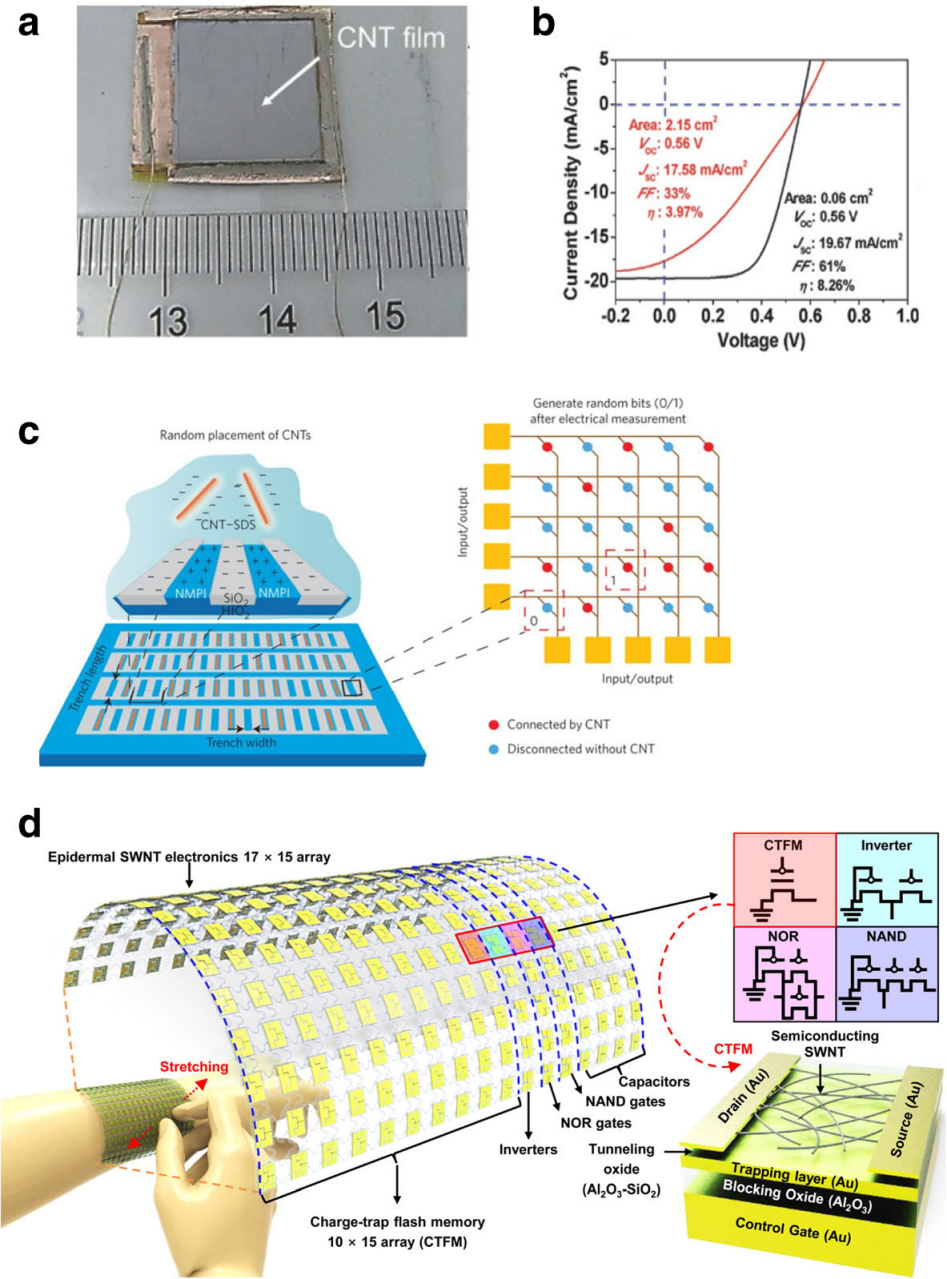
**a** Schematic of a thin-film CNT solar cell. Adapted from [308]. **b** J–V characteristics of a CNT solar cell with area = 2.15 cm^2^ (in red) and area = 0.06 cm^2^ (in black). Adapted from [308]. **c** Schematic of a randomly connected 2D nanotube array that can be used as a crossbar switch. Here, CNTs self-assemble in the HfO_2_ trenches with width of 70–300 nm. Adapted from [314]. **d** Schematic of an s-SWCNT-based wearable array of electronic devices, consisting of memory units, capacitors and logic circuits that can be integrated into different circuits for day-to-day applications. Adapted from [317]

As mentioned previously, a major class of applications of CNTs is in electronic devices like FETs [46, 48, 299] and to build integrated circuits (ICs) and flexible electronics [300, 301]. Some of the other applications include their use in batteries, where a CNT film was used as the current collector for a lithium ion battery [302], in transparent conductive films that can be used in flat panel displays such as laptops and cameras [57, 303], hydrogen storage to be used as fuel source [304], as interconnects [305, 306] that can potentially offer advantages over copper (due to their high thermal stability and large current carrying capacity), in non-volatile random access memory for molecular computing [307] and in thin-film solar cells made up of a semi-transparent thin film of nanotubes on a n-type crystalline silicon substrate, wherein CNTs films are used as photogeneration sites and are also used for enhancing conductivity (Fig. 46a, b) [308].

Due to their unique thermal and mechanical properties, CNTs have also found applications as nano additives in heat conductive materials made of CNT-based lubricating oils, nanoliquids, etc. [309] These additives can be used for heat dissipation in electronic devices like LEDs and computer processors [309–311]. The high thermal conductivity of CNTs also enables their use as thermally conductive composites that could potentially replace metallic parts in devices such as electric motors and generators [14, 312, 313].

In addition, CNTs have also been tested to create an unclonable electronic random structure to create cryptographic keys that can be a used to provide significantly higher level of security as compared to the conventional binary-bit architecture with the same key size [314, 315]. A schematic of generation of random bits based on two-dimensional (2D) carbon nanotube arrays is shown in (Fig. 46c). In this method, well-aligned CNTs are deposited on a HfO_2_ trench, ~ 7 nm high using self-assembly. This was done through the SDS-wrapped CNTs dispersed in water and the use of NMPI to form a surface monolayer, which led to ion exchange between Na^+^ in SDS and I^−^ in NMPI. By controlling the concentrations of SDS surfactant and dimensions of the trenches, placement of CNTs inside the trench was randomly varied. Further, CNT thin films, also known as CNT mats, have shown potential to be used in radio-frequency signal transmission [316]. A combination of most of the above applications includes the use of CNTs in wearable electronics consisting of many memory units, capacitors, transistors and logic circuits, etc. (Fig. 46d) [317]. In one of the recent examples, stretchable and ultrathin wearable array of electronic devices with a dimension of ~ 3 μm showed a possibility to be integrated on the human skin. Techniques such as spin coating, thermal evaporation and photolithography were used to fabricate these devices based on the required application.

Use of aligned/organized CNTs in the form of yarns, forests and sheets can help in further development of CNTs for large-scale industrial applications. Some of the emerging applications of CNTs include their use as multi-functional coating materials, which in turn can be used as an alternative to environmentally hazardous paints containing biocides [288]. CNTs have also proven useful in anticorrosion coatings in metals (showing enhanced strength and coating stiffness) [318], and in transparent electronics like flexible displays. Due to their ability to be manufactured as metallic, semi-conducting or wide-band gap CNTs, they can also be used in manufacturing new generation electronic components and devices like batteries, supercapacitors, transistors, logic circuits, memristors, neuromorphic computing, sensors for optics, gas, pressure, glucose, tumor detection, electronic textiles, artificial muscles and electro-thermal actuators, etc. [1, 219, 297, 307, 319, 320]. CNTs have a potential to be used in other applications in the form of wires in nanoscale very-large-scale integrated (VLSI) circuits [306], to fabricate flexible and foldable sheets [321], fibers via wet spinning [264, 322], in energy and water treatment [323].

Despite their numerous advantages and potential applications, several outstanding issues related to CNTs must still be addressed in order for their potential to be fully realized in the future (cf. Fig. 44a): For example, low mechanical strength and thermal conductivity of the typical as-grown CNT material has to be studied and improved. In addition, during the growth of long nanotubes tubes with closed ends are also formed due to the presence of pentagonal and heptagonal defects in the graphene lattice. This makes it difficult for their use in many applications where access to open-ended tubes is needed, e.g. to functionalize and/or contact the tube ends. Also, controlling chirality of the CNTs grown, which critically influences the electrical properties of the nanotubes is still considered a major challenge. Growing high purity, well-aligned semiconducting CNTs is one of the important steps for realization of various applications and thus far, growth of CNTs with very few metallic CNTs for thin films and devices is yet to be fully realized. Since the choice of catalyst plays a significant role in the growth of CNTs, further research on different materials that can be used as catalysts is necessary to understand issues arising due to the tube-catalyst interactions. A major obstacle to commercial implementation of CNT (or graphene) nanoelectronics is rooted in the lack of an efficient and reliable method of device production and integration (cf. silicon integrated circuits). In addition, CNT electronics will likely have to compete with the emergence of silicon nanowires and related structures for integrated circuits in the near future as the rapid pace of Moore’s Law continues along with evolution and advances in semiconductor electronics and CMOS technology. Continued focus on the other allotropes of carbon like graphene (for 3D CNT-graphene networks used in, e.g. thermal interfaces) and fullerenes is necessary which may help in wide scale production and/or improvement in the properties of existing CNTs. For applications requiring macroscopic amounts of nanotubes, well-ordered assemblies will allow optimization of desired properties with nanoscale control. Lastly, since CNTs have not yet been fully used in large-scale commercial applications, not much is known about their possible effects on the human health and the surrounding environment [324] both during processing, post-processing and disposal [325–327]. Studies focusing on these aspects will also be necessary.

## Conclusions

**Fig. 47:**
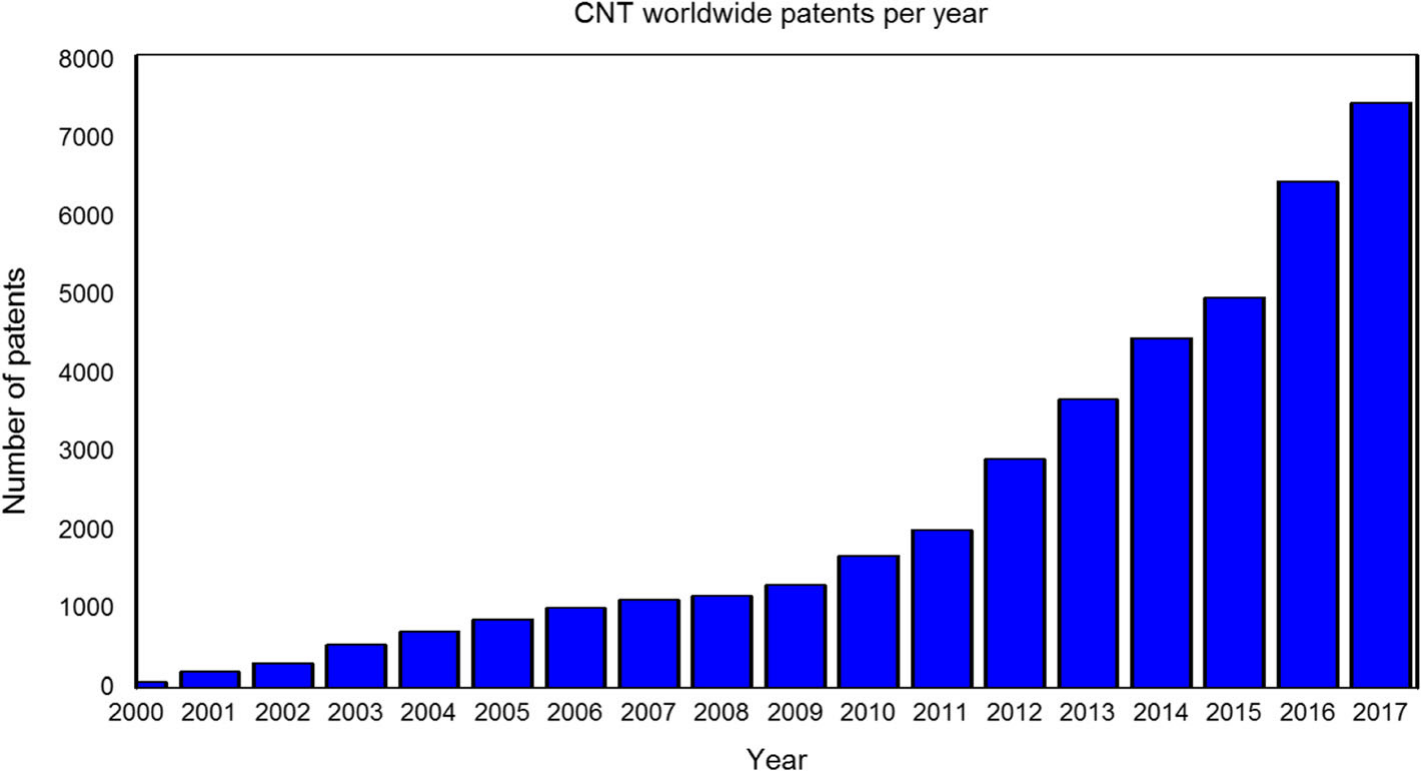
Trends in CNT-related patents filed worldwide between 2000 and 2017. Data collected from European Patent Office’s webpage using keywords ‘carbon nanotubes or carbon nanotube or graphene’

The superb (and unique) properties of CNTs and their potential use in a wide range of applications has led researchers to consider nanotubes based on carbon as one of the emerging materials that may play key roles in the future of nanoscale-based applications. This is supported by the CNT-related patent statistics between 2000 and 2017 (Fig. 47). In this review, we have focused on the progress made in the field of CNT fabrication, purification, assembly and integration of single and multiple tubes for their use in various applications. We discussed how structural control of individual CNT properties such as chirality, diameters and junctions are important factors in determining their properties (structural, electrical, mechanical, thermal properties and cost) and to utilize them for various applications. Also included is information related to post-growth purification techniques of CNTs using methods such as selective surface chemistry, gel chromatography and density gradient centrifugation. Assembly and integration for multiple CNTs using forest growth, catalyst patterning and composites are discussed. Details of their alignment/placement onto different substrates using methods such as photolithography, transfer printing and different solution-based techniques are also included. Towards the end, we list some emerging applications like sensors, field-effect devices, energy storage devices, health monitoring in aircraft structures, crypto primitives, commercial applications in the form of conductivity enhancement/control in plastics, in bio-based applications like tissue engineering, drug delivery, etc. based on their unique properties, advantages and challenges that need to be understood for efficient utilization of these structures in the future.

Significant progress has been made over the past two decades in CNT fabrication and assembly, from individual tubes to large ensembles and commercial applications should continue to grow as well-organized CNT materials are introduced. In terms of nanoscale integration, precise control of CNT type, placement and orientation will enable many applications in electronics and photonics and beyond, and despite challenges, continued advances in CNTs, with their almost ideal one-dimensional structure and properties, and related structures will play an important role in future applications of nanotechnology.

## Data Availability

Not applicable.

## Abbreviations

1D: One-dimensional
2D: Two-dimensional
3D: Three-dimensional
AC: Alternating current
AFM: Atomic force microscope
CB: Carbon black
c-CVD: Catalytic chemical vapor deposition
CMC: Carboxylmethyl cellulose
CNTFET: Carbon nanotube field-effect transistor
CNTs: Carbon nanotubes
CVD: Chemical vapor deposition
DC: Direct current
DEP: Dielectrophoresis
DGU: Density gradient ultracentrifugation
DNA: Deoxyribonucleic acid
DOS: Density of states
FCCVD: Floating catalyst chemical vapor deposition
FETs: Field-effect transistors
HDPE: High-density polyethylene
ICs: Integrated circuits
IEX: Ion-exchange chromatography
IR: Infrared
LB: Langmuir-Blodgett
LCDs: Liquid crystal displays
m-SWCNTs: Metallic single-walled carbon nanotubes
MWCNTs: Multi-walled carbon nanotubes
N-CNTs: Nitrogen-doped CNTs
NIL: Nano-imprint lithography
NMPI: 4-(N-hydroxycarboxamido)-1-methylpyridinium iodide
PBA: Prussian blue analog
PDMS: Polydimethylsiloxane
PECVD: Plasma-enhanced chemical vapor deposition
PET: Polyethylene terephthalate
PMMA: Polymethyl methacrylate
PTC: Positive temperature coefficient
PVA: Polyvinyl alcohol
PyC: Pyrolytic carbon
SDS: Sodium dodecyl sulfate
SEM: Scanning electron microscopy/microscope
SPX: Spandex
SSA: Specific surface area
ssDNA: Single-stranded-deoxyribonucleic acid
s-SWCNTs: Semiconducting single-walled carbon nanotubes
SWCNTs: Single-walled carbon nanotubes
TEM: Transmission electron microscope
THz: Terahertz
TPU: Thermoplastic polyurethane
UHMWPE: Ultra-high molecular weight polyethylene
UV: Ultraviolet
VHS: Van Hove singularities
VLS: Vapor-liquid-solid
VLSI: Very-large-scale integrated
VPE: Vapor phase epitaxy
VSS: Vapor-solid-solid
